# A general route to β,β-carbocyclic sidechains in peptides: an aqueous metallaphotoredox approach driven by green light

**DOI:** 10.1039/d5sc08845c

**Published:** 2026-02-05

**Authors:** Samuel Gary, Pei-Hsuan Chen, Nin Mai, Steven Bloom

**Affiliations:** a Department of Medicinal Chemistry, Gray-Little Hall Lawrence KS 66045 USA spbloom@ku.edu

## Abstract

Amino acids with β,β-carbocyclic sidechains are valuable replacements for endogenous Val, Leu, and Ile, with therapeutic benefits. When placed into ordinary peptides, these annulated variants improve metabolic stability, cell permeability, and receptor affinity and selectivity. Yet, their appearance in modern peptide drugs is often limited to β,β-cyclopentyl- and β,β-cyclohexyl-rings, one reason being the limited availability of resin- and solution-compatible β,β-carbocyclic amino acids for direct coupling. More ‘exotic’ rings, *i.e.*, those with different sizes, chemical compositions, and geometric preferences, could be superior, but finding and assessing their benefits calls for more general ways to incorporate and test them. Herein, we pioneer a modular route to convert a single unsaturated residue, known as β-sulfonyldehydroamino acid (ΔSulf), in a peptide into many unique β,β-carbocycles—cyclic, polycyclic, and heteroatom-containing—in two telescoped steps. First, an unprecedented photocatalyst, Pyronin Y, in an original combination with an organodiiodide, cobalt porphyrin catalyst, sacrificial amine, and green LEDs converts ΔSulf into a Δ-amino acid with a pendant iodide. Adding Zn/Cu couple then triggers an intramolecular and stereoselective Giese cyclization. We detail the mechanism of our procedure, highlighting the interplay between aqueous metallaphotoredox catalysis, halogen-atom abstraction, and ligand-controlled cyclization using spectroscopy, cyclic voltammetry, intermediate-trapping, and radical-clock experiments.

## Abstract 2

An approach to transform peptide β-sulfonyldehydroamino acid (ΔSulf) residues into chiral cyclic-β,β disubstituted amino acids is reported, combining aqueous metallaphotoredox catalysis, halogen-atom transfer, and ligand-controlled cyclization.

## Graphical abstract

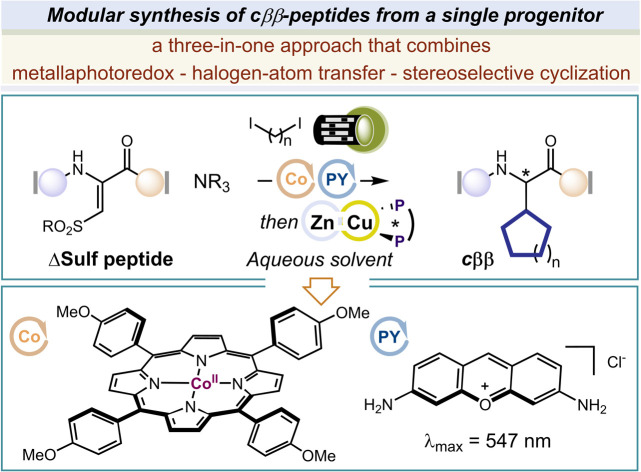

## Introduction

**Fig. 1:**
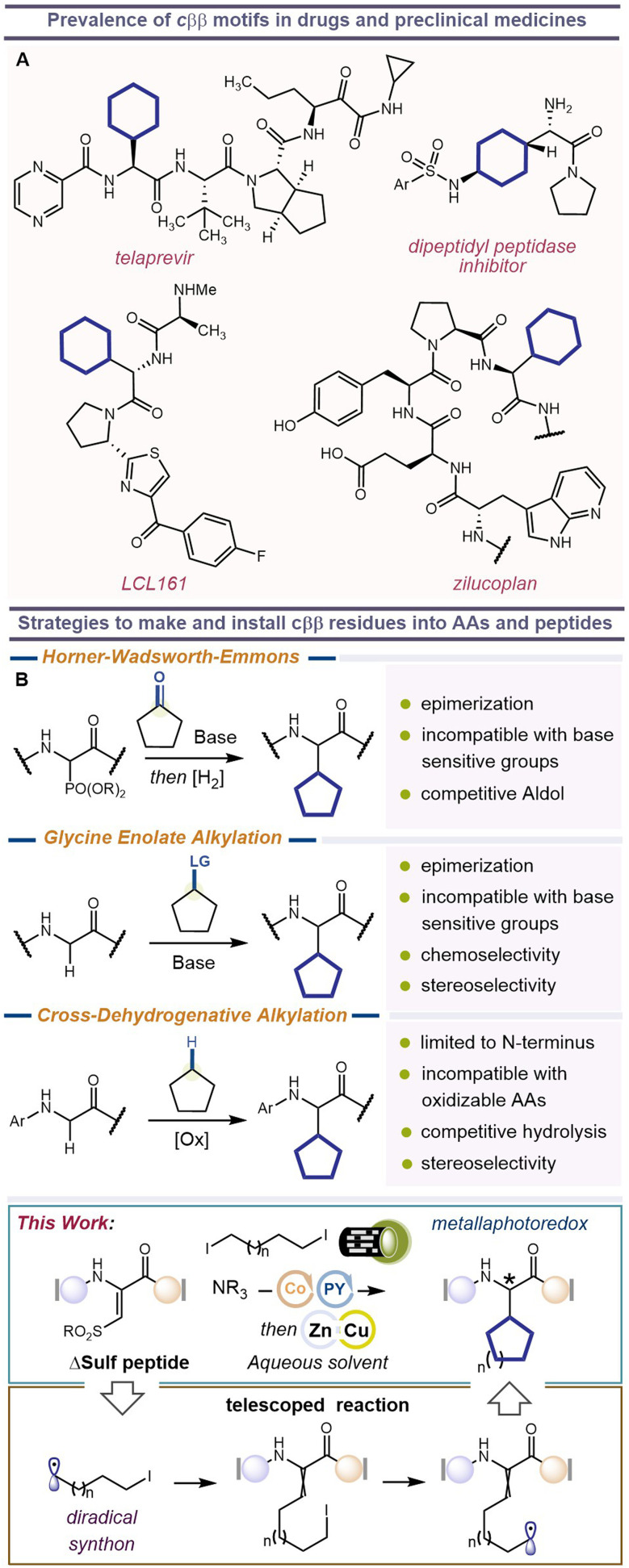
(A) Examples of β,β-cycloalkyl amino acids found in bioactive molecules. (B) Current methods used in the synthesis of β,β-cycloalkyl amino acid derivatives and this work.

Peptides are the third most abundant therapeutic modality—tailing behind biologics and small molecules—and have gained increasing traction in recent years.^1^ They are incredibly specific and highly potent, display broad chemical and biological diversity, are well-tolerated, and have outstanding therapeutic efficacy.^2,3^ Many of these salient features can be enhanced by introducing at least one Non-Proteinogenic Amino Acid (NPAA) into the peptide. These NPAAs can take many forms, including those that mirror endogenous residues (isosteric replacements), and those for which no biological counterpart exists. NPAAs that mimic endogenous residues often harbor additional atoms, groups, or stereocenters that promote non-covalent interactions—hydrogen-bonding, π-stacking, space-filling—to drive *in vivo* activity and therapeutic competence.^4,5^ One example is cyclic β,β-disubstituted (*c*ββ) amino acids. These synthetic residues are analogous to native Val, Leu and Ile, but their cyclic three-dimensional structure increases their effective size, conformational diversity, and metabolic and plasma stability.^6–9^ Unsurprisingly, *c*ββs have found their way into several FDA-approved peptide drugs, including epelsiban/retosiban,^10,11^ zilucoplan^12^ and telaprevir,^13^ as well as pre-clinical dipeptidyl peptidase inhibitors^8^ and anti-apoptotic agents,^14^Fig. 1. Some peptides even incorporate multiple *c*ββs, such as SEN304, which mimics the hydrophobic core of the Aβ42 peptide (LVFFL) and has been investigated as a possible treatment for Alzheimer's disease.^15^ But these examples are predominately limited to commercial *c*ββs with cyclopentyl- and cyclohexyl-rings. Other rings may be superior, but their lack of commercial availability prevents them from being sampled and, ultimately, adopted into peptide drugs. Standard approaches to insert *c*ββs into peptides rely on Horner–Wadsworth–Emmons (HWE) reactions between phosphonoglycine (Schmidt's reagent) and designer ketones,^16,17^ electrophilic alkylation of glycine residues,^18,19^ or redox-mediated cross-dehydrogenative coupling on *N*-arylglycine residues.^19–23^ Although moderately successful, these methods have limitations including poor chemoselectivity when using peptides with acidic or oxidizable sidechains, risks of α-epimerization, and inability to be performed at interior positions in the peptide, Fig. 1. Therefore, most *c*ββs are prepared in a separate chemical synthesis and then integrated into the nascent polypeptide using liquid-phase or solid-phase peptide synthesis. To accelerate *c*ββ peptide synthesis and discovery, it would be better if a single progenitor residue could first be positioned in the peptide and then transformed, *in parallel*, into a bevy of new *c*ββs, yielding a comprehensive inventory of peptides for biochemical testing. Such an approach would not only streamline hit-to-lead campaigns with *c*ββs but allow medicinal chemists to quickly customize a diverse set of probe molecules to map protein binding sites and elucidate complex structure–activity relationships. Hereafter, we describe our approach to addressing this timely challenge.

### Experimental design plan

We envisioned a divergent approach to *c*ββs using our recently reported β-sulfonyldehydroamino acid (ΔSulf).^24^ This new bioorthogonal reagent can undergo two successful C–C bond-forming events by intercepting two different C-centered radicals. The first C-radical displaces the sulfonyl group, while the second C-radical performs a Giese reaction to furnish the saturated β,β-disubstituted product. If we use a single reagent that can give rise to both radicals—a diradical synthon—then we could fashion an annulated product, *i.e.*, the desired *c*ββ, Fig. 1. It might also be possible to bias cyclization to deliver d-*c*ββs and l-*c*ββs in a stereoselective manner.

## Results and discussion

To explore our design plan, we selected a model tetrapeptide, AcNH-Gly-Pro-ΔSulfCb-Phe-CONH_2_ (1a), where the sulfonyl group of our dehydroamino acid is capped with a cyclobutyl (Cb) ring. We surveyed diiodobutane (diradical synthon) with twenty-eight photoredox catalysts, using 1 : 1 H_2_O : TFE as solvent and *N*,*N*-diisopropylethylamine (DIPEA) as a sacrificial amine. We also surveyed electrochemical conditions; see SI S11–S13. None of these afforded the 5-membered *c*ββ product (3a). The only noteable product was the β-alkylated dehydroamino acid 2a, uniquely formed with a green-light (525 nm) activated Pyronin Y (PY^+^) photocatalyst in 15% conversion. 2a is produced when the first radical is formed and displaces the sulfonyl group, but the second radical that closes the ring through intramolecular conjugate addition is not generated. The formation of 2a could not be enhanced by substituting the organodiiodide with other diradical synthons, *e.g.*, 1,4-Katritzky salts, -*N*-hydroxyphthalimide esters, and -dibromides; see SI S14. Because the second C–I bond remains intact, it is still possible to forge the annulated *c*ββ product from 2a through conjugate addition of the newly tethered alkyliodide onto the adjoining π-system. We found that this could be done by adding Cu(acac)_2_ and Zn powder to the crude reaction mixture,^25^ delivering the desired *c*ββ; a two-step approach done in a single reaction vessel without purification of the intermediate.

The combination of PY^+^ and DIPEA was further optimized for *c*ββ formation under 525 nm irradiation. One immediate challenge was the low conversion to the intermediate β-iodoalkyl-dehydroamino acid (15% conversion). A possible reason for this poor conversion is that primary alkyl radicals—from our organodiiodide—are highly reactive and prone to side reactions, *e.g.*, H-atom abstraction, intramolecular cyclization, and homodimerization.^26–28^ Adding a transition metal salt could sequester the alkyl radical and help guide the first addition step (sulfonyl displacement) through an organometallic mechanism. Including a cobalt porphyrin, Co^II^ [tetra(*p*-methoxyphenyl)]porphyrin (Co^II^TMPP), nearly doubled our conversion (29%). This finding is significant not only for its ability to improve our yield, but that synergistic reactions involving metals and photocatalysts are incredibly rare in water, with only a handful of examples being reported.^29–31^ However, Co^II^ also encouraged the conjugate addition of TFE to ΔSulfCb. Switching the cosolvent to MeCN eliminated this background reaction and provided a small boost in conversion (30%). Decreasing the loadings of PY^+^ and Co^II^TMPP to 7 mol% and 1 mol%, respectively, further increased the conversion to 41%. At this point, we gathered that a change in either Co^II^ catalyst, amine base, or ΔSulf substrate would be needed to bolster conversions beyond 40%. A survey of electronically distinct porphyrin and phthalocyanine cobalt ligands revealed that our original Co^II^TMPP was optimal; see SI S16. Using tri-*n*-butylamine (NBu_3_) improved conversions to 53%. And finally, exchanging the cyclobutyl group of ΔSulfCb for an *n*-propyl group (ΔSulf^*n*^Pr, 1b) gave 56% conversion. Refinements in concentration, *i.e.*, moving from 2 mM to 4 mM and adding phosphate buffer (pH 7, 10 mM overall), provided an optimal and reproducible yield of 63% (4 : 1 Z : E geometric isomers), respectively. Key optimizations are summarized in Table 1. Even with these optimized conditions, we still did not observe the formation of 3a. Moreover, isolating 2a and resubjecting it to our optimized protocol failed to produce 3a, suggesting that our metallaphotoredox system does not induce cyclization. The only observable byproducts in our reaction were reductive cleavage of the β-sulfonyl group from 1b, reduction of the resulting dehydroalanine residue to saturated alanine, and hydrodehalogenation of 2a. As a final point of interest, we checked the compatibility of our system with different amino acids, including aromatic (Phe), aliphatic (Leu), oxidizable (Trp, Tyr, Met), β-branched (Val), and basic (His, Lys) residues, using a series of dipeptides, *i.e.*, AcNH-ΔSulfCy-AA-CO_2_Me, where AA = amino acid. All residues except Lys and Met (oxidized to sulfoxide) were tolerated; avg. 41% for alkylation (step 1) and avg. 30% for cyclization (step 2). See SI S17–S23 for dipeptide results.

**Table 1:** Reaction optimization^a^

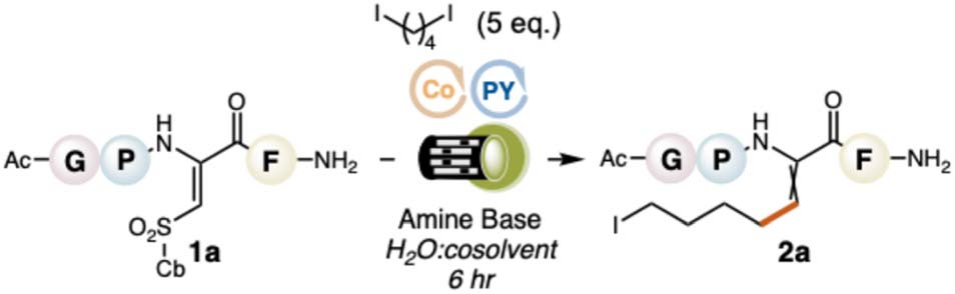					
Entry	Photocatalyst	Metal catalyst	Amine	Solvent	Conversion
1	PY^+^ (0.1 eq.)	—	DIPEA	H_2_O:TFE	15%
2	PY^+^ (0.1 eq.)	CoTMPP (0.1 eq.)	DIPEA	H_2_O:TFE	29%
3	PY^+^ (0.1 eq.)	CoTMPP (0.1 eq.)	DIPEA	H_2_O:MeCN	30%
4	PY^+^ (0.07 eq.)	CoTMPP (0.01 eq.)	DIPEA	H_2_O:MeCN	41%
5	PY^+^ (0.07 eq.)	CoTMPP (0.01 eq.)	NBu_3_	H_2_O:MeCN	53%
6^b^	PY^+^ (0.07 eq.)	CoTMPP (0.01 eq.)	NBu_3_	H_2_O:MeCN	56%
7^b^^,^^c^	PY^+^ (0.07 eq.)	CoTMPP (0.01 eq.)	NBu_3_	H_2_O:MeCN	56%
**8** b ^,^ c	**PY** ^**+**^ **(0.07 eq.)**	**CoTMPP (0.01 eq.)**	**NBu**_**3**_	**Kpi:MeCN**	**63%**
9^b^^,^^c^	PY^+^ (0.01 eq.)	—	NBu_3_	Kpi:MeCN	32%
10^b^^,^^c^	PY^+^ (0.07 eq.)	CoTMPP (0.01 eq.)	—	Kpi:MeCN	0%
11^b^^,^^c^	—	CoTMPP (0.01 eq.)	NBu_3_	Kpi:MeCN	0%

a Reactions performed with ΔSulfCb and 5 eq. of amine base at [2 mM] unless stated otherwise.

b Reaction performed with ΔSulf^*n*^Pr.

c Reaction performed at [4 mM].

**Fig. 2:**
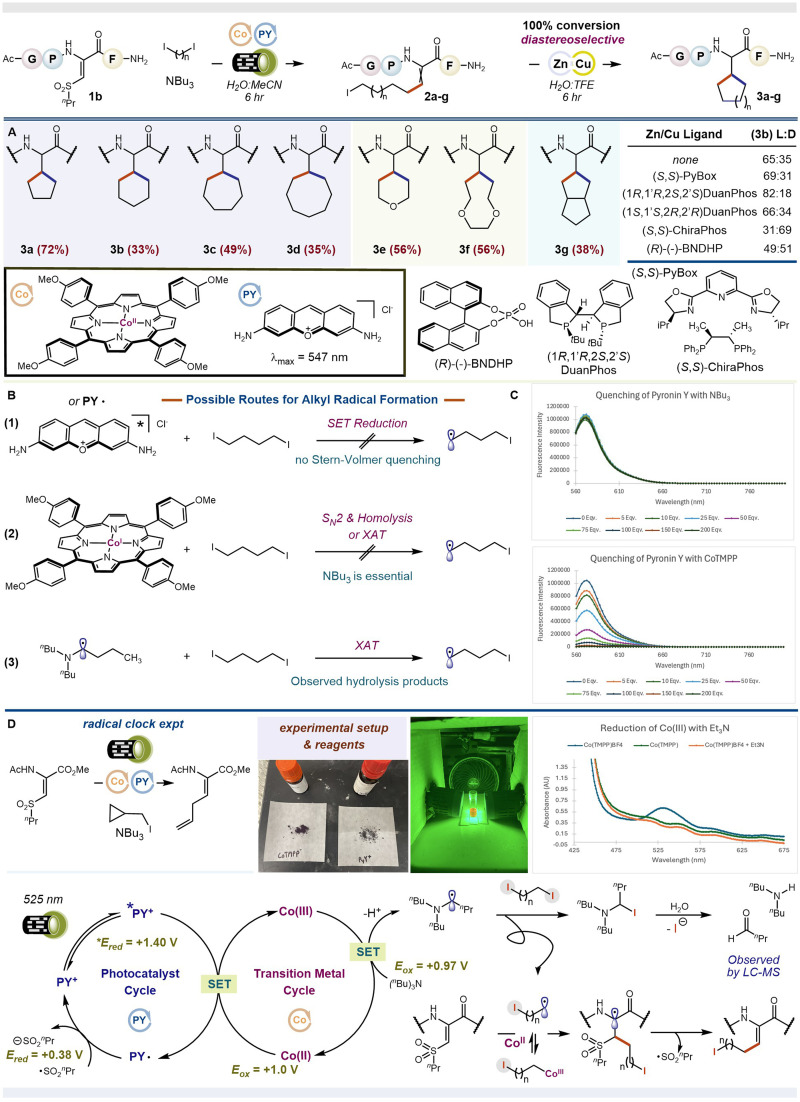
(A) Reaction conditions, substrate scope, and the screening of chiral ligands. Percentage values refer to the ratio of LC-MS peak area of products to all other peptide-derived products and starting material at 214 nm from the first step. (B) Mechanistic postulates for the generation of C centered radicals from diiodides. (C) Stern–Volmer fluorescence quenching indicates Co^II^TMPP is the best reaction component for quenching the fluorescence of Pyronin Y. (D) Proposed mechanism for the generation of 2a–g.

We examined the scope of our reaction, Fig. 2A. We found that ring sizes of 5-, 6-, 7-, and 8- (compounds 3a–d) could be fashioned from the corresponding diiodides using our standard peptide and optimized metallaphotoredox conditions, followed by addition of Cu(acac)_2_ and Zn powder to the crude reaction mixture. We found that including TFE could accelerate the ring-closing step, affording complete (100%) conversion in 6 h. We also found that one of the two possible diastereomers from ring closure is favored. By comparison to authentic samples, we deduced that our reaction favors the l-diastereomer (dr 65 : 35); See SI S75–S78. This innate bias could be enhanced when a chiral ligand, *e.g.*, (1*R*,1′*R*,2*S*,2′*S*)DuanPhos, was coordinated to Cu(acac)_2_ in the second step on the crude reaction material; shown for compound 3b (dr 82 : 18). However, using the opposing (1*S*,1′*S*,2*R*,2′*R*)DuanPhos enantiomer failed to produce the opposite peptide diastereomer, indicating a matched *vs.* mismatched case, respectively. Using a different ligand altogether, *i.e.*, ChiraPhos, flipped the selectivity to favor the d-diastereomer (dr 31 : 69), overriding the innate preference of the peptide substrate.^32–34^ (comparable diastereoselectivities were obtained when using purified 2b in place of the crude mixture). Bidentate and tridentate amine-based ligands did not influence the stereochemical outcome of our reaction and chiral Brønsted acids tended to give racemic mixtures. A more comprehensive survey of chiral ligands is included in the SI, see S24–S31. We plan to examine other phosphine-based ligands in future reports, including chiral variants thereof. Diiodides that form heteroatom-containing *c*ββs also worked (compounds 3e and 3f) as did ones that yield bicyclic *c*ββs (compound 3g). In all cases, the peptide products could be purified by routine flash chromatography in quantities suitable for early-phase medicinal chemistry projects (>0.1 mg, with purities exceeding 65%), as previously shown by our lab.^35^ In fact, we tested a few of our products as inhibitors of Aβ42 fibril formation, but they were not effective.

### Mechanistic studies

To unravel the synergistic effect between PY^+^ and Co^II^TMPP, a series of mechanistic experiments were performed. First, we conducted a radical clock experiment to probe the involvement of free radicals in our reaction. ΔSulf^*n*^Pr was reacted with 1-iodomethylcyclopropane in the presence of PY^+^, Co^II^TMPP, and NBu_3_. Loss of an iodine atom generates a primary carbon-centered radical that unfurls the cyclopropane ring, forming an intermediate homoallylic radical (*k* ∼10^8^)^36^ that can conjugate to ΔSulf^*n*^Pr. Addition of a new alkene fragment to ΔSulf^*n*^Pr can, therefore, be taken as evidence for the involvement of a transient alkyl radical. Indeed, we observed the ring-opened olefin product, Fig. 2D & SI S64. Including 3 equivalents of TEMPO, a reagent commonly used to trap free radicals, abolished all reactivity. Together, these initial results show that our metallaphotoredox conditions turn C–I bonds into free carbon-radicals that engage ΔSulf^*n*^Pr through open–shell conjugate addition.

How does the carbon-radical form from the diiodide? We envisioned three scenarios involving PY^+^ (*λ*_max_ 547 nm) and Co^II^TMPP (Q_1_ band *λ*_max_ 548 nm), both of which absorb the green light used in our standard reaction, Fig. 2B: (1) SET reduction of the organodiiodide by excited state *PY^+^ or ground state PY˙, the latter being formed *via* reductive quenching of *PY^+^ by sacrificial NBu_3_ or Co^II^TMPP, (2) S_N_2-like oxidative addition of Co^I^TMPP into the C–I bond followed by photolytic cleavage. The requisite Co^I^ species is formed from Co^II^*via* oxidative quenching of *PY^+^ or reduction by NBu_3_, and (3) iodine-atom abstraction by neutral Bu_2_NC·H(CH_2_)_2_CH_3_, formed from oxidation of NBu_3_ by *PY^+^ or *Co^II^TMPP. Stern–Volmer quenching is an excellent way to interrogate these three possibilities (Fig. 2C). For *PY^+^, no significant quenching was observed with NBu_3_, CH_3_(CH_2_)_3_I, or ΔSulf^*n*^Pr, the last study being done with and without NBu_3_. Even though quenching of the excited state photocatalyst by NBu_3_ may appear insignificant, the fact that our reaction works when only NBu_3_ and PY^+^ are present, as shown in Table 1, suggests that this process, common to many photoredox reactions,^37,38^ can still operate, leading to intermediate dehydroamino acid 2a when cobalt is not present. Only Co^II^TMPP significantly quenched *PY^+^, appearing linear at low concentrations of Co^II^TMPP and exponential at higher concentrations of Co^II^TMPP (≥100 equivalents). This change indicates that both dynamic (diffusion-controlled SET) and static (EDA complexation or ground state electron transfer) mechanisms are operative at high Co^II^TMPP concentrations.^39^ Because our reaction is performed with sub-stoichiometric amounts of Co^II^, only linear quenching should be considered further. This notion is also supported by the fact that no new spectral peaks appear when reaction quantities of Co^II^TMPP and PY^+^ are combined. In other words, no new complexes or catalytic entities are formed in the ground state. The ability of Co^II^TMPP to quench *PY^+^ suggests that Co^II^ can function beyond capturing and stabilizing a transient alkyl radical; it can function as a redox mediator. Interestingly, its fluorescence is also quenched by PY^+^, highlighting that an intimate redox pair exist between Co^II^TMPP and PY^+^ in the excited state. Note: the prior experiment required excitation of the Co^II^TMPP Soret band (*λ*_max_ 433 nm), as no fluorescence was observed at *λ*_ex_ > 500 nm. No other reagent combinations showed any significant quenching. To summarize, our Stern–Volmer studies indicate that electron and/or energy transfer occurs best between *Co^II^TMPP/PY^+^ and *PY^+^/Co^II^TMPP pairs; a result which means that scenario 1 is not the sole mechanism. The fact that the *Co^II^ lifetime is very short (*τ*_1/2_ ∼ 12 ps)^40^ also makes chemistries that invoke it unlikely. Hence, the interaction between *PY^+^/Co^II^TMPP is the most probable reaction pathway leading to alkyl radical formation. See SI S48–S60 for complete spectroscopic data.

Which way do electrons or energy flow, *i.e.*, from *PY^+^ + Co^II^ → PY˙^++^ + Co^I^ or *PY^+^ + Co^II^ → PY˙ + Co^III^? And, does the resulting intermediate drive carbon-radical formation? To answer this next question, we performed a series of control reactions, systematically removing each component from our reaction mixture. If an electron moves from *PY^+^→Co^II^, the resulting Co^I^ species (scenario 2) should be able to complete our reaction in the absence of NBu_3_, forming the alkyl radical through iodine-atom abstraction or an S_N_2-like mechanism. The same is not true if an electron moves from Co^II^ → *PY^+^, yielding Co^III^. In this case, NBu_3_ would be critically essential, serving as both a sacrificial reductant and possible iodine-atom abstractor. We found that no reaction occurred in the absence of NBu_3_, consistent with *PY^+^ oxidation of Co^II^. Hence, scenario 2 is not viable. We also observed no reaction when PY^+^ was removed, suggesting that Co^II^TMPP does not independently drive productive chemistry, either by electron or energy transfer from its ground or excited states. To further validate the *PY^+^ + Co^II^ → PY˙ + Co^III^ pathway, we determined the excited state redox potentials of *PY^+^ and the ground state redox potentials of Co^II^TMPP. PY^+^ shows irreversible ground state oxidation at *E*_Ox_ = 1.27 V *vs.* SCE and irreversible reduction at *E*_Red_ = −0.79 V *vs.* SCE.^41^ Combined with absorbance and fluorescence measurements, the excited state redox potentials of *PY^+^ are estimated as *E_Red_ = +1.40 V *vs.* SCE and *E_Ox_ = −0.92 V *vs.* SCE. Ground state Co^II^/Co^III^ oxidation occurs at a peak potential of *E*_Ox_ = 1.0 V *vs.* SCE.^42^ Thus, SET oxidation of Co^II^TMPP by *PY^+^ is thermodynamically feasible (∼−10 kcalmol^−1^). See SI S61, S62 and S65 for spectroscopic data. We are now left with scenario 3. Formation of Bu_2_NC·H(CH_2_)_2_CH_3_ abstractor could occur if the newly generated Co^III^TMPP oxidizes NBu_3_ (*E*_Ox_ = 0.97 V *vs.* SCE) to ^+^˙NBu_3_, which is subsequently deprotonated. To test the feasibility of this step, we prepared Co^III^TMPP^+^BF_4_^−^ according to methods by Jones.^43^ For this species, a new absorption peak was observed at 533 nm. Addition of NBu_3_ resulted in quantitative disappearance of this new spectral feature and the reemergence of bands consistent with Co^II^TMPP. We interpreted this as oxidation of NBu_3_ by Co^III^TMPP to yield ^+^˙NBu_3_ and Co^II^. No complex was evident between NBu_3_ and the resulting Co^II^, as later confirmed by separate UV-vis studies; see SI S49. The final step is to show that Bu_2_NC·H(CH_2_)_2_CH_3_ can abstract an iodine atom from our organodiiodide to yield an alkyl radical. Fortunately, this situation already has literature support, being studied by Leonori.^44–46^ Additionally, we performed two control reactions, substituting NBu_3_ for pyridine and DABCO. Neither of these bases can form the requisite α-amino radical needed for halide abstraction. Hence, neither base should work in our reaction. Indeed, this was the case. The resulting α-iodo amine or, more precisely, its iminium congener, is hydrolyzed to dibutylamine and butyraldehyde, with HNBu_2_ being detected by LC-MS of our crude reaction mixture, see SI S79. From the above experiments, we propose the following mechanism shown in Fig. 2D. Upon green light irradiation, *PY^+^ oxidizes Co^II^TMPP to Co^III^TMPP, which oxidizes NBu_3_ to ^+^˙NBu_3_, returning Co^II^. Deprotonation of ^+^˙NBu_3_ (p*K*_a_ ∼8)^47^ yields an α-amino radical that abstracts one of the two iodine atoms from our organodiiodide. Conjugate addition of the resulting carbon-centered radical to ΔSulf^*n*^Pr yields a β-sulfonyl radical, which undergoes β-scission to release ˙Sulf^*n*^Pr and the alkylated dehydroamino acid with the second C–I bond still intact. (Note: the free carbon-radical could also be reversibly captured by Co^II^, stabilizing it for subsequent addition to ΔSulf^*n*^Pr.^48,49^ It does not covalently modify PY^+^ or Co^II^TMPP according to UV-vis tracking of these two reaction components, which appear unaltered at 2 h into the reaction; see SI S49) Reduction of the sulfonyl radical (*E*_Red_ = +0.38 V *vs.* SCE for ˙SO_2_Me)^50^ by reduced PY˙ delivers a sulfinate salt, closing the photoredox cycle. Intramolecular cyclization is then induced in a separate chemical step by adding Cu(acac)_2_ and Zn powder.

## Conclusions

In conclusion, we developed a one-pot, two-step process to transform β-sulfonyldehydroamino acids in peptides into one of several *c*ββs using organodiiodides as ‘ring-transfer reagents’. In the first step, we show that a potent halogen-atom abstractor, borne from the unique combination of an unprecedented photocatalyst, PY^+^, and Co^II^TMPP and green light, attaches the diiodide to the β-sulfonyldehydroamino acid, excising the sulfonyl group and one of the two iodine atoms. Adding Zn/Cu couple then induces ring closure in the same reaction vessel. This two-step process has the advantage of enabling different ring chemotypes to be successfully made and to control their stereochemistry through judicious choice of ligand in step 2. Overall, our procedure provides peptide medicinal chemists with a powerful new synthetic tool and offers key mechanistic insights for designing cooperative metallaphotoredox and halogen-atom transfer reactions in water and at longer wavelengths.

## Author contributions

S. G. and S. B. conceived the project. S. G. carried out the method development and experimental and mechanistic investigations, with assistance from P.-H. C. and N. M. S. B. supervised the project and secured funding. All authors contributed to data analysis and participated in writing and editing the final manuscript.

## Conflicts of interest

The authors declare no competing financial interest.

## Supplementary Material

SC-017-D5SC08845C-s001

## Data Availability

Supplementary information (SI): experimental optimizations, general considerations, experimental procedures, and characterization data for all compounds including NMR spectra and LC-MS spectra. See DOI: https://doi.org/10.1039/d5sc08845c.
