# Supplementary material for: A general route to β,β-carbocyclic sidechains in peptides: an aqueous metallaphotoredox approach driven by green light
Source: Chem Sci. 2026 Feb 5;17(14):7228–35. doi: 10.1039/d5sc08845c (PMC12914560; doi:10.1039/d5sc08845c)
Supplement: SC-017-D5SC08845C-s001 [file SC-017-D5SC08845C-s001.pdf]

Supplementary Information (SI) for Chemical Science.
This journal is © The Royal Society of Chemistry 2026

A General Route to β,β-carbocyclic Sidechains in Peptides: An Aqueous Metallaphotoredox Approach Driven by Green Light

Samuel Gary, Pei-Hsuan Chen, Nin Mai and Steven Bloom*

Table of Contents

A. General Information 3

B. Small Molecule Synthetic Procedures 4

C. Peptide Synthetic Procedures 8

D. Reaction Screening and Optimization 11

a) General Procedure for Photocatalyst Screening 11
b) General Procedure for Radical Precursor Screening 14
c) General Procedure for Electrochemical Approach 14
d) General Procedure for Alkylation of ΔSulf 15
e) General Procedure for the Generation of cββs 15
f) Procedure for Screening of Cobalt Porphyrins and Phthalocyanines 16
g) Reaction performed with different dipeptides 17
h) Procedure for Chiral Ligand Screening 24

E. Peptide Characterization 32

a) Starting Material Characterization 32
b) Product Characterization 34

F. Mechanistic Studies 48

a) Stern-Volmer Quenching Studies 51
b) Cyclic Voltammetry 61

G. Other Mechanistic Studies 64

H. NMR Spectra 67

I. References 80

A. General Information

Unless otherwise stated, reagents and solvents were purchased and used as received (SigmaAldrich, Alfa Aesar, Fisher Scientific, Combi-Blocks, and Oakwood Chemicals). H_2O refers to using Millipore Grade I water. Buffers and solutions were prepared using H_2O and the pH adjusted using a Mettler Toledo FiveEasy pH meter. ^1H NMR and ^{13}C NMR spectra were recorded on a Bruker Ascend 400 NMR spectrometer, 500 MHz spectrometer equipped with a multi-nuclear BBFO cryoprobe, or Bruker AVIII 600 MHz NMR spectrometer equipped with a triple resonance inverse probe. ^1H NMR and ^{13}C NMR spectra were referenced to residual solvent peaks ($CDCl_3$ 7.26 and 77.06 ppm; D_2O 4.79 ppm; CD_3CN 1.94 and 118.26 ppm). Peak multiplicities are designated by the following abbreviations: b, broad; s, singlet; d, doublet; t, triplet; q, quartet; quint, quintet; sept, septet; m, multiplet; dd, doublet of doublets; dt, doublet of triplets; tt, triplet of triplets; and ddd, doublet of doublet of doublets. Electrospray ionization spectra were acquired on a LCT Premier (Waters Corp.) time of flight mass spectrometer (HRMS). LC-MS spectra were acquired on a Waters Acquity UPLC HClass equipped with a Waters QDa Mass Detector using a Discovery BIO Wide Pore C8 HPLC column (Supelco; 5 µm, 10 cm x 4.6 mm). LC spectra were obtained at 214 nm using a gradient of H2O/0.1% formic acid; MeCN/0.1% formic acid. Small molecules were purified via flash chromatography using an Isolera 1 Biotage instrument with the indicated solvent systems with Buchi FlashPure EcoFlex prepacked columns. Peptides were purified via flash chromatography using a Buchi Pure C-815 instrument S3 with Biotage Sfär C18 Duo prepacked columns with the indicated solvent system. Thin-layer chromatography (TLC) was performed on silica gel coated glass TLC plates (Merck, TLC Silica gel 60 F254) and visualized using a UV lamp (254 nm) in combination with various stains. Electrochemistry was performed using an ElectraSyn 2.0 (IKA). Photochemical reactions were carried out using LED lights from Kessil (PR160L, 525 nm, 40 W).

B. Small Molecule Synthetic Procedures

General Procedure for Synthesis of Diols

Dicarboxylic acid or ester (1-5 mmol) was dissolved in THF (0.1 M) in a 100 mL round-bottom flask equipped with a magnetic stir bar. The solution was stirred at room temperature and solid $LiAlH_4$ (5 eqv.) was added in small portions over ~10 minutes. After 5 hours of stirring at room temperature, acetone (5 mL) and sat. aq. potassium sodium tartrate (5 mL) were added slowly to quench the reaction. Stirring continued for an additional 30 minutes, after which the suspension was filtered over Celite. The filtrate was concentrated under reduced pressure, transferred to a separatory funnel, diluted with water (50 mL), and extracted with EtOAc (5 x 50 mL). The combined organic extracts were dried over $MgSO_4$ and concentrated under reduced pressure. The majority of diols were obtained in >90% crude purity and used without additional purification.

General Procedure for Synthesis of Diiodides

The corresponding diol (1-5 mmol) was dissolved in THF (0.1 M) in a 100 mL round-bottom flask. Triphenylphosphine (2.1 eqv.) and imidazole (2.1 eqv.) were added next, and stirring was commenced. In small portions, iodine crystals (2.5 eqv.) were added to the stirring solution. After 8 hours of stirring at room temperature, 10 mL of 1 M aq. sodium ascorbate was added and stirring continued until discoloration of the dark brown solution occurred (5-10 minutes). The reaction mixture was concentrated under reduced pressure, transferred to a separatory funnel, diluted with DCM (100 mL) and water (100 mL). The organic fraction was removed, and the aqueous layer extracted once more with DCM (100 mL). The combined organic fractions were dried over $MgSO_4$, concentrated under reduced pressure, and purified by flash chromatography (silica gel; hexanes/EtOAc). The pure diiodides were stored in amber vials and were re-purified by passing over a silica plug with hexanes prior to further use.

The majority of small molecules (diiodides forming products **3a**-**3f** and radical precursors **S1-S4**) were all synthesized and characterized in our prior report.[1]

trans-1,2-bis(iodomethyl)cyclopropane

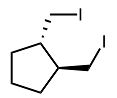

Synthesized in two steps from *trans*-1,2-cyclopentanedicarboxylic acid on 5 mmol scale.

Yield: 221 mg of a pale oil (13% over two steps)

^{1}H NMR (400 MHz, $CDCl_3$): d 3.36 (2H, dd, *J* = 9.8, 4.4 Hz), 3.19 (2H, dd, *J* = 9.8, 6.8 Hz), 2.02-1.88 (2H, m), 1.82-1.70 (2H, m), 1.66 (2H, td, *J* = 7.5, 6.0 Hz), 1.49 (2H, ddd, *J* = 12.7, 8.0, 6.3 Hz).

Spectral data are consistent with literature.[2]

Ac-ΔAlaBr-OMe

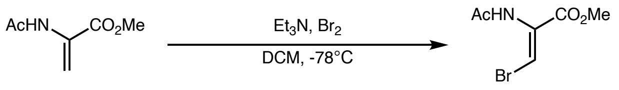

Ac-Dha-OMe (5.0 g, 35.0 mmol) was dissolved in CH_2Cl_2 (80 mL) and cooled to -78 °C. Next, Br_2 (1.97 mL, 38.4 mmol) was added slowly with rapid stirring. After 15 minutes of stirring at -78 °C, triethylamine (5.35 mL, 38.4 mmol) was added dropwise. The reaction mixture was stirred at the same temperature for 3 hours, then allowed to warm to room temperature and transferred to a separatory funnel. 100 mL of 0.1 M aq. sodium ascorbate solution was added, the biphasic mixture shaken vigorously, and the organic layer removed. The remaining aqueous layer was extracted again with CH_2Cl_2 (2 x 50 mL) and the combined organic fractions were dried over $MgSO_4$, filtered, and concentrated under reduced pressure. Purification by flash chromatography (silica gel; hexanes/ethyl acetate) yielded Ac-ΔAlaBr-OMe as a pale orange solid (4.22 g, 54% yield).

^1H NMR (400 MHz, $CDCl_3$): d 7.14 (1H, s), 6.92 (1H, br. s), 3.84 (3H, s), 2.17 (3H, s).

Ac-ΔCysCy-OMe

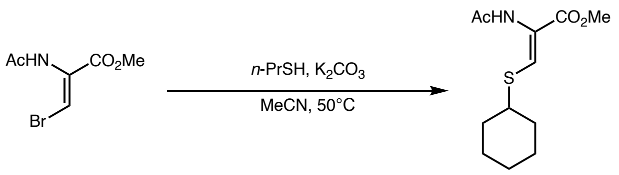

1.0 g of Ac-ΔAlaBr-OMe (4.50 mmol), K_2CO_3 (1.87 g, 13.5 mmol), and cyclohexanethiol (1.65 mL, 13.5 mmol) were dissolved in 70 mL of MeCN. The reaction mixture was heated to 50 °C under a reflux condenser for 6 hours. The reaction mixture was concentrated to dryness under reduced pressure, redissolved in ethyl acetate (50 mL), and diluted with water (100 mL). The organic layer was removed, and the aqueous layer was extracted again with ethyl acetate (2 x 50 mL). The organic fractions were combined, dried over $MgSO_4$, filtered, and concentrated. The resulting residue was then purified by flash chromatography (silica gel; hexanes/ethyl acetate) to afford 895 mg of Ac-ΔCysCy-OMe as a white powder (77% yield).

^1H NMR (400 MHz, $CDCl_3$): d 7.47 (1H, s), 7,43 (1H, br. s.), 3.62 (3H, s), 2.87 (1H, td, J = 8.7, 5.1 Hz), 1.99 (3H, s), 1.95-1.86 (2H, m), 1.66 (2H, dt, J = 7.8, 4.1 Hz), 1.51 (1H, dt, J = 12.7, 3.9 Hz), 1.35-1.16 (4H, m).

Ac-ΔSulfCy-OMe

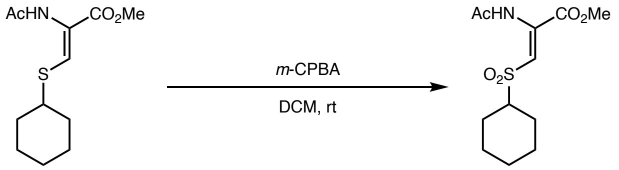

895 mg of Ac-ΔCysCy-OMe (3.48 mmol) was dissolved in DCM (35 mL) in a 100 mL round-bottom flask equipped with a magnetic stir bar. *m*-CPBA (8.70 mmol, 2.5 equivalents, 1.50 g) was added in small portions over ~15 minutes with rapid stirring. After two hours, the reaction mixture was filtered and diluted with additional DCM (30 mL) and sat. aq. $NaHCO_3$ (50 mL). The organic layer was removed, and the aqueous layer was extracted twice more with DCM (50 mL). The combined DCM layers were dried over $MgSO_4$, filtered, and concentrated under reduced pressure. The resulting residue was then purified by flash chromatography (silica gel; DCM/9:1 DCM:MeOH) to yield Ac-ΔSulfCy-OMe (656 mg, 65%) as a white powder.

^{1}H NMR (400 MHz, $CDCl_3$): d 9.59 (1H, s), 5.65 (1H, s), 3.85 (3H, s), 2.87 (1H, tt, *J* = 12.2, 3.5 Hz), 2.24 – 2.10 (5H, m), 1.93 (2H, dq, *J* = 13.4, 3.2 Hz), 1.73 (1H, dd, *J* = 9.6, 6.2 Hz), 1.47 (2H, qd, *J* = 12.4, 3.4 Hz), 1.38 – 1.13 (3H, m).

C. Peptide Synthetic Procedures

General Procedure for Peptide Synthesis

Solid-phase peptide synthesis was performed with Rink Amide MBHA Resin using a 250-mL glass peptide synthesis vessel with N_2 agitation. The resin was initially swelled with CH_2Cl_2 (10 mL/g resin) for 1 hour. Fmoc deprotection was carried out using 4:1 DMF:piperidine (10 mL/g resin) for 30 minutes. Amide couplings were performed using 3 equivalents of Fmoc amino acid, 3 equivalents of HOBT, 3 equivalents of HBTU, and 3 equivalents of DIPEA for 2 hours at a concentration of 0.5 M. In between coupling and deprotection steps, the resin was washed sequentially with DMF, MeOH, and CH_2Cl_2 (10 mL/g resin each). Acetylation was performed using a solution of DMF:pyridine:Ac_2O (2:1:1, 10 mL/g resin) for 2 hours. Global deprotection and resin cleavage was accomplished with 95:2.5:2.5 TFA:H_2O:TIPS (10 mL/g resin) for 2 hours. The cleavage solution was concentrated under reduced pressure to ~15% volume and diluted with Et_2O in an ice bath until precipitation occurred. The solid was collected via vacuum filtration, redissolved in aqueous methanol, and purified by reverse-phase flash chromatography (C18; H_2O/0.01% formic acid:MeCN/0.01% formic acid). The purified peptide was lyophilized to yield a white powder.

Conversion of Ac-GPCF-NH_2 to Ac-GP(Dha)F-NH_2

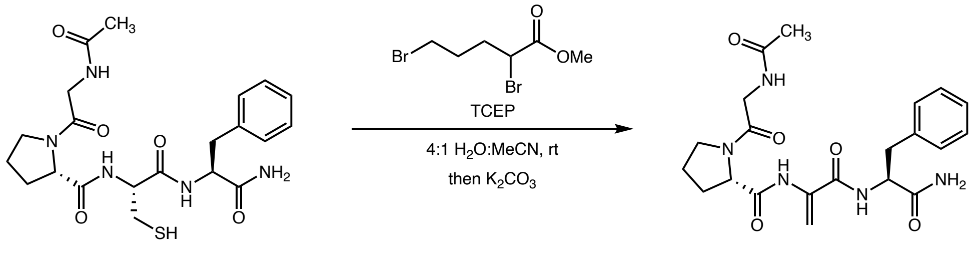

To a 100 mL round-bottom flask was added Ac-GPCF-NH_2 (200 mg, 0.43 mmol), 4:1 water: acetonitrile (50 mL, 8.6 mM) and tris(2-carboxyethyl)phosphine hydrochloride (19 mg, 0.065 mmol). The resulting solution was stirred at ambient temperature for 1 hour, followed by the addition of methyl 2,5-dibromovalerate (80 mL, 0.51 mmol) and rapid stirring for 3 hours. K_2CO_3

(238 mg, 1.72 mmol) was finally added. After 2 hours of stirring at room temperature, the reaction was directly concentrated under reduced pressure and purified by reverse-phase flash chromatography (C18; H_2O/0.01% formic acid:MeCN/0.01% formic acid). After concentration of the purified peptide under reduced pressure, lyophilization yielded 158 mg (85% yield) of Ac-GP(Dha)F-NH_2 as a fluffy white powder.

Conversion of Ac-GP(Dha)F-NH_2 to Ac-GP(ΔAlaBr)F-NH_2

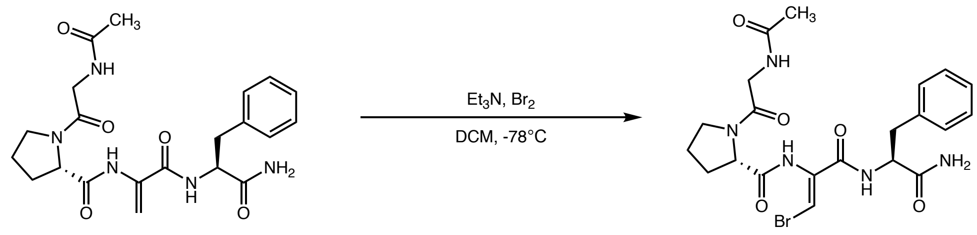

Ac-GP(Dha)F-NH_2 (158 mg, 0.37 mmol) and dichloromethane (25 mL, 15 mM) were added to a 50 mL round-bottom flask equipped with a magnetic stir bar. The cloudy suspension was cooled to -78 °C, followed by the dropwise addition of bromine (57 mL, 1.1 mmol) and then triethylamine (155 mL, 1.1 mmol). After two hours of stirring at -78 °C, the reaction was allowed to warm to room temperature and stirred at ambient temperature for one additional hour. Concentration of the pale-yellow solution under reduced pressure followed by reverse-phase flash chromatography (C18; H_2O/0.01% formic acid:MeCN/0.01% formic acid) and lyophilization gave 127 mg (68% yield) of Ac-GP(ΔAlaBr)F-NH_2 as a fluffy white powder.

Conversion of Ac-GP(ΔAlaBr)F-NH$_2$ to Ac-GP(ΔCys^{n}Pr)F-NH$_2$

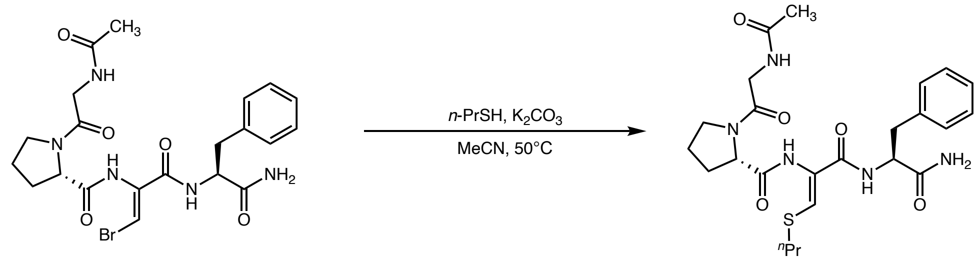

Ac-GP(ΔAlaBr)F-NH$_2$ (127 mg, 0.25 mmol) was dissolved in acetonitrile (25 mL, 10 mM) in a 50 mL round-bottom flask. 1-propanethiol (116 mL, 1.25 mmol) and K_2CO_3 (173 mg, 1.25 mmol) were then added, and the resulting suspension was heated at 50 °C for three hours. Upon cooling to room temperature, the reaction was filtered to remove KBr, concentrated under reduced pressure, and purified by reverse-phase flash chromatography (C18; H_2O/0.01% formic acid:MeCN/0.01% formic acid). Lyophilization of the purified product provided 86 mg (68% yield) of Ac-GP(ΔCys^{n}Pr)F-NH$_2$ as a fluffy white powder.

Additional vinyl thioethers were prepared in an analogous manner using 5-10 mg of Ac-GP(ΔAlaBr)F-NH$_2$.

Conversion of Ac-GP(ΔCys^{n}Pr)F-NH$_2$ to Ac-GP(ΔSulf^{n}Pr)F-NH$_2$

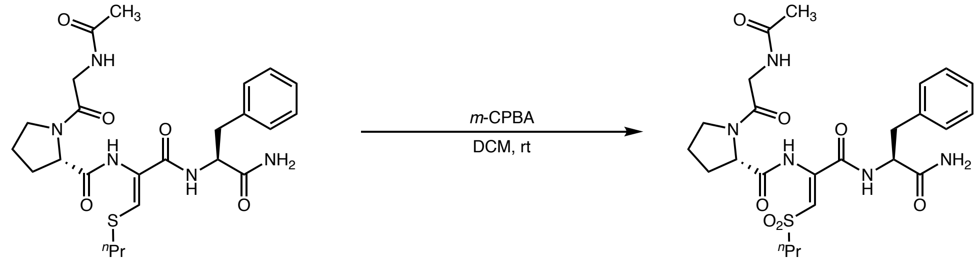

Ac-GP(ΔCys^{n}Pr)F-NH$_2$ (86 mg, 0.17 mmol) was dissolved in dichloromethane (12 mL, 14 mM) in a 50 mL round-bottom flask equipped with a magnetic stir bar. 3-chloroperbenzoic acid (88 mg, 0.51 mmol) was added in one portion and stirring was continued at room temperature for

two hours. The solvent was evaporated under reduced pressure, followed by purification by (C18; H_2O/0.01% formic acid:MeCN/0.01% formic acid). The purified peptide was lyophilized to yield 78 mg (85% yield) of Ac-GP(ΔSulf^{n}Pr)F-NH_2 as a fluffy white powder.

Additional vinyl sulfones were prepared in an analogous manner using 5-10 mg of Ac-GP(ΔAlaBr)F-NH_2.

D. Reaction Screening and Optimization

a) General Procedure for Photocatalyst Screening

For photochemical reactions, screening was conducted on 0.5 mg scale of Ac-GP(ΔSulfCb)F-NH_2. Reactions were prepared using stock solutions of reagents and catalysts and degassed with N_2 prior to irradiation with two Kessil LEDs (525 nm or 440 nm) for 6 hours. Phtotocatalysts used in screening are commercially available and shown in **Table S1** and **Table S2.** All reactions were conducted with 10 mol% photocatalyst, 5 equivalents of DIPEA, and 5 equivalents of diiodobutane in 1:1 H_2O:TFE (2 mM). At the completion of the reaction, reactions were diluted with 1:1 MeOH:MeCN to 1 mM, filtered, and analyzed by LC-MS.

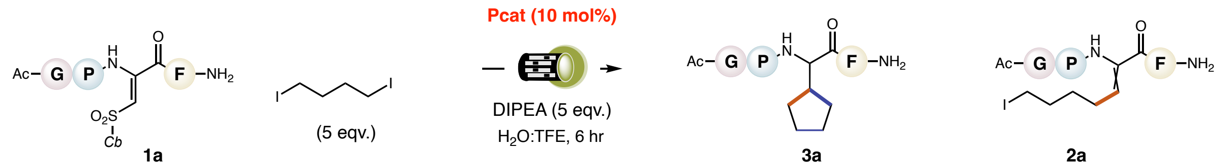

Table S1. Survey of photocatalysts used under green light irradiation (525 nm) in this study.

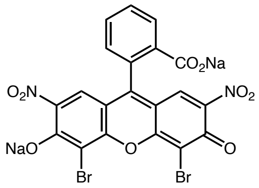

eosin B

3a: 0%; **2a**: 0%

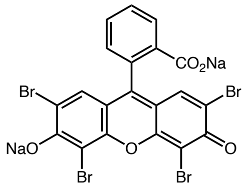

eosin Y

3a: 0%; **2a**: 0%

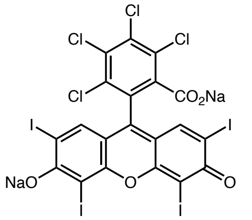

rose bengal

3a: 0%; **2a**: 0%

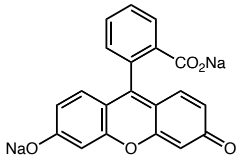

fluorescein

3a: 0%; **2a**: 0 %

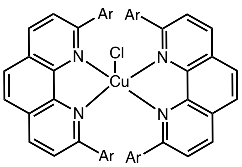

Cu(dap)$_2$Cl

3a: 0%; **2a**: 0%

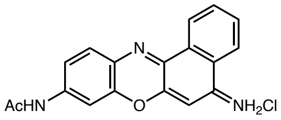

Darrow Red

3a: 0%; **2a**: 0%

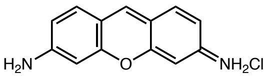

Pyronin Y

3a: 0%; **2a**: ***15%***

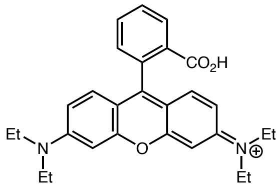

rhodamine B

3a: 0%; **2a**: 0%

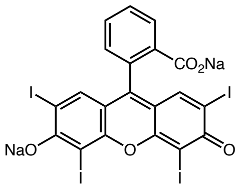

erythrosin

3a: 0%; **2a**: 0%

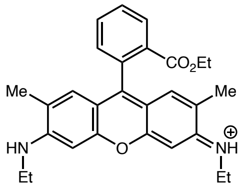

rhodamine 6G

3a: 0%; **2a**: 0%

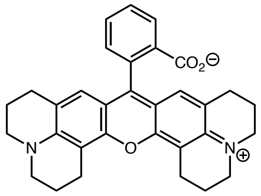

rhodamine 101

3a: 0%; **2a**: 0%

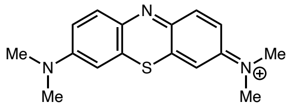

Methylene Blue

3a: 0%; **2a**: 0%

Table S2. Survey of photocatalysts used under blue light irradiation (440 nm) in this study.

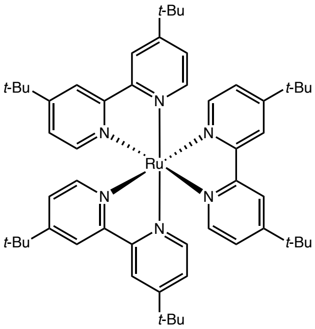

$Ru(dtbpy)_3(PF_6)_2$

3a: 0%; **2a**: 0%

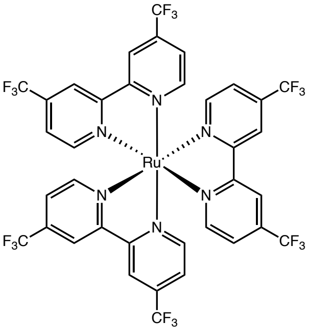

Ru(*p*-CF_3bpy)$_3$(BF_4)$_2$

3a: 0%; **2a**: 0%

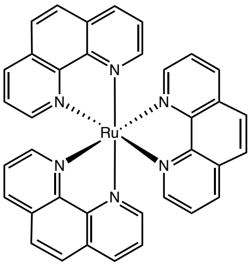

$Ru(phen)_3Cl_2$

3a: 0%; **2a**: 0%

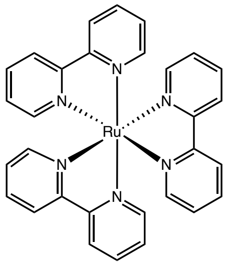

$Ru(bpy)_3Cl_2$

3a: 0%; **2a**: 0%

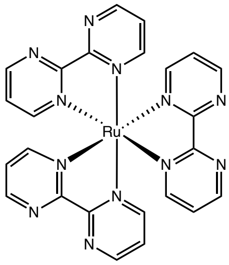

$Ru(bpm)_3Cl_2$

3a: 0%; **2a**: 0%

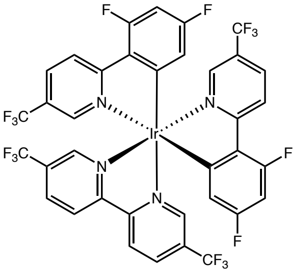

Ir(dFCF$_3$ppy)$_2$(5,5'-dCF$_3$bpy)PF$_6$

3a: 0 %; **2a**: 0%

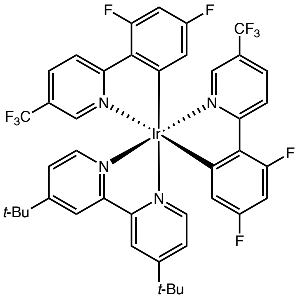

Ir(dFCF$_3$ppy)$_2$(dtbpy)PF$_6$

3a: 0%; **2a**: 0%

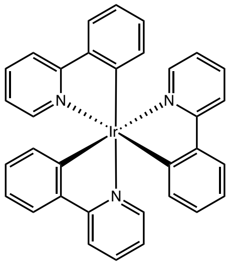

$Ir(ppy)_3$

3a: 0%; **2a**: 0%

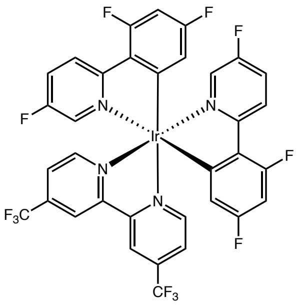

Ir(dFFppy)$_2$(4,4'-dCF$_3$bpy)PF$_6$

3a: 0%; **2a**: 0%

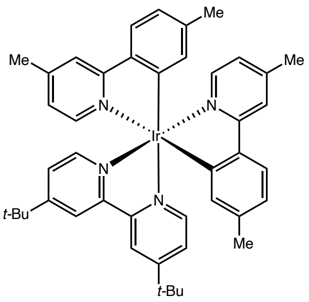

Ir(Me[Me]ppy)$_2$(dtbpy)PF$_6$

3a: 0%; **2a**: 0%

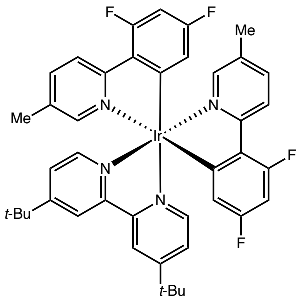

Ir(dF[Me]ppy)$_2$(dtbpy)PF$_6$

3a: 0%; **2a**: 0%

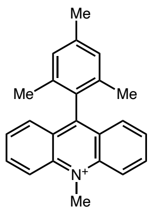

9-mesityl-10-methyl acridinium

3a: 0%; **2a**: 0%

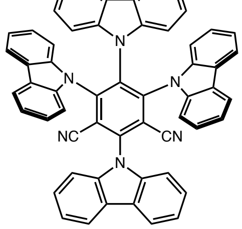

4CzIPN

3a: 0%; **2a**: 0%

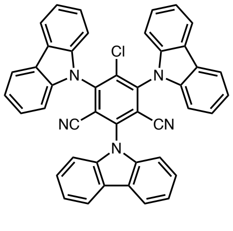

3CzClIPN

3a: 0%; **2a**: 0%

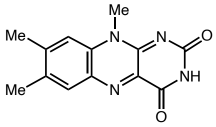

Lumiflavin

3a: 0%; **2a**: 0%

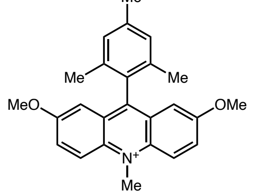

2,7-dOMe-9-mesityl-
10-methyl acridinium

3a: 0%; **2a**: 0%

b) General Procedure for Radical Precursor Screening

Radical precursor screening was conducted on 0.5 mg scale of Ac-GP(ΔSulfCb)F-NH_2. Reactions were prepared using stock solutions of reagents and catalysts and degassed with N_2 prior to irradiation with two Kessil LEDs (525 nm) for 6 hours. All reactions were conducted with 10 mol% Pyronin Y, 5 equivalents of DIPEA, and 5 equivalents of radical precursor, shown in **Table S3**, in 1:1 H_2O:TFE (2 mM). At the completion of the reaction, reactions were diluted with 1:1 MeOH:MeCN to 1 mM, filtered, and analyzed by LC-MS.

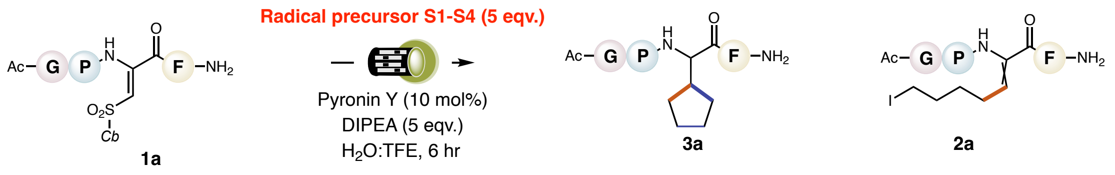

Table S3. Structures of radical precursors used in screening for this study.

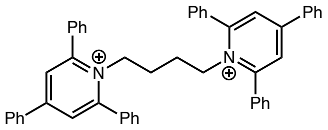

S1

3a: 0%; **2a**: 0%

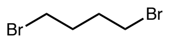

S2

3a: 0%; **2a**: trace

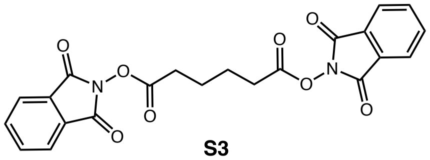

S3

3a: 0%; **2a**: 0%

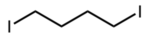

S4

3a: 0%; **2a**: 15%

c) General Procedure for Electrochemical Approach

For electrochemical approaches, a scale of 5.0 mg of Ac-GP(ΔSulfCb)F-NH_2 was used. All electrochemical reactions were conducted using an IKA ElectraSyn 2.0. Electrochemical reactions were performed using constant voltage in an undivided cell ranging from +0.5 V to +2.0V (using 5 equivalents of DIPEA as electron donor and/or HAT reagent) and -0.5 V to -2.0 V vs. Ag/AgCl (using sacrificial anode) with combinations of graphite, nickel, magnesium, and zinc

electrodes. Electrochemical reactions were performed using 1 mM of 1:1 H_2O:TFE and run for a total of 5 F/mol, after which point the reaction mixture was directly filtered and analyzed by LC-MS. None of these conditions afforded **2a** or **3a**.

d) General Procedure for Alkylation of ΔSulf

To a 2-dram vial equipped with a magnetic stir bar, Ac-GP(ΔSulf^{n}Pr)F-NH_2 (5.0 mg, 9.34 μmol) was dissolved in MeCN (1168 μL), H_2O (933 μL), and 0.1 M phosphate buffer (234 μL) to a final concentration of 4 mM. Next, pyronin Y (117 μL of a 5.56 mM aqueous solution), CoTMPP (11.1 μL from a 5 mg/mL DMF stock solution), and NBu_3 (11.1 μL, 46.7 μmol) were added. Finally, the corresponding alkyl diiodide was added (10 eqv., 93.4 μmol). The resulting solution was degassed with N_2 for 5 minutes, sealed with parafilm, and irradiated with two Kessil LEDs (525 nm) for 6 hours. After the reaction was complete, saturated aqueous ammonium chloride (2 mL) was added, followed by ethyl acetate (2 mL). The organic fraction was removed, and the aqueous layer was extracted three additional times with ethyl acetate (2 mL each). The combined organic fractions were concentrated and redissolved in 4 mL of 1:1 MeCN:MeOH and analyzed by LC-MS.

e) General Procedure for the Generation of *c*ββs

The peptide mixture from the prior step was concentrated under reduced pressure. At this point, $Cu(acac)_2$ (10 eqv., 24.4 mg), zinc powder (10 eqv., 6.1 mg), H_2O (2.24 mL), sat. aq. NH_4Cl (0.1 mL) and TFE (2.34 mL) were added. The suspension was degassed with N_2 for 5 minutes, sonicated, and rapidly stirred for 4 hours. After the reaction was complete, saturated aqueous ammonium chloride (2 mL) was added, followed by ethyl acetate (2 mL). The organic fraction was removed, and the aqueous layer was extracted three additional times with ethyl acetate (2 mL each). The combined organic fractions were concentrated and redissolved in 4 mL of 1:1 MeCN:MeOH and analyzed by LC-MS. The product was then purified by flash chromatography (C18; H_2O/0.01% HCO_2H; MeCN/0.01% HCO_2H).

f) Procedure for Screening of Cobalt Porphyrins and Phthalocyanines

All porphyrins and phthalocyanines used in this study (**Table S4**) are commercially available. *Apo*-ligands were metalated using $CoCl_2$ with standard literature procedures.[3]

To a 1-dram vial equipped with a magnetic stir bar, Ac-GP(ΔSulf^{n}Pr)F-NH_2 (0.3 mg, 0.56 μmol) was dissolved in MeCN (140 μL) and H_2O (140 μL) to a final concentration of 2 mM. Next, pyronin Y (7.0 μL of a 5.56 mM aqueous solution), CoTMPP (2.68 μL from a 6.3 mM DMF stock solution), and NBu_3 (0.67 μL, 2.8 μmol) were added. Finally, 1,4-diiodobutane was added (10 eqv., 5.6 μmol). The resulting solution was degassed with N_2 for 5 minutes, sealed with parafilm, and irradiated with two Kessil LEDs (525 nm) for 6 hours. After the reaction was complete, the solution was filtered and analyzed by LC-MS.

Table S4. Screening of additional Co(II) porphyrin and phthalocyanine ligands.

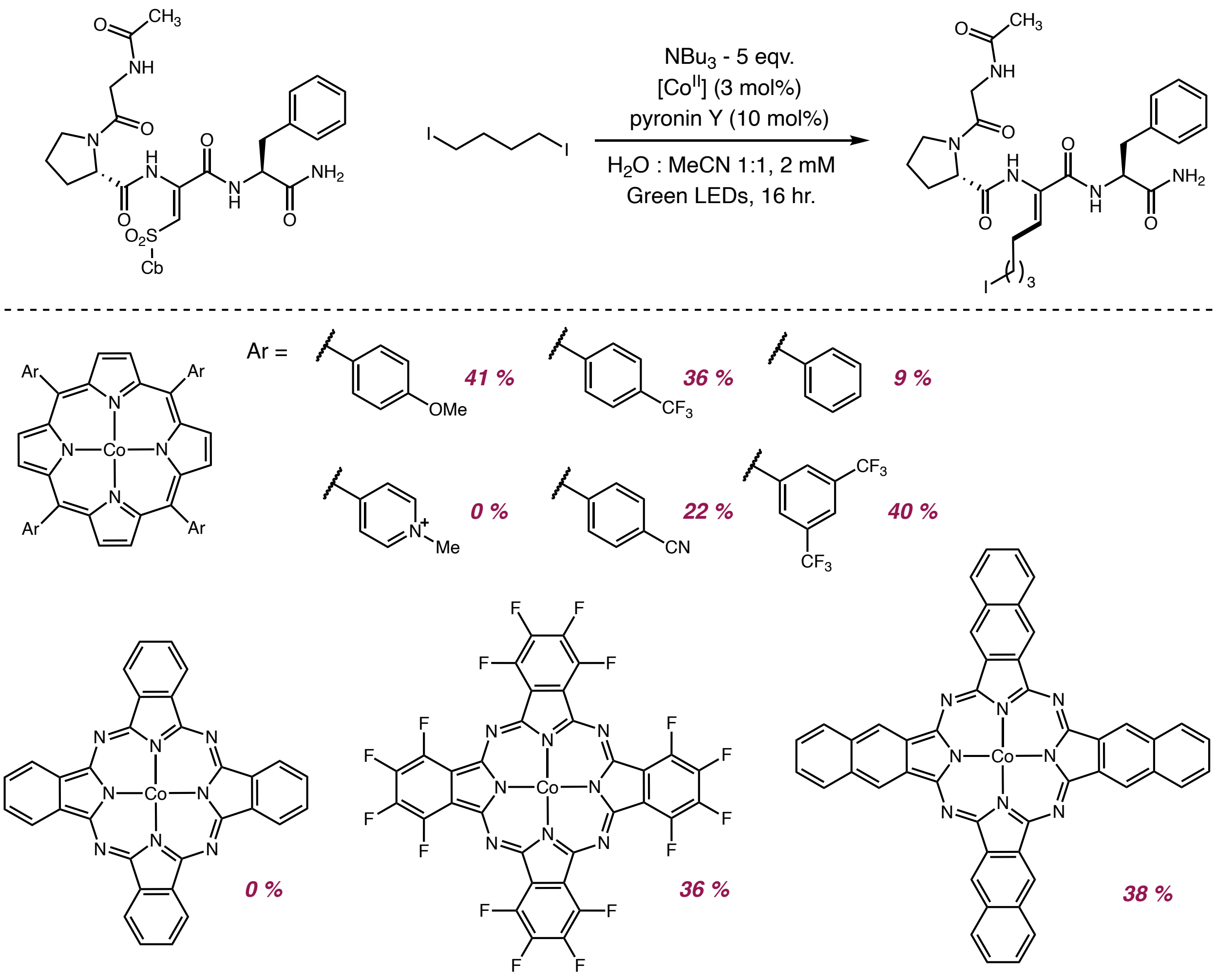

g) Reaction performed with different dipeptides

Table S5. Reaction conversion using different ΔSulfCy containing dipeptides

**Dipeptide** (AcNH-ΔSulfCy-***AA***-CO_2Me) **AA =**	**Conversion**	
	**Alkylation (Step 1)**	**Cyclization (Step 2)**
Leu	66	47
Trp	27	37
Phe	65	23
Val	42	25
Tyr	21	33
His	26	12
Lys	0	-
Met	0	-

Reactions with dipeptides were conducted following the general procedures outlined on Page **S14**. The crude LC-MS data for the reactions are shown below.

AcNH-ΔCySulf-*Leu*-OMe

Step1

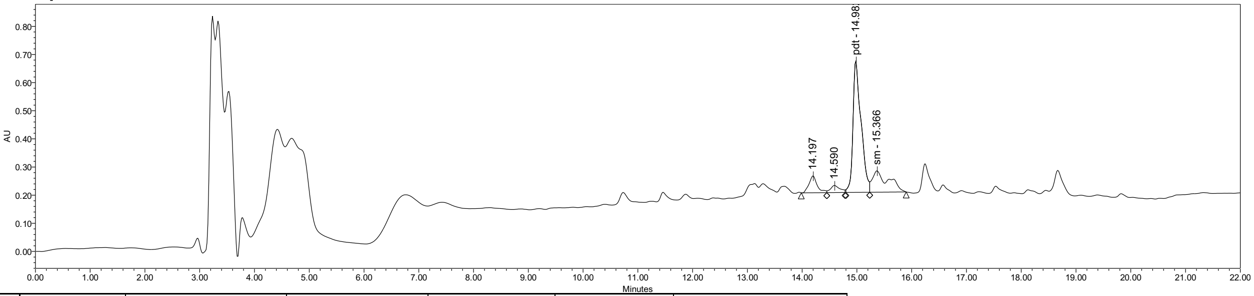

	Name	Retention Time	Area	% Area	Base Peak	Base Peak (Combined)
1		14.197	623594	8.59	146.19	146.19
2		14.590	302167	4.16	440.63	440.59
3	pdt	14.982	4781196	65.86	146.18	146.19
4	sm	15.366	1552578	21.39	158.26	158.25

Step2

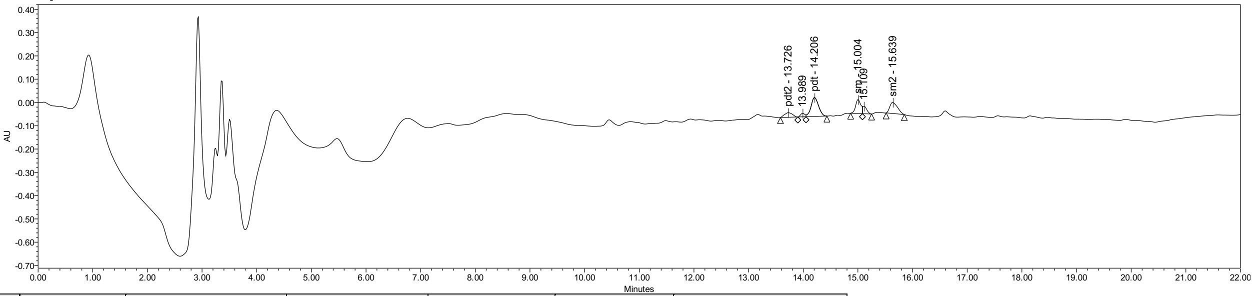

	Name	Retention Time	Area	% Area	Base Peak	Base Peak (Combined)
1	pdt2	13.726	198519	9.49	335.28	335.30
2		13.989	82631	3.95	569.49	569.52
3	pdt	14.206	777704	37.19	335.33	335.32
4	sm	15.004	397358	19.00	439.35	439.32
5		15.109	168198	8.04	315.41	337.30
6	sm2	15.639	466633	22.32	354.43	368.27

AcNH-ΔCySulf-*Trp*-OMe

Step1

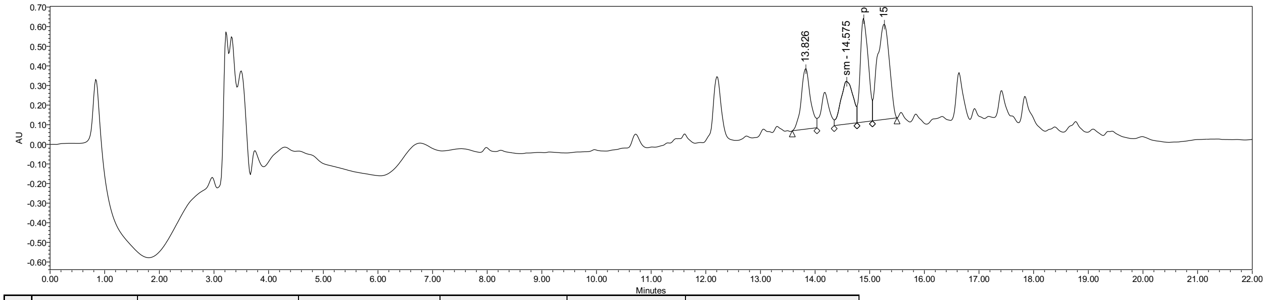

	Name	Retention Time	Area	% Area	Base Peak	Base Peak (Combined)
1		13.826	3602394	18.22	219.26	219.26
2	sm	14.575	3395776	17.17	513.49	513.56
3	pdt	14.886	5336158	26.98	512.32	200.42
4		15.263	7442448	37.63	158.22	158.25

Step2

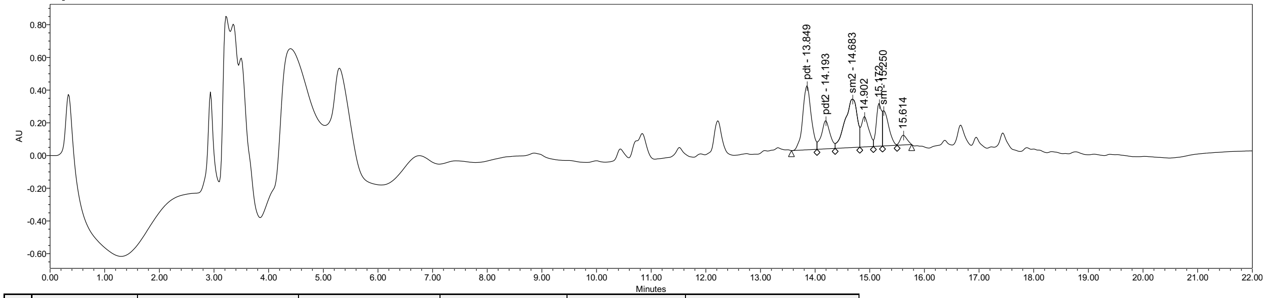

	Name	Retention Time	Area	% Area	Base Peak	Base Peak (Combined)
1	pdt	13.849	4221076	25.62	386.44	386.40
2	pdt2	14.193	1884277	11.44	386.41	386.37
3	sm2	14.683	4590626	27.86	410.48	513.57
4		14.902	1840036	11.17	512.28	512.28
5		15.172	1785112	10.83	349.30	349.29
6	sm	15.250	1653317	10.03	349.30	514.28
7		15.614	503570	3.06	553.29	368.38

AcNH-ΔCySulf-*Phe*-OMe

Step1

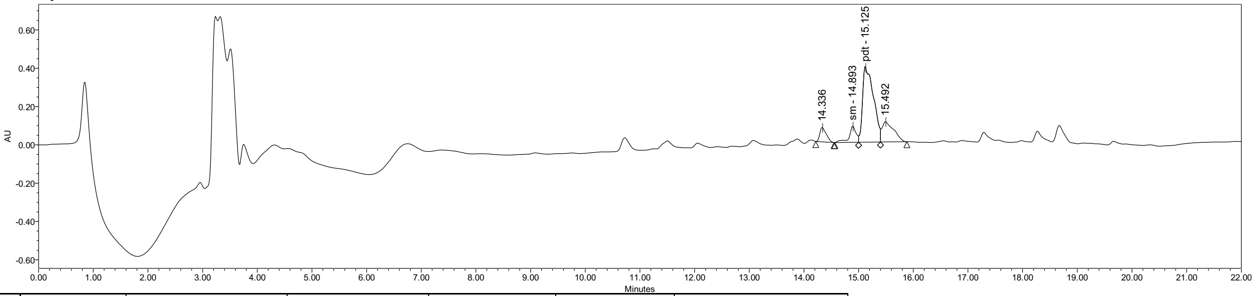

	Name	Retention Time	Area	% Area	Base Peak	Base Peak (Combined)
1		14.336	614141	7.32	403.36	403.36
2	sm	14.893	745044	8.88	459.42	474.61
3	pdt	15.125	5458355	65.02	495.26	495.28
4		15.492	1576888	18.78	368.34	368.34

Step2

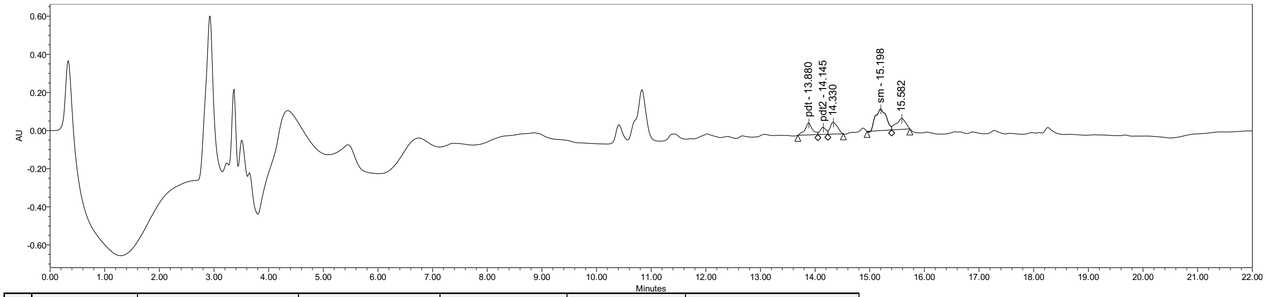

	Name	Retention Time	Area	% Area	Base Peak	Base Peak (Combined)
1	pdt	13.880	582456	15.85	369.33	395.37
2	pdt2	14.145	276395	7.52	369.33	369.36
3		14.330	576284	15.68	403.33	403.32
4	sm	15.198	1562126	42.51	459.36	495.26
5		15.582	677381	18.43	368.43	336.24

AcNH-ΔCySulf-*Val*-OMe

Step1

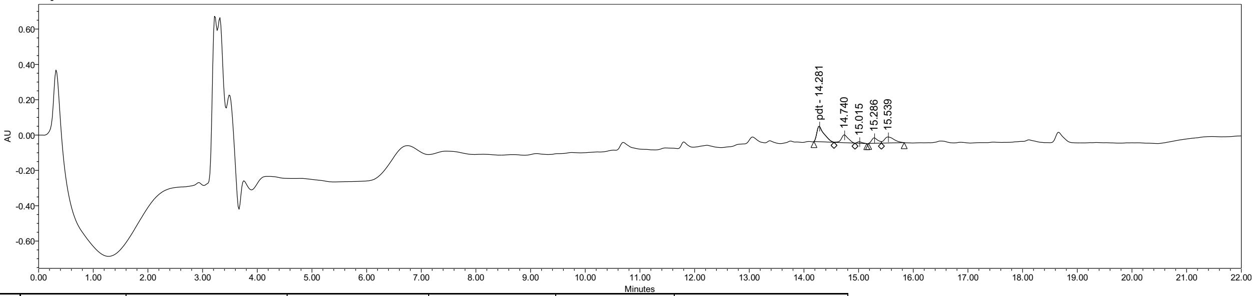

	Name	Retention Time	Area	% Area	Base Peak	Base Peak (Combined)
1	pdt	14.281	803520	42.21	425.33	329.30
2		14.740	385553	20.26	427.33	427.36
3		15.015	54078	2.84	427.35	427.36
4		15.286	239468	12.58	875.21	368.37
5		15.539	420837	22.11	368.39	368.37

Step2

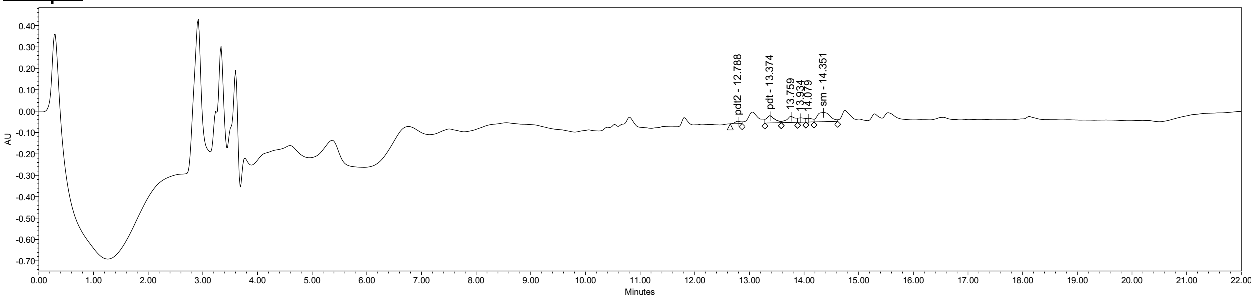

	Name	Retention Time	Area	% Area	Base Peak	Base Peak (Combined)
1	pdt2	12.788	82898	4.67	321.33	321.31
2	pdt	13.374	347963	19.60	355.34	321.28
3		13.759	319425	17.99	357.38	258.34
4		13.934	171984	9.69	258.32	258.33
5		14.079	142043	8.00	335.16	312.19
6	sm	14.351	710811	40.04	301.36	329.34

AcNH-ΔCySulf-*Tyr*-OMe

Step1

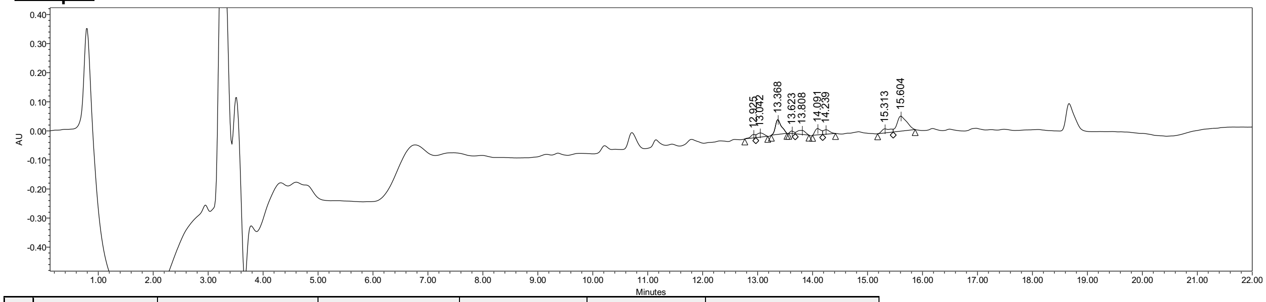

	Name	Retention Time	Area	% Area	Base Peak	Base Peak (Combined)
2	sm	12.925	70014	3.83	453.43	453.41
3		13.042	125635	6.87	271.75	271.61
4	463 pdt	13.368	383267	20.96	511.23	511.26

Step2

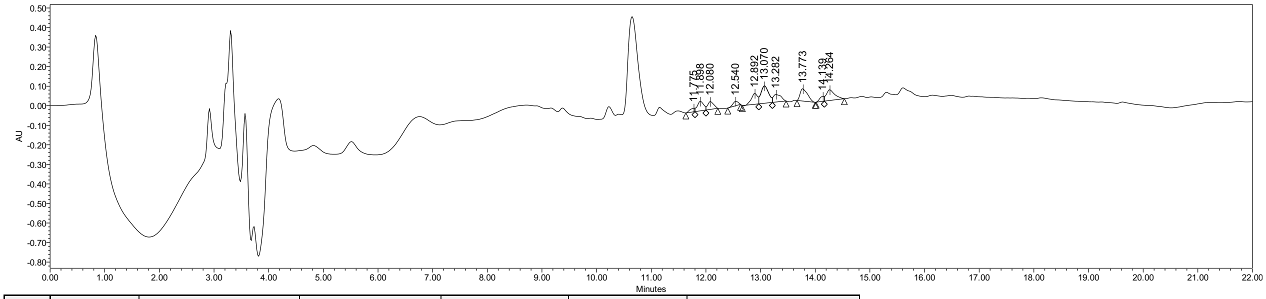

	Name	Retention Time	Area	% Area	Base Peak	Base Peak (Combined)
1	1	10.650				
2		11.775	117975	2.97	411.34	411.37
3		11.898	390967	9.85	385.33	363.36
4		12.080	306053	7.71	363.40	363.33
5		12.540	151144	3.81	322.26	322.28
6	pdt	12.892	446966	11.26	365.47	510.45
7	pdt2	13.070	864159	21.76	365.43	365.39
8	sm	13.282	376736	9.49	475.37	270.53
9		13.773	588757	14.83	359.40	359.38
10		14.139	141058	3.55	359.20	284.35
11		14.264	586614	14.77	587.49	509.42

AcNH-ΔCySulf-*His*-OMe

Step1

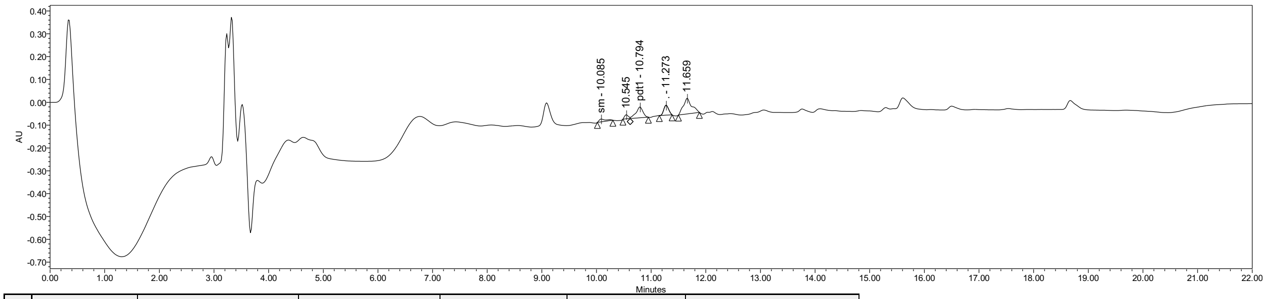

	Name	Retention Time	Area	% Area	Base Peak	Base Peak (Combined)
1	sm	10.085	101883	6.36	408.40	408.45
2		10.545	88041	5.50	449.40	428.45
3	pdt1	10.794	418661	26.13	463.32	463.33
4	.	11.273	268692	16.77	498.39	498.42
5		11.659	724842	45.24	534.31	534.34

Step2

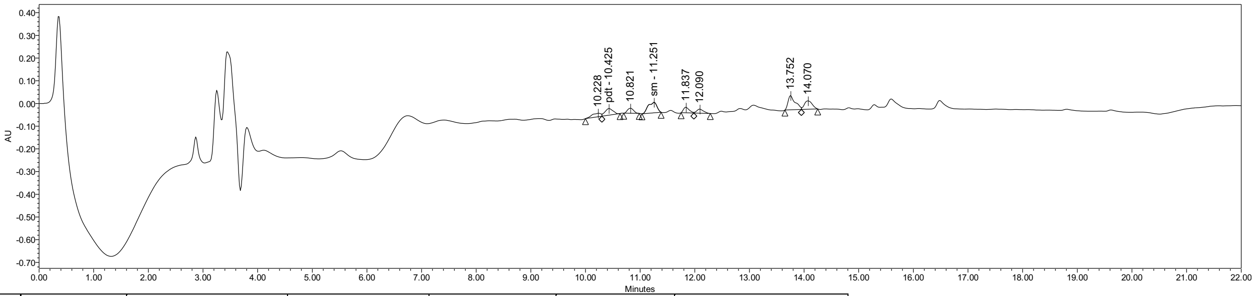

	Name	Retention Time	Area	% Area	Base Peak	Base Peak (Combined)
1		10.228	182011	7.48	408.45	408.46
2	pdt	10.425	295507	12.14	339.37	339.33
3		10.821	169879	6.98	449.50	449.49
4	sm	11.251	518194	21.29	410.48	410.49
5		11.837	178319	7.33	338.53	338.40
6		12.090	151874	6.24	407.56	407.66
7		13.752	570253	23.43	359.26	466.56
8		14.070	368161	15.12	258.32	258.27

h) Procedure for Chiral Ligand Screening

Chiral ligands (84.9 μmol) were weighed out into individual 1-mL vials equipped with stir bars. TFE (213 μL) was next added, followed by $Cu(acac)_2$ (84.9 μmol, 22 mg) and the solutions were degassed with N_2 for 3 minutes and then heated at 70 °C for 24 hours. The solutions were then cooled to room temperature and used directly in the next step.

To a 2-dram vial equipped with a magnetic stir bar, Ac-GP(ΔSulf^{n}Pr)F-NH_2 (5.0 mg, 9.34 μmol) was dissolved in MeCN (1168 μL), H_2O (933 μL), and 0.1 M phosphate buffer (234 μL) to a final concentration of 4 mM. Next, Pyronin Y (117 μL of a 5.56 mM aqueous solution) and CoTMPP (11.1 μL from a 5 mg/mL DMF stock solution) and NBu_3 (11.1 μL, 46.7 μmol) were added. Finally, the corresponding alkyl diiodide was added (10 eqv., 93.4 μmol). The resulting solution was degassed with N_2 for 5 minutes, sealed with parafilm, and irradiated with two Kessil LEDs (525 nm) for 6 hours. After the reaction was complete, saturated aqueous ammonium chloride (2 mL) was added, followed by ethyl acetate (2 mL). The organic fraction was removed, and the aqueous layer was extracted three additional times with ethyl acetate (2 mL each). The combined organic fractions were concentrated, redissolved in 2.24 mL of H_2O (sonication was required, forming a cloudy solution), and distributed into the vials containing the chiral ligands. Zinc powder (6 mg) was then added followed by sat. aq. NH_4Cl (10 μL). The reactions were then degassed with N_2 (5 min.), sealed with parafilm, and stirred rapidly at room temperature for 6 hours. Upon reaction completion, the reactions were filtered, washed with 0.2 mL of 1:1 MeCN:MeOH, and analyzed by LC-MS. Product diastereoselectivity was determined by the ratio of ESI+ intensity of the two product peaks rather than UV-Vis, which was complicated by the excess of chiral ligands used.

No Ligand Added

65:35 L:D

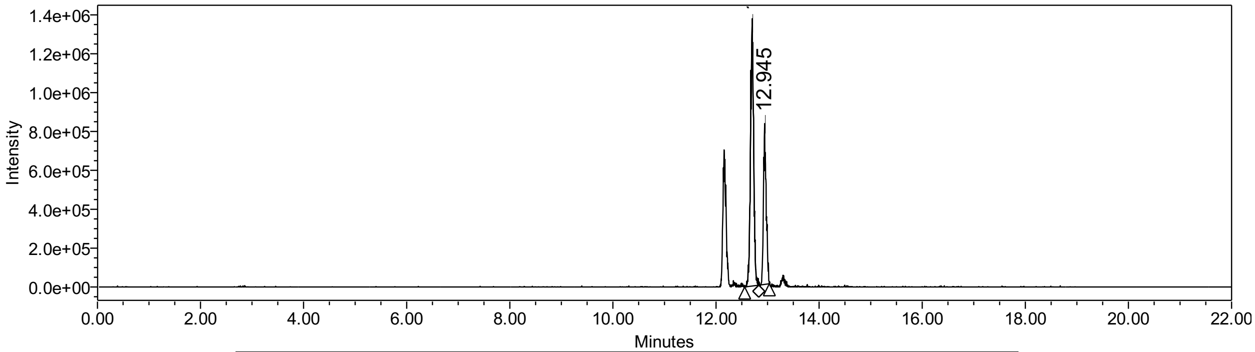

	Name	Retention Time	Area	% Area
1	(L)-**3b**	12.701	5645763	65.45
2	(D)-**3b**	12.945	2979977	34.55

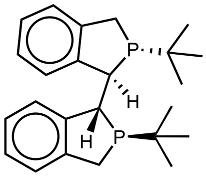

(1R, 1'R, 2S, 2'S)-DuanPhos
CAS Number: 528814-26-8

82:18 L:D

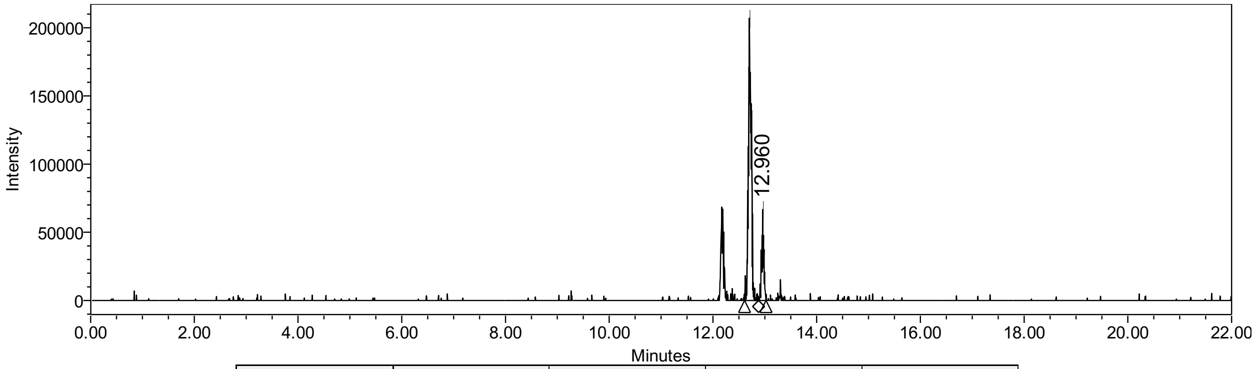

	Name	Retention Time	Area	% Area
1	(L)-**3b**	12.702	738274	82.30
2	(D)-**3b**	12.960	158819	17.70

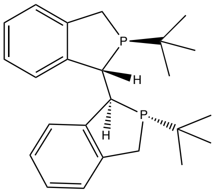

(1S, 1'S, 2R' 2'R)-DuanPhos
CAS number: 795290-34-5

L:D 66:34

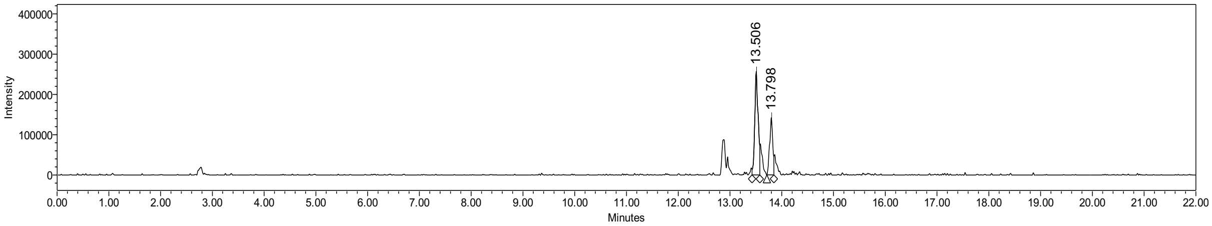

	Name	Retention Time	Area	% Area
1	(L)-**3b**	13.506	1146283	66.37
2	(D)-**3b**	13.798	580763	33.63

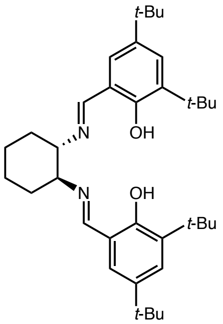

(S,S)-Jacobsen's Ligand
CAS No. 135616-36-3

68:32 L:D

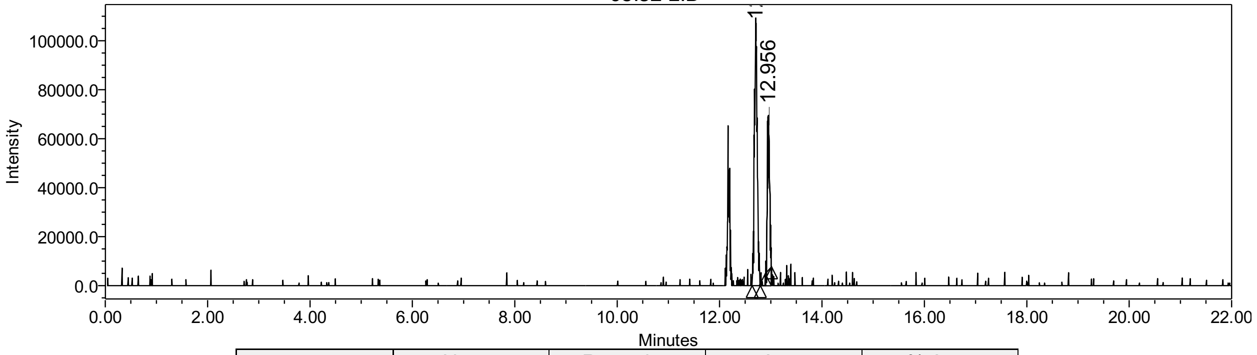

	Name	Retention Time	Area	% Area
1	(L)-**3b**	12.708	394421	68.26
2	(D)-**3b**	12.956	183433	31.74

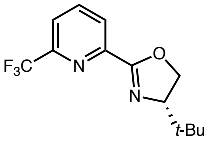

(S)-4-(tert-Butyl)-2-(5-(trifluoromethyl)pyridin-2-yl)-4,5-dihydrooxazole

CAS No. 1416819-91-4

71:29 L:D

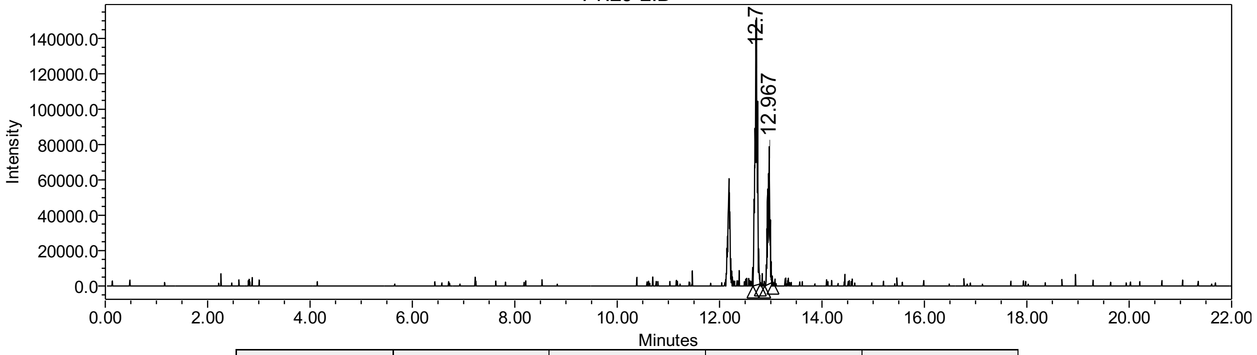

	Name	Retention Time	Area	% Area
1	(L)-**3b**	12.714	450545	70.51
2	(D)-**3b**	12.967	188478	29.49

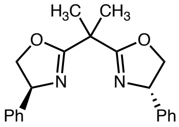

(4S,4S)-2,2-(Propane-2,2-diyl)bis(4-phenyl-4,5-dihydrooxazole)

CAS No. 131457-46-0

68:32 L:D

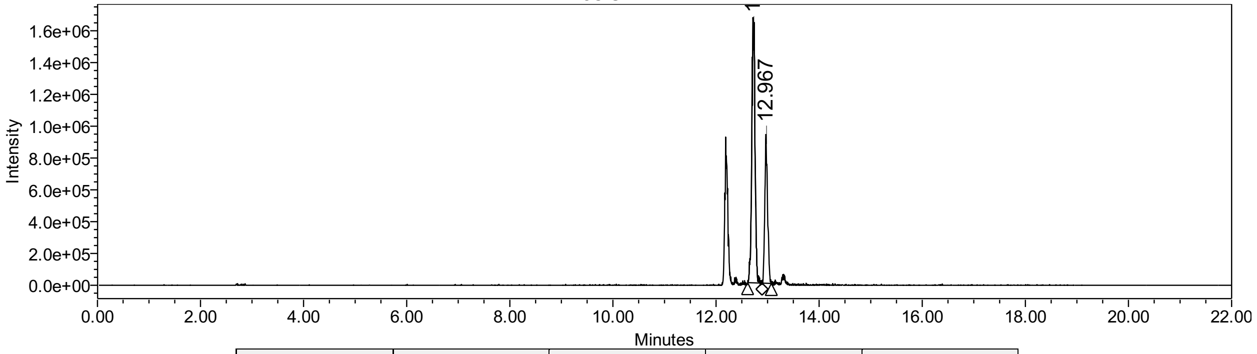

	Name	Retention Time	Area	% Area
1	(L)-**3b**	12.721	7332761	68.28
2	(D)-**3b**	12.967	3407225	31.72

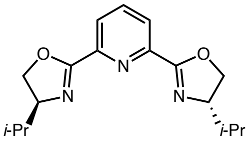

2,6-Bis((S)-4-isopropyl-4,5-dihydrooxazol-2-yl) pyridine

CAS No. 118949-61-4

69:31 L:D

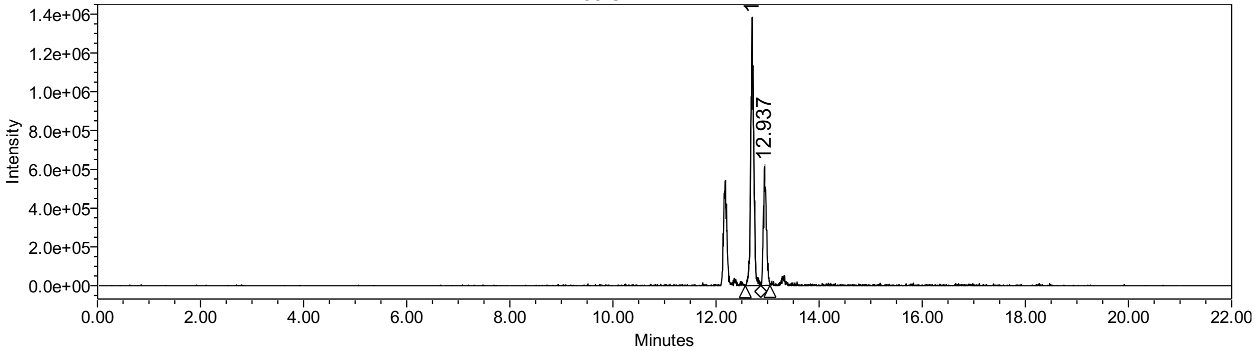

	Name	Retention Time	Area	% Area
1	(L)-**3b**	12.700	5361455	69.49
2	(D)-**3b**	12.937	2354242	30.51

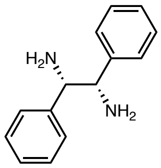

(1S,2S)-(−)-1,2-Diphenylethylenediamine

CAS No. 29841-69-8

69:31 L:D

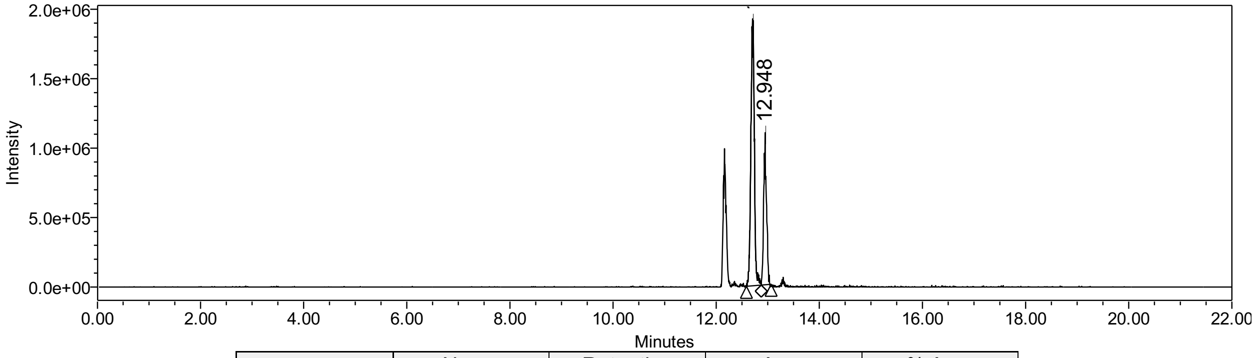

	Name	Retention Time	Area	% Area
1	(L)-**3b**	12.705	8993101	69.10
2	(D)-**3b**	12.948	4021701	30.90

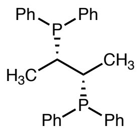

(S,S)-Chiraphos

CAS No. 64896-28-2

31:69 L:D

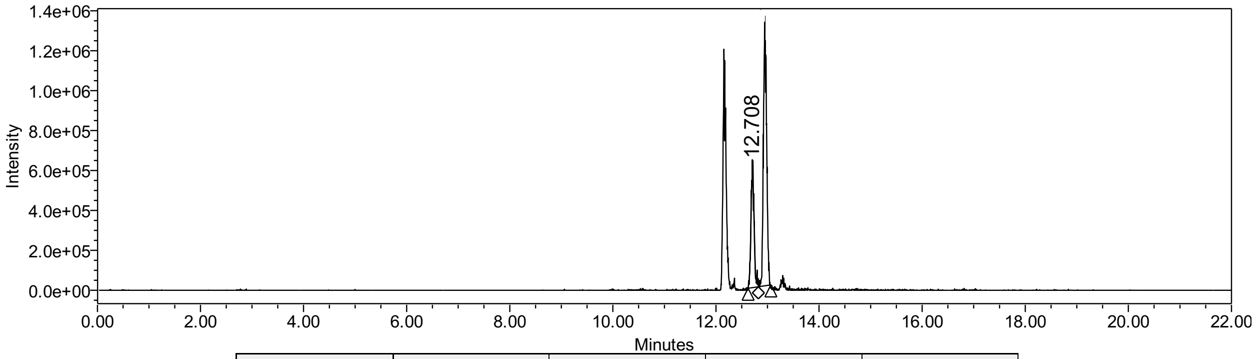

	Name	Retention Time	Area	% Area
1	(L)-**3b**	12.708	2529436	30.57
2	(D)-**3b**	12.944	5745828	69.43

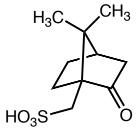

(1S)-(+)-10-Camphorsulfonic acid
CAS No. 3144-16-9

56:44 L:D

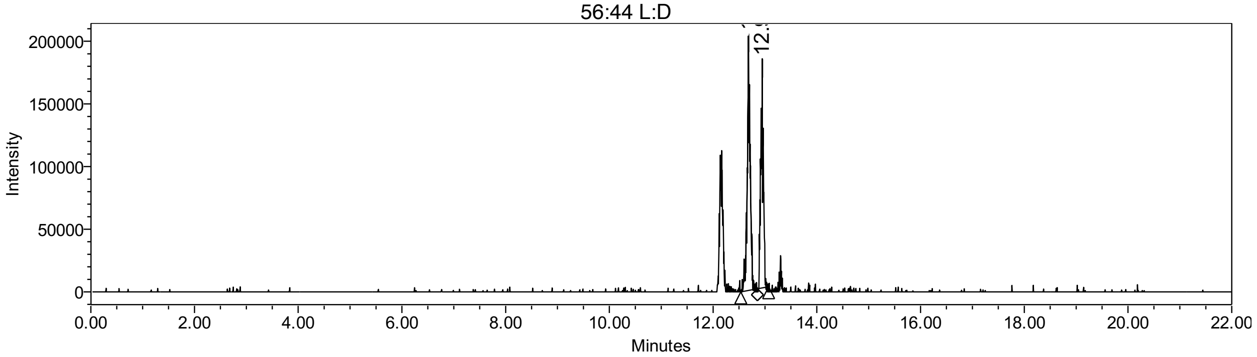

	Name	Retention Time	Area	% Area
1	(L)-**3b**	12.681	750420	56.37
2	(D)-**3b**	12.948	580915	43.63

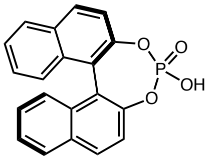

(R)-(–)-1,1′-Binaphthyl-2,2′-diyl hydrogenphosphate

CAS No. 39648-67-4

49:51 L:D

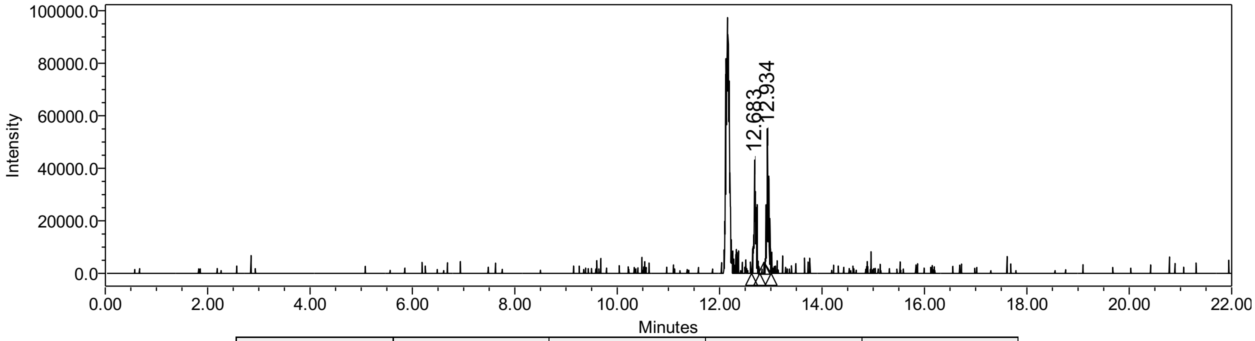

	Name	Retention Time	Area	% Area
1	(L)-**3b**	12.683	99632	49.06
2	(D)-**3b**	12.934	103430	50.94

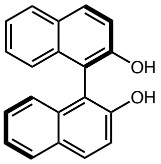

(R)-(+)-1,1′-Bi(2-naphthol)

CAS No. 18531-94-7

60:40 L:D

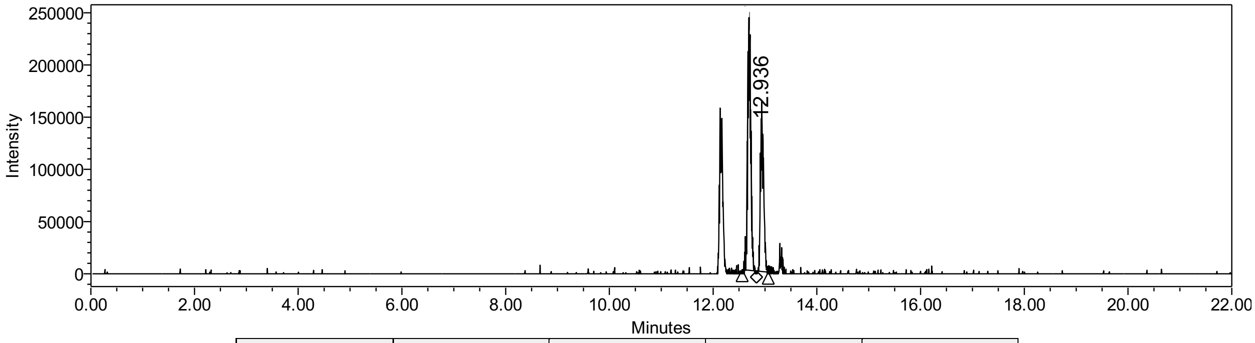

	Name	Retention Time	Area	% Area
1	(L)-**3b**	12.690	952143	59.72
2	(D)-**3b**	12.936	642134	40.28

Authentic (L)-**3b**

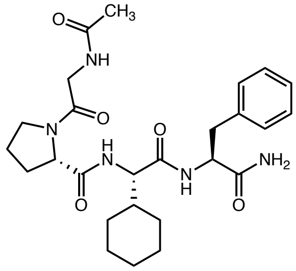

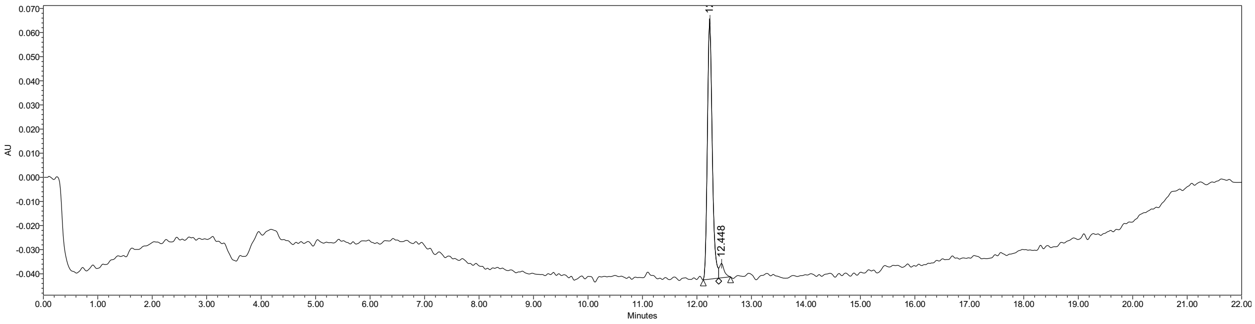

	Name	Retention Time	Area	% Area
1	Authentic (L)-**3b**	**12.234**	662127	94.76
2		12.448	36617	5.24

Authentic (D)-**3b**

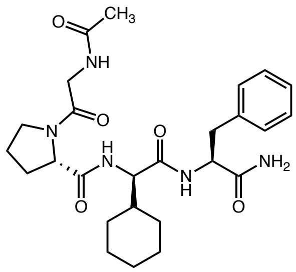

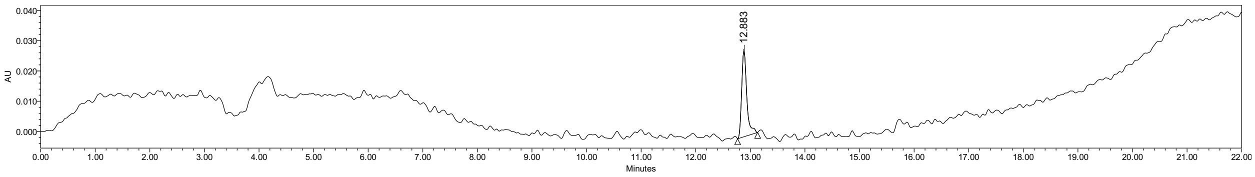

	Name	Retention Time	Area	% Area
1	Authentic (D)-**3b**	**12.883**	184988	100.00

E. Peptide Characterization

a) Starting Material Characterization

Ac-GPCF-NH$_2$

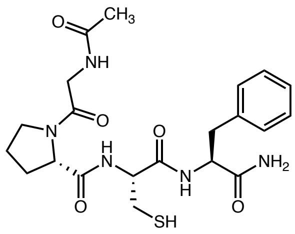

^{1}H NMR (CD_3CN, 400 MHz): d 7.38 (1H, br. d *J*=8.6 Hz), 7.32-7.17 (5H, m), 6.97 (1H, br. s), 6.63 (1H, br. s), 5.85 (1H, br. s), 4.43 (1H, q, *J*=4.6 Hz), 4.28 (1H, dd, *J*=8.5, 5.4 Hz), 4.23-4.10 (2H, m), 3.84 (1H, dd, *J*=16.9, 4.3 Hz), 3.77-3.68 (1H, m), 3.63-3.53 (1H, m), 3.25 (1H, dd, *J*=13.9, 4.7 Hz), 2.90 (1H, dd, *J*=14.0, 10.0 Hz), 2.81-2.61 (2H, m), 2.06-1.96 (2H, m), 1.84 (3H, s).

Ac-GP(Dha)F-NH$_2$

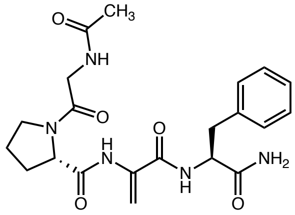

^{1}H NMR (CD_3CN, 400 MHz): d 8.56 (1H, br. s), 7.37-7.16 (6H, m), 6.74 (2H, br. s), 5.89-5.78 (1H, m), 5.70 (1H, s), 5.46 (1H, s), 4.51 (1H, ddd, *J*=9.9, 8.3, 4.6 Hz), 4.33 (1H, dd, *J*=8.0, 4.1 Hz), 4.09-3.89 (2H, m), 3.65-3.47 (2H, m), 3.26 (1H, dd, *J*=14.0, 4.6 Hz), 2.93 (1H, dd, *J*=14.0, 10.0 Hz), 2.00 (3H, ddq, *J*=8.9, 4.1, 2.2 Hz), 1.87 (3H, s).

Ac-GP(ΔAlaBr)F-NH$_2$

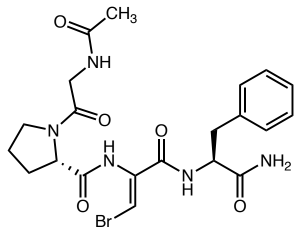

^{1}H NMR (CD_3CN, 400 MHz): d 8.31 (1H, s), 7.52 (1H, d, *J*=8.5 Hz), 7.29-7.17 (6H, m), 7.15 (1H, d, *J*=1.0 Hz), 6.81 (2H, d, *J*=14.0 Hz), 5.83 (1H, s), 4.43 (1H, ddd, *J*=10.4, 8.5, 4.3 Hz), 4.30 (1H, dd, *J*=8.3, 5.6 Hz), 4.19 (1H, dd, *J*=17.3, 5.9 Hz), 3.90 (1H, dd, *J*=17.3, 4.3 Hz), 3.65 (2H, ddt, *J*=41.9, 10.2, 6.3 Hz), 3.29 (1H, dd, *J*=14.0, 4.3 Hz), 2.92 (1H, dd, *J*=14.0, 10.4), 2.34-2.23 (2H, m), 2.13-1.96 (4H, m), 1.81 (3H, s).

Ac-GP(ΔCys^{n}Pr)F-NH$_2$

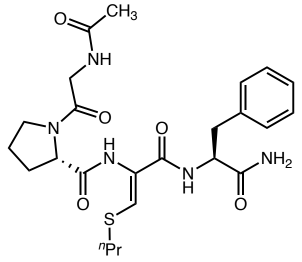

^{1}H NMR (CD_3CN, 400 MHz): d 8.06 (1H, s), 7.39-7.32 (2H, m), 7.28-7.13 (6H, m), 6.94-6.82 (2H, m), 5.96 (1H, s), 4.43 (1H, ddd, *J*=10.5, 8.6, 4.2 Hz), 4.29-4.15 (2H, m), 3.89 (1H, dd, *J*=17.3, 4.1 Hz), 3.63 (2H, ddt, *J*=39.3, 10.1, 6.6 Hz), 3.27 (1H, dd, *J*=13.9, 4.3 Hz), 2.91 (1H, dd, *J*=13.9, 10.4 Hz), 2.79 (2H, t, *J*=7.3 Hz), 2.35-2.20 (5H, m), 2.13-1.87 (5H, m), 1.78 (3H, s), 0.94 (3H, t, *J*=7.3 Hz).

Ac-GP(ΔSulf^{n}Pr)F-NH$_2$

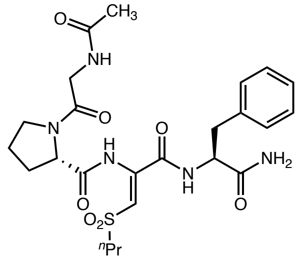

^{1}H NMR (CD_3CN, 400 MHz): d 9.67 (1H, br. s), 7.38 (1H, d, *J*=8.6 Hz), 7.36-7.23 (6H, m), 6.92 (1H, s), 6.71 (1H, s), 5.93 (1H, s), 5.55 (1H, s), 4.57 (1H, ddd, *J*=10.2, 8.6, 4.6 Hz), 4.36 (1H, dd, *J*=8.7, 4.5 Hz), 4.03 (2H, dd, *J*=8.4, 5.0 Hz), 3.61 (2H, qt, *J*=9.9, 6.5 Hz), 3.36 (1H, dd, *J*=14.1, 4.7 Hz), 3.07 (2H, ddd, *J*=9.5, 6.3, 1.3 Hz), 2.93 (1H, dd, *J*=14.1, 10.2 Hz), 2.22 (5H, q, *J*=7.8 Hz), 2.04-1.98 (3H, m), 1.91 (3H, s), 1.77-1.66 (2H, s), 1.04 (3H, t, *J*=7.4 Hz).

b) Product Characterization

3a

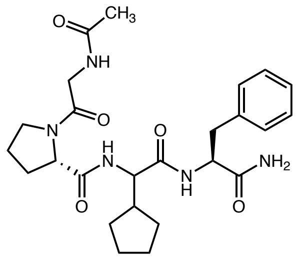

Crude Reaction – Step 1

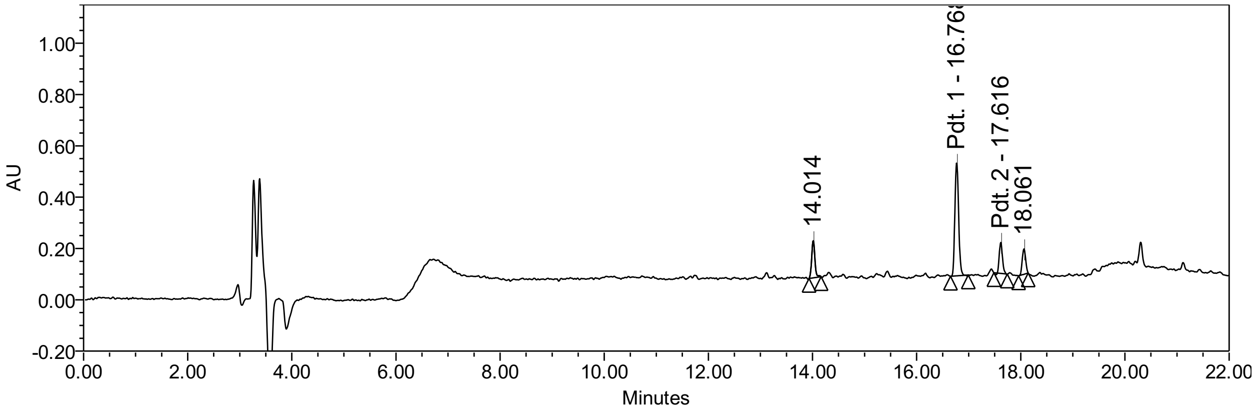

	Name	Retention Time	Area	% Area
1		14.014	617805	16.16
2	**Pdt. 1**	16.768	2215783	**57.95**
3	**Pdt. 2**	17.616	538511	**14.08**
4		18.061	451433	11.81

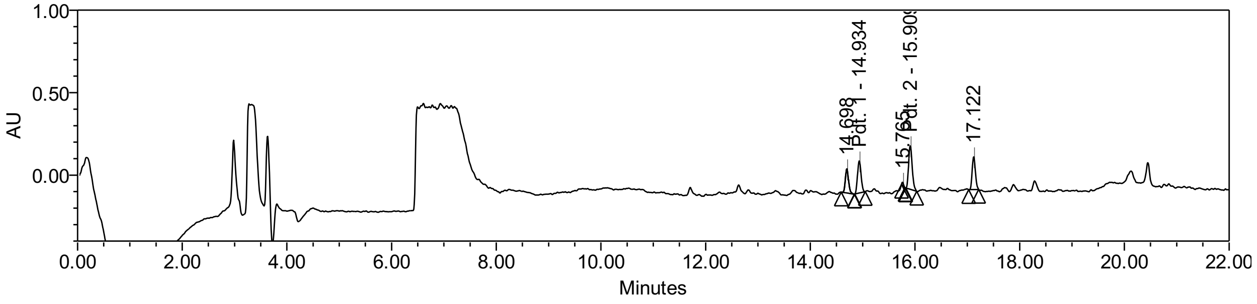

Crude Reaction – Step 2

	Name	Retention Time	Area	% Area
1		14.698	627899	16.97
2	**Pdt. 1**	14.934	920367	**24.88**
3		15.765	38068	1.03
4	**Pdt. 2**	15.909	1260308	**34.07**
5		17.122	852825	23.05

Purified **3a**

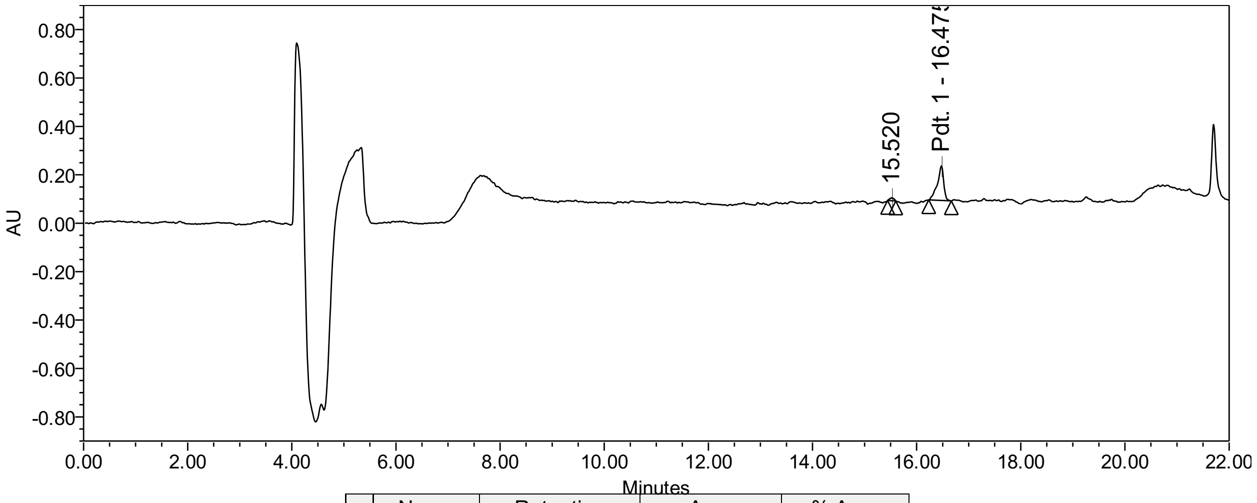

	Name	Retention Time	Area	% Area
1		15.520	67375	5.27
2	**Pdt. 1**	16.475	1210250	**94.73**

Purified **3a** – ESI+ Trace

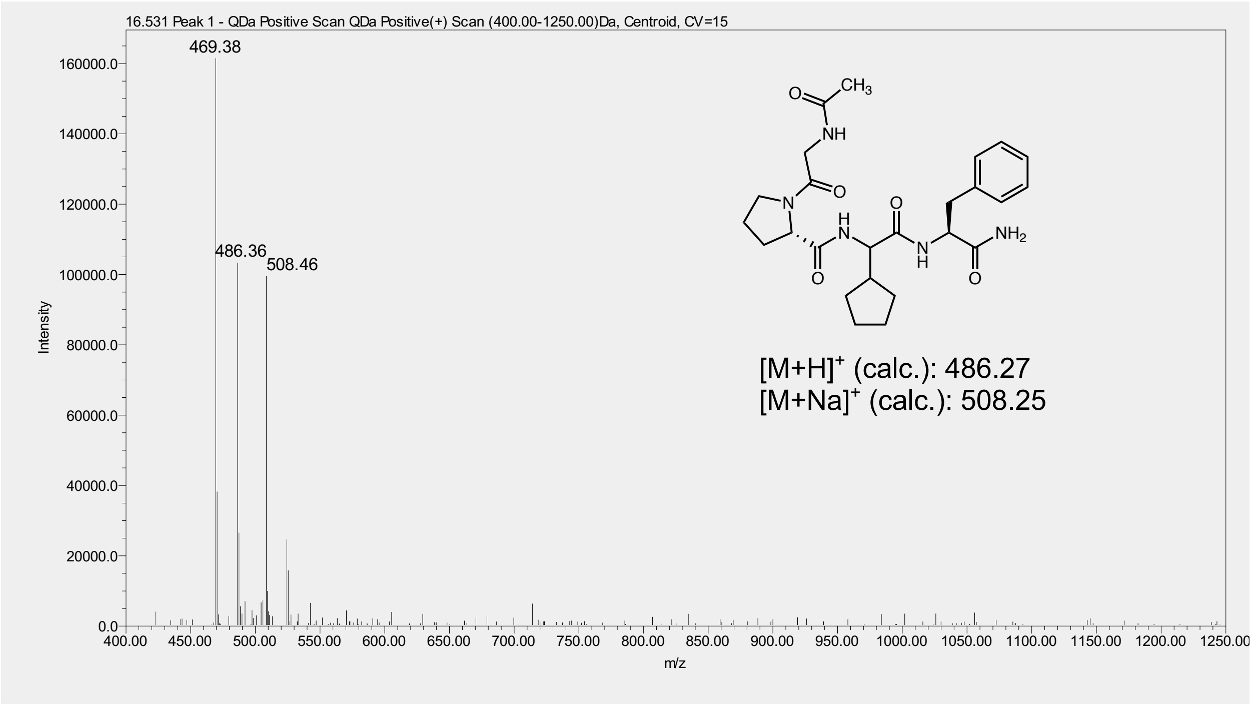

3b

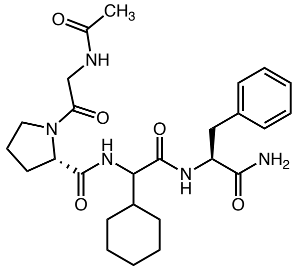

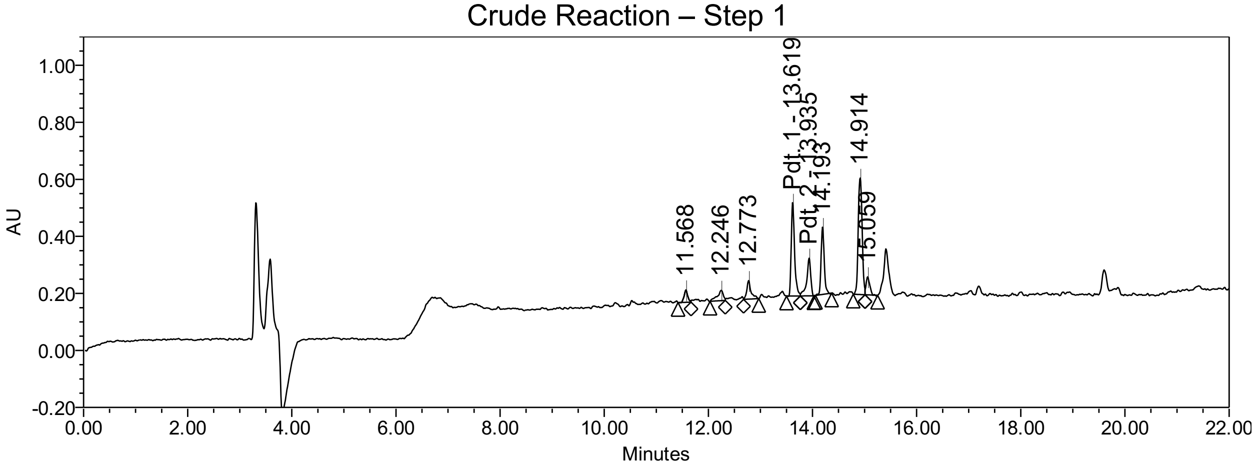

	Name	Retention Time	Area	% Area
1		11.568	209528	3.24
2		12.246	289674	4.47
3		12.773	369431	5.71
4	**Pdt. 1**	13.619	1463750	**22.61**
5	**Pdt. 2**	13.935	704287	**10.88**
6		14.193	987844	15.26
7		14.914	2137210	33.01
8		15.059	311785	4.82

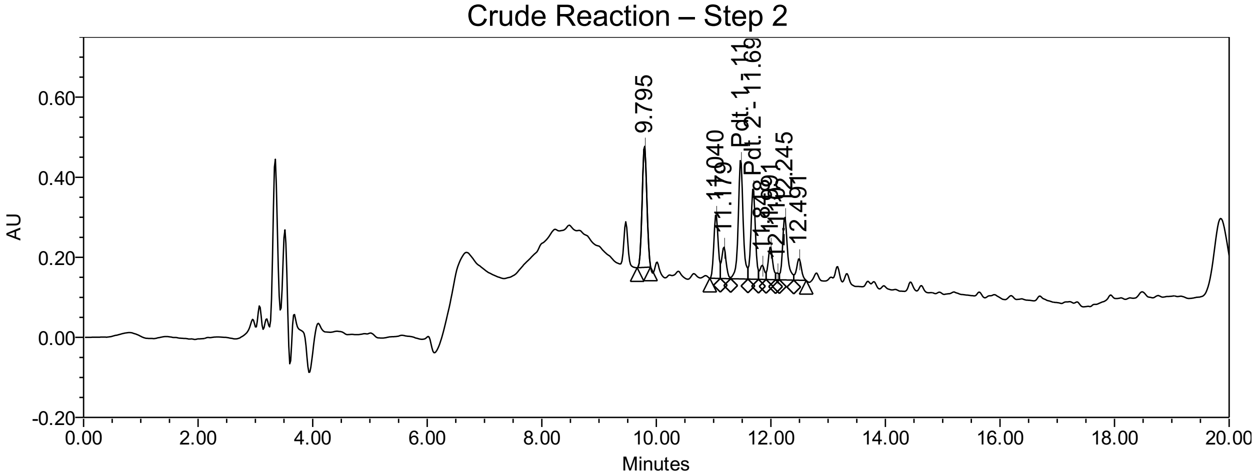

	Name	Retention Time	Area	% Area
1		9.795	1721882	21.47
2		11.040	835034	10.41

3		11.179	437702	5.46
4	**Pdt. 1**	11.473	1773520	**22.11**
5	**Pdt. 2**	11.690	1229659	**15.33**
6		11.848	207631	2.59
7		11.991	464296	5.79
8		12.110	64030	0.80
9		12.245	961813	11.99
10		12.491	325853	4.06

Purified **3b**

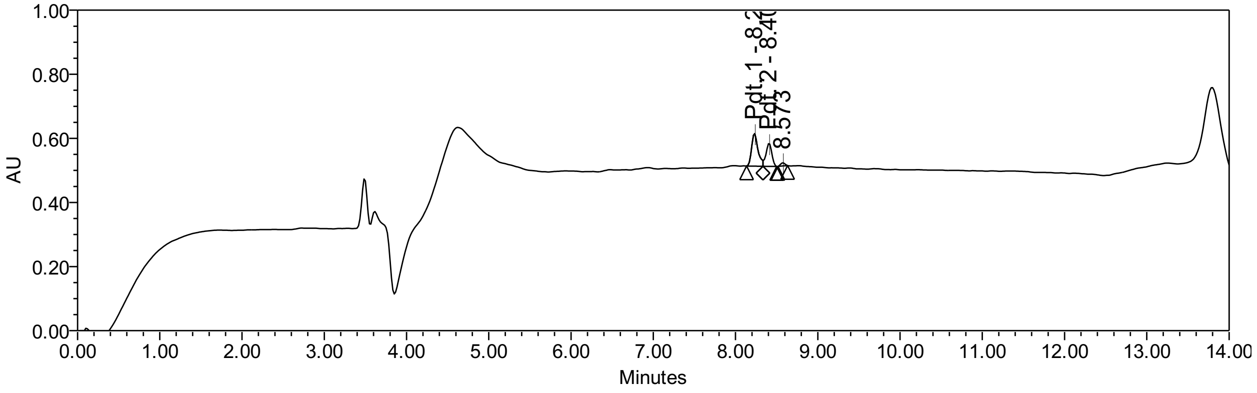

	Name	Retention Time	Area	% Area
1	**Pdt. 1**	8.230	553083	**58.54**
2	**Pdt. 2**	8.406	355642	**37.64**
3		8.573	36050	3.82

Purified **3b** – ESI+ Trace

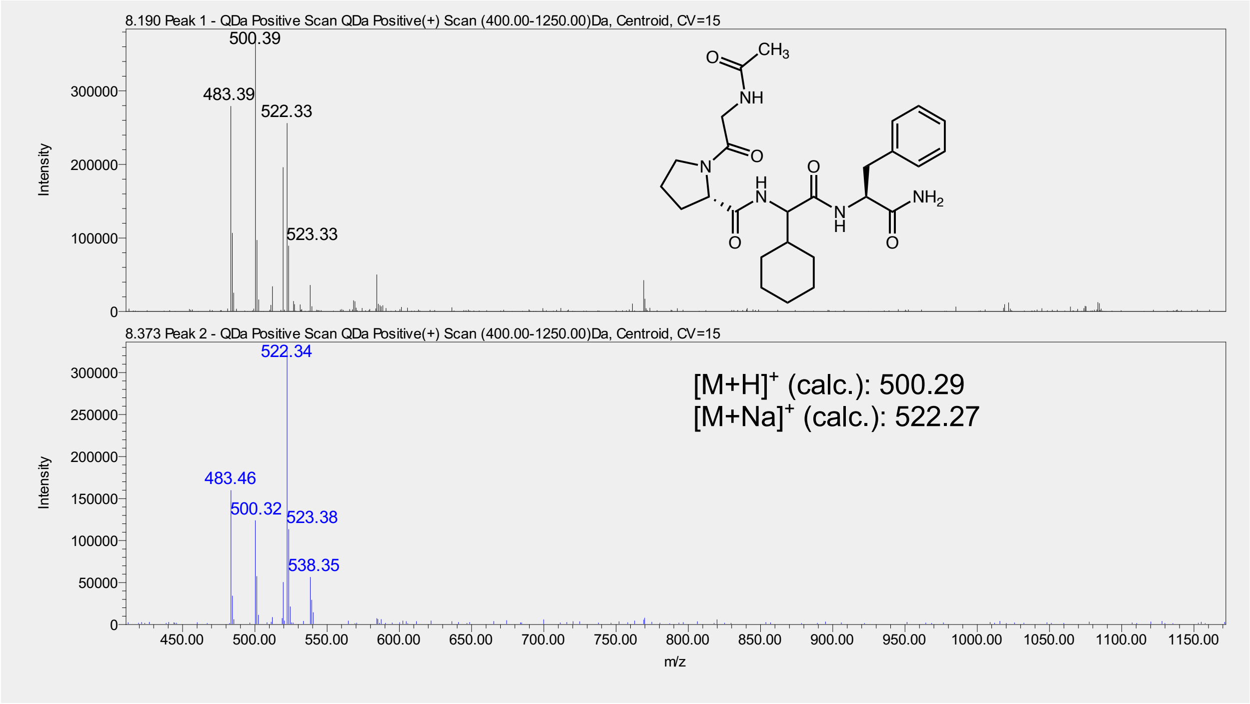

3c

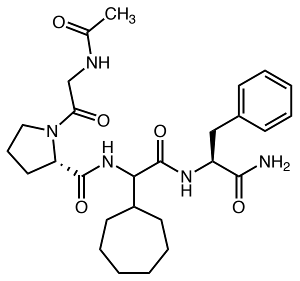

Crude Reaction – Step 1

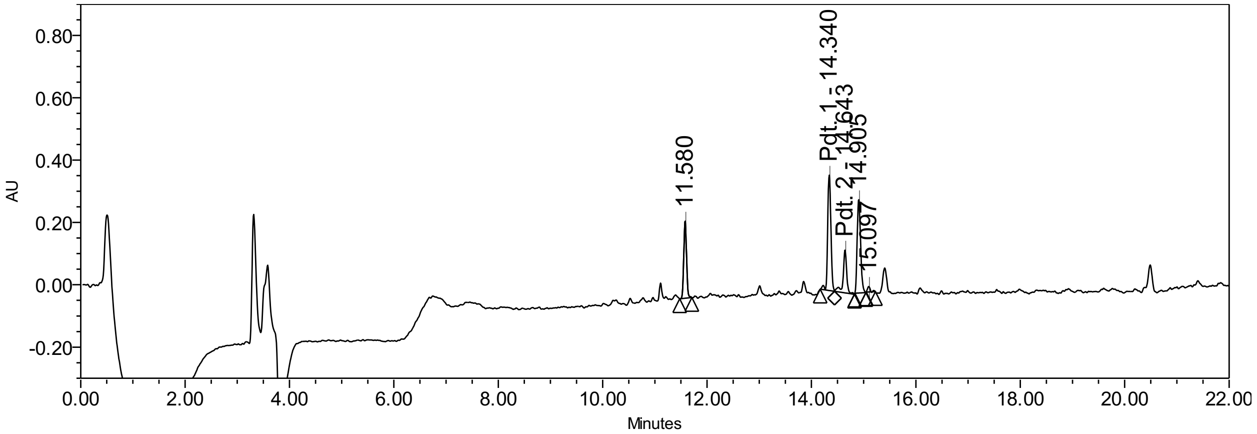

	Name	Retention Time	Area	% Area
1		11.580	919584	19.97
2	**Pdt. 1**	14.340	1600977	**34.77**
3	**Pdt. 2**	14.643	648128	**14.08**
4		14.905	1370340	29.76
5		15.097	65456	1.42

Crude Reaction – Step 2

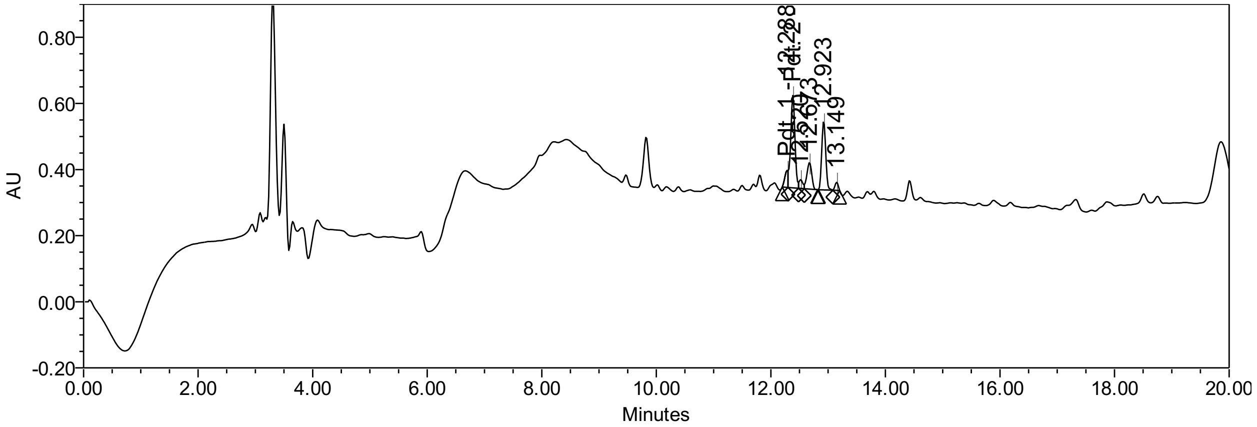

	Name	Retention Time	Area	% Area
1	**Pdt. 1**	12.288	186357	**5.63**
2	**Pdt. 2**	12.384	1448056	**43.71**
3		12.520	119513	3.61
4		12.673	463971	14.00

5		12.923	1002576	30.26
6		13.149	92487	2.79

Purified **3c**

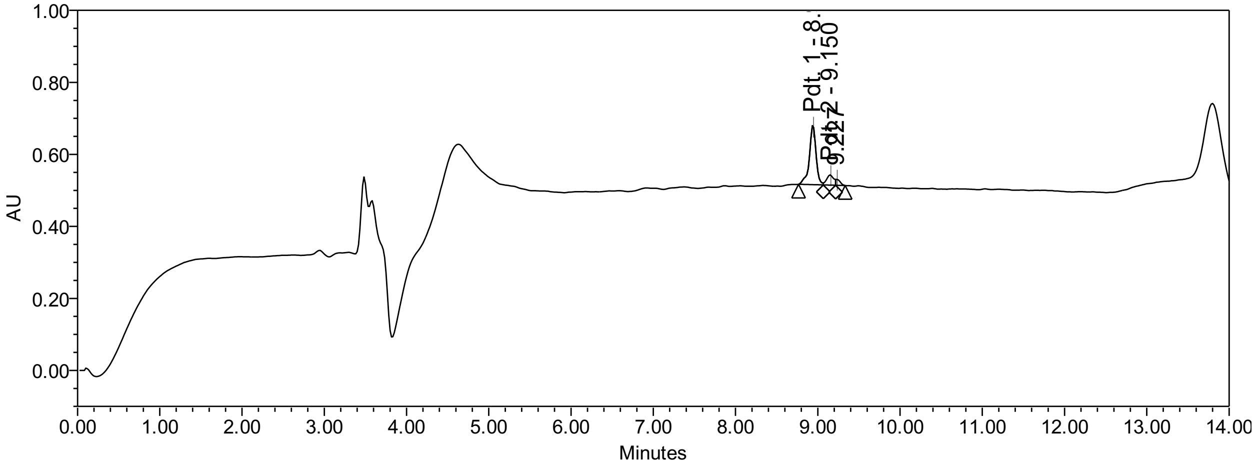

	Name	Retention Time	Area	% Area
1	**Pdt. 1**	8.939	930734	**80.22**
2	**Pdt. 2**	9.150	168685	**14.54**
3		9.227	60850	5.24

Purified **3c** – ESI+ Trace

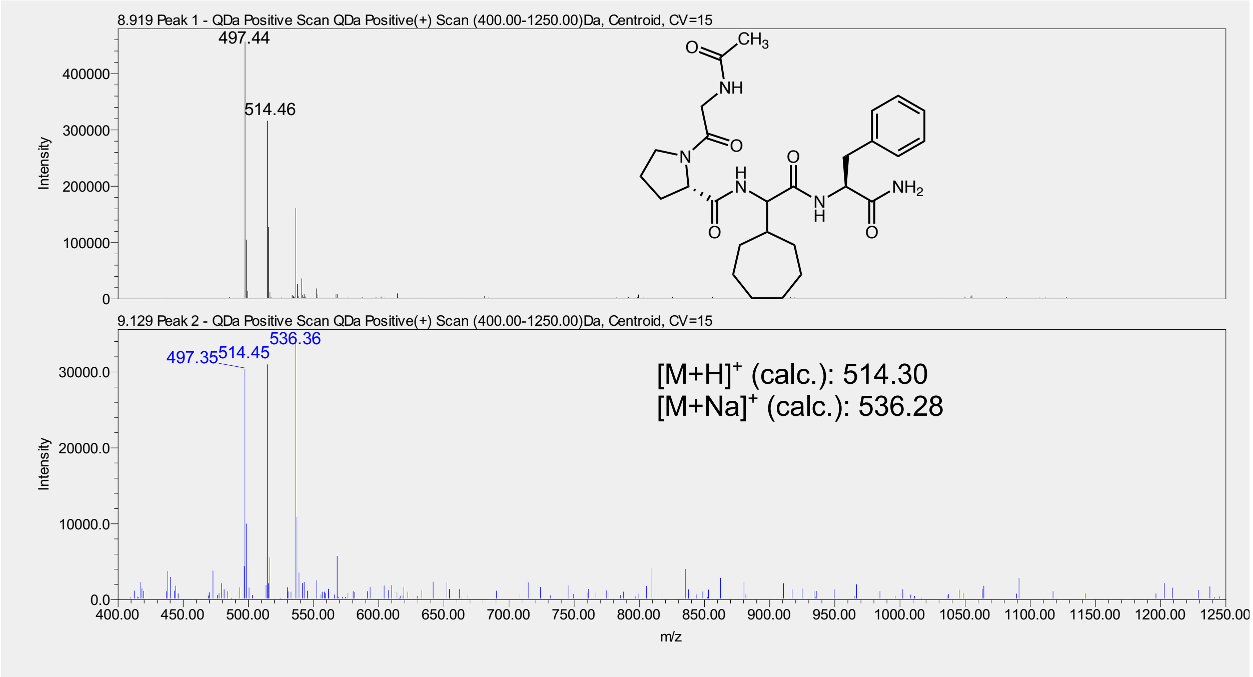

3d

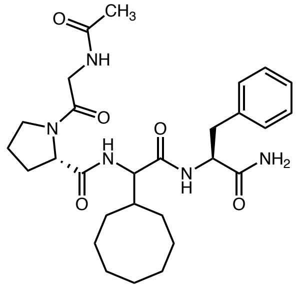

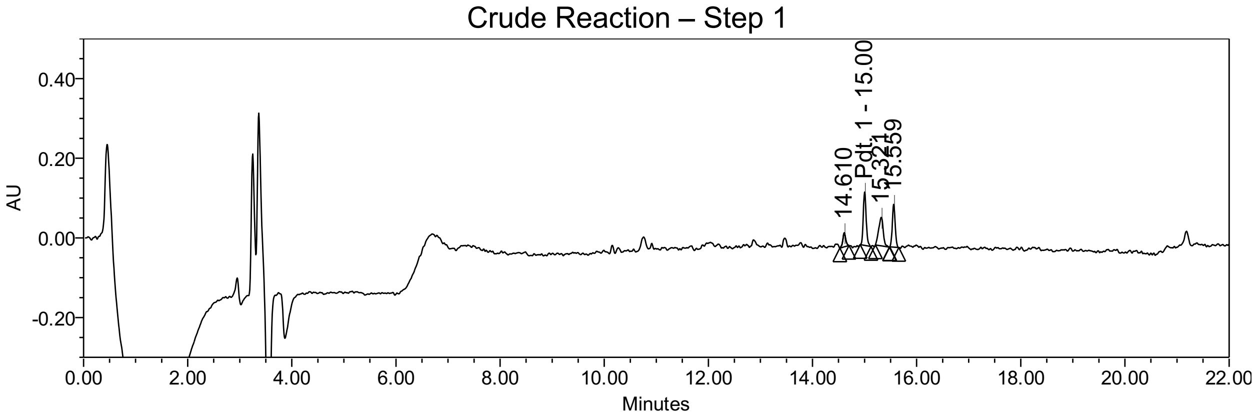

	Name	Retention Time	Area	% Area
1		14.610	136526	9.07
**2**	**Pdt. 1**	15.001	523012	**34.74**
3		15.321	437154	29.03
4		15.559	409016	27.16

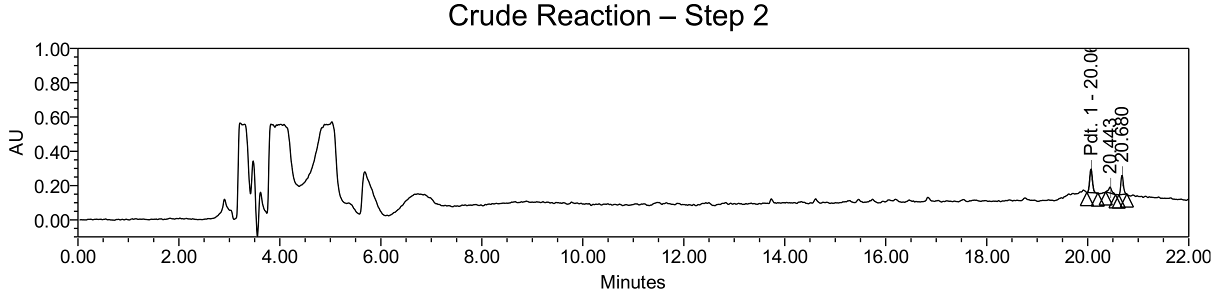

	Name	Retention Time	Area	% Area
1	**Pdt. 1**	20.066	520750	**48.47**
2		20.443	159086	14.81
3		20.680	394457	36.72

Purified **3d**

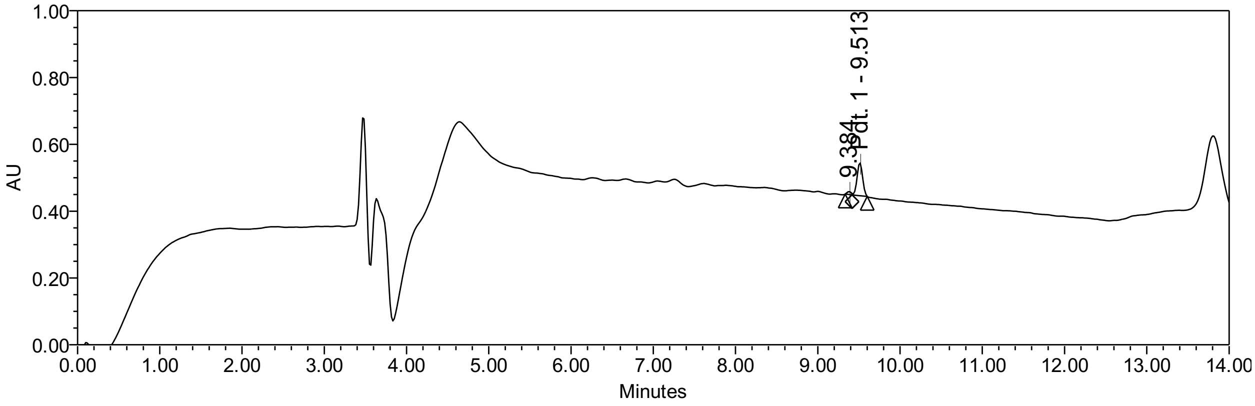

	Name	Retention Time	Area	% Area
1		9.384	28683	5.89
2	**Pdt. 1**	9.513	458532	**94.11**

Purified **3d** – ESI+ Trace

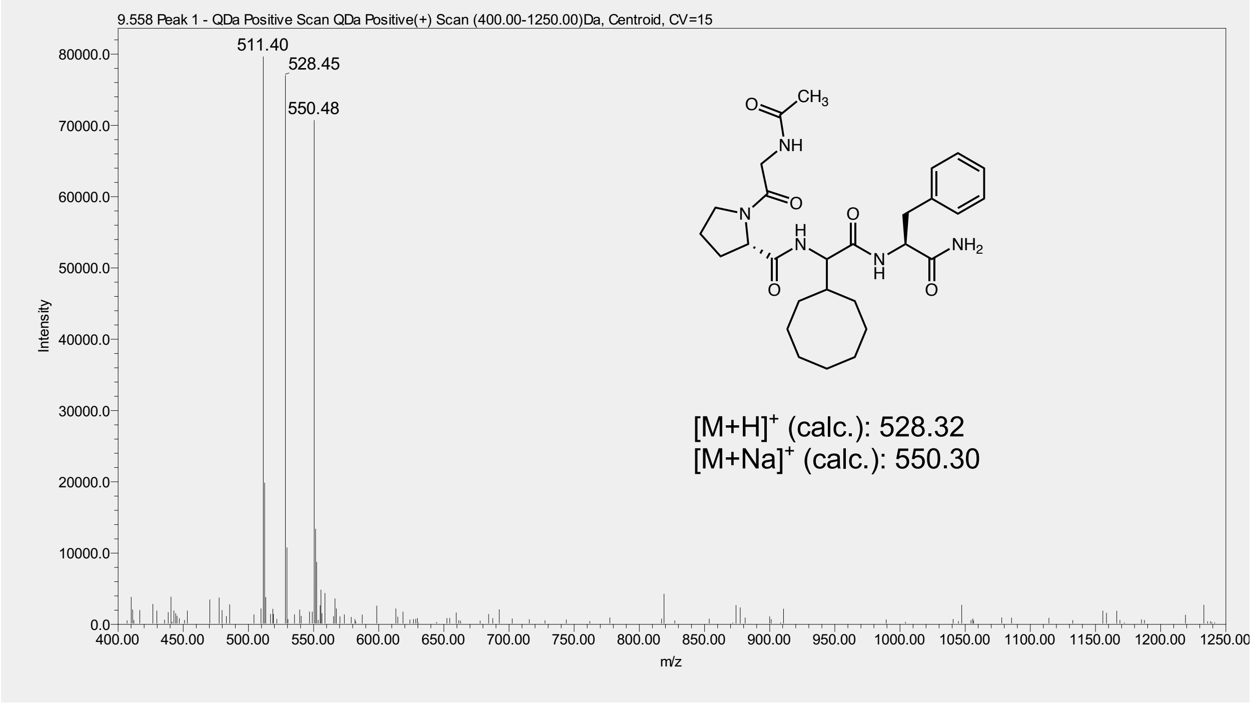

3e

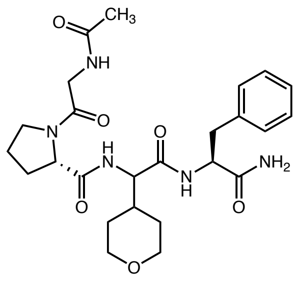

Crude Reaction – Step 1

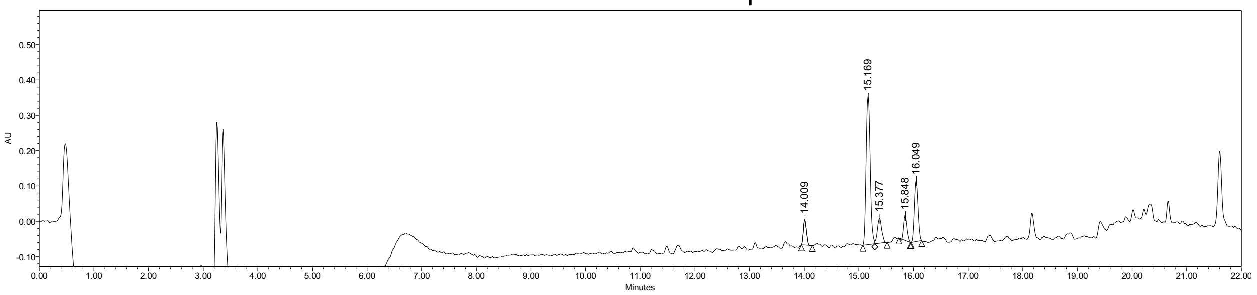

	Name	Retention Time	Area	% Area
1		14.009	273407	6.96
2	**Pdt. 1**	15.169	2180125	**55.51**
3		15.377	381655	9.72
4		15.848	302226	7.69
5		16.049	790372	20.12

Crude Reaction – Step 2

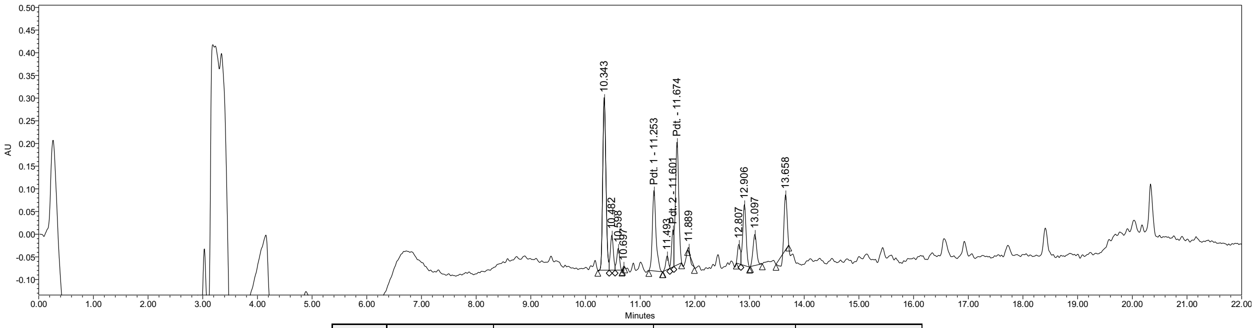

	Name	Retention Time	Area	% Area
1		10.343	1556408	26.15
2		10.482	289000	4.85
3		10.598	172715	2.90
4		10.697	6143	0.10
5	**Pdt. 1**	11.253	824818	**13.86**
6		11.493	105181	1.77
7		11.601	220702	3.71
8	**Pdt. 2**	11.674	1160443	**19.49**
9		11.889	43629	0.73
10		12.807	149582	2.51
11		12.906	573230	9.63
12		13.097	314697	5.29
13		13.658	536213	9.01

Purified **3e**

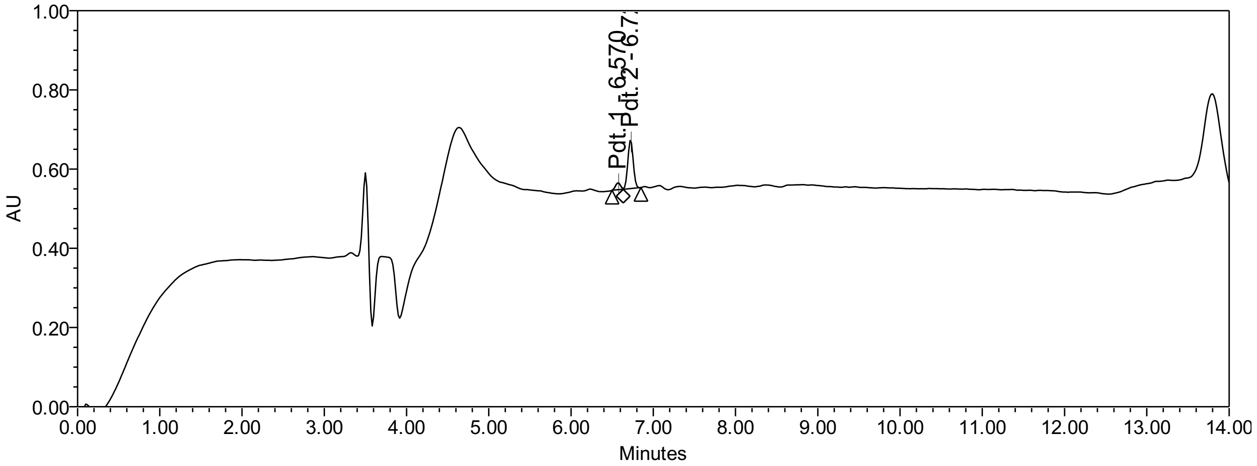

	Name	Retention Time	Area	% Area
1	**Pdt. 1**	6.570	73053	**11.56**
2	**Pdt. 2**	6.722	558710	**88.44**

Purified **3e** – ESI+ Trace

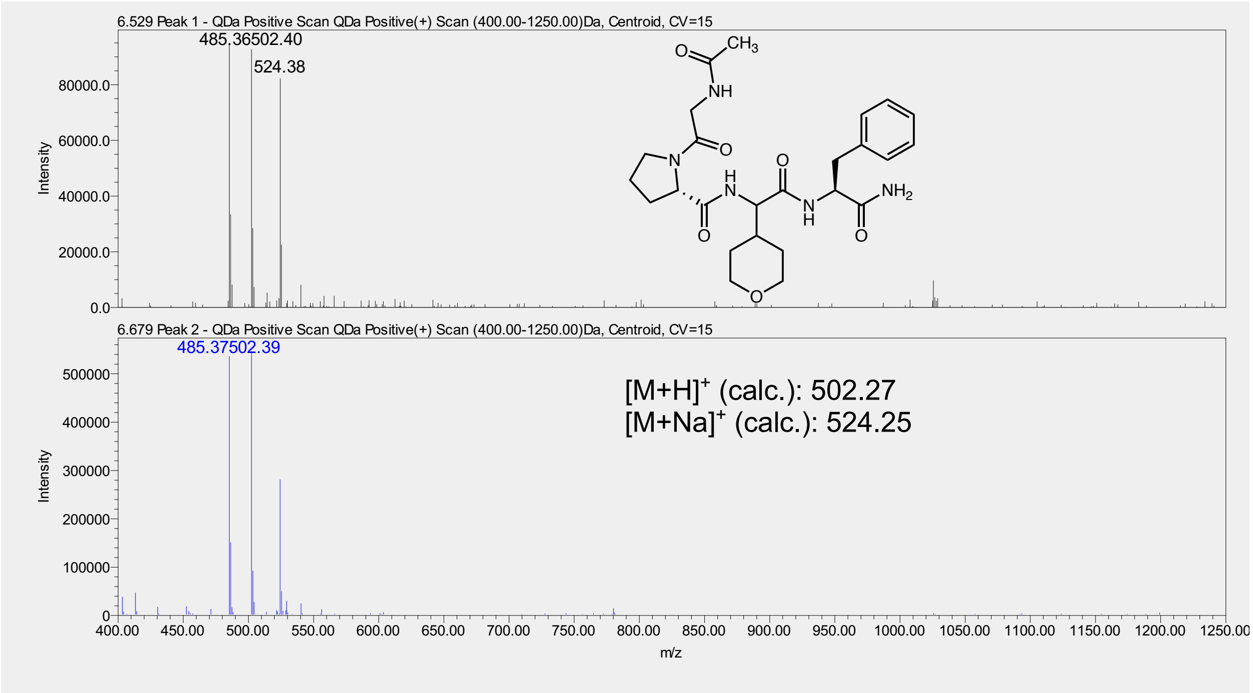

3f

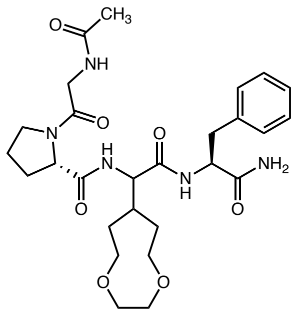

Crude Reaction – Step 1

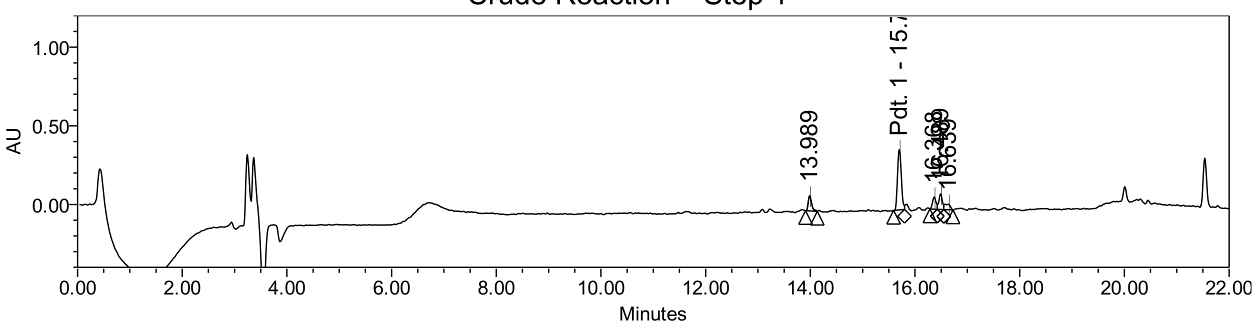

	Name	Retention Time	Area	% Area
1		13.989	429274	12.48
2	**Pdt. 1**	15.700	1930299	**56.11**
3		16.368	356726	10.37
4		16.489	454325	13.21
5		16.639	269417	7.83

Crude Reaction – Step 2

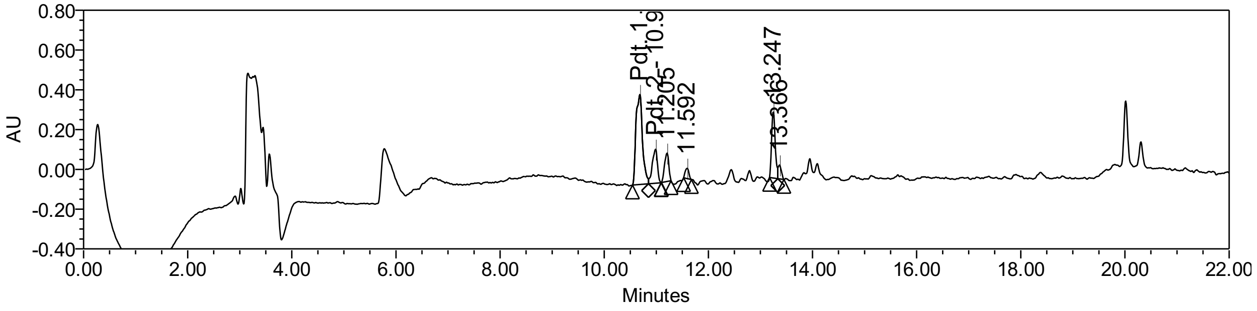

	Name	Retention Time	Area	% Area
1	**Pdt. 1**	10.683	3922283	**48.59**
2	**Pdt. 2**	10.985	1168749	**14.48**
3		11.205	804913	9.97
4		11.592	253828	3.14
5		13.247	1622644	20.10
6		13.366	300277	3.72

Purified **3f**

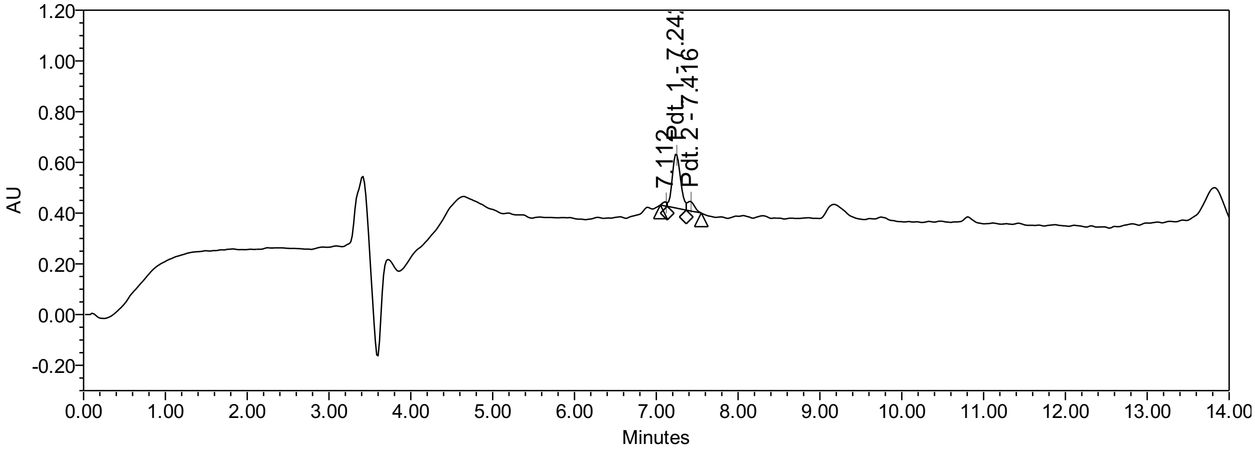

	Name	Retention Time	Area	% Area
1		7.112	54054	3.01
2	**Pdt. 1**	7.242	1510714	**84.03**
3	**Pdt. 2**	7.416	232957	**12.96**

Purified **3f** – ESI+ Trace

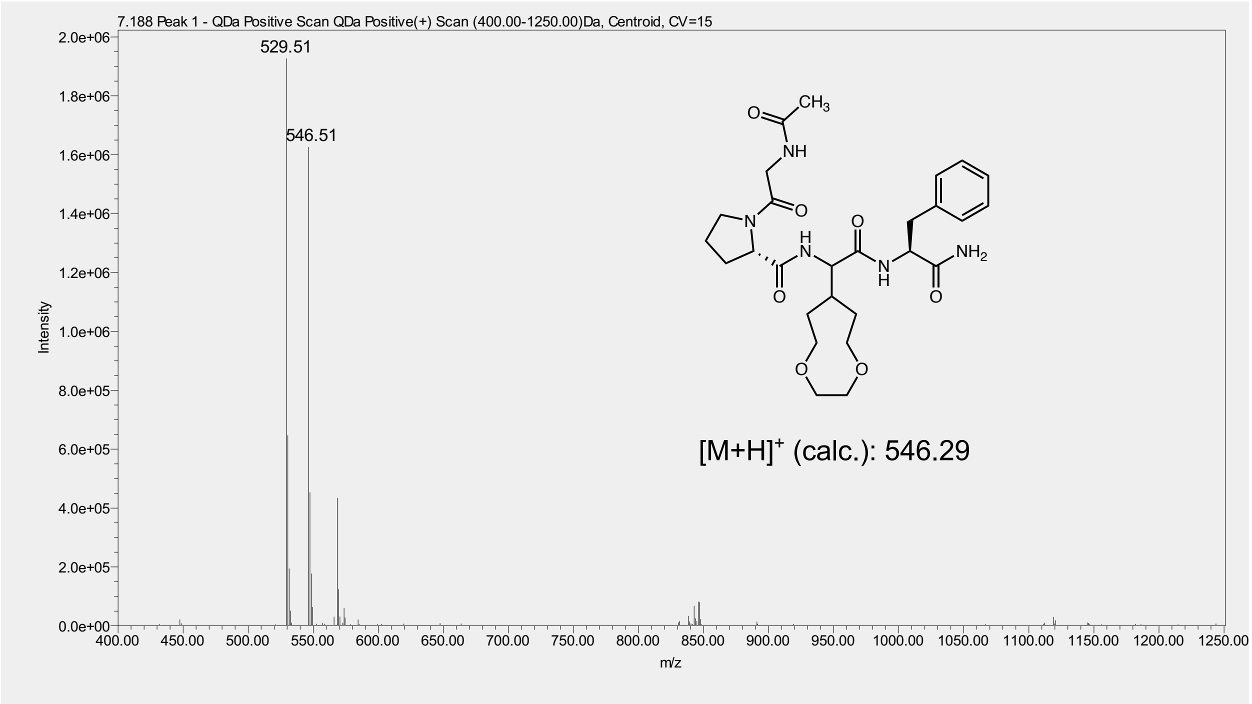

3g

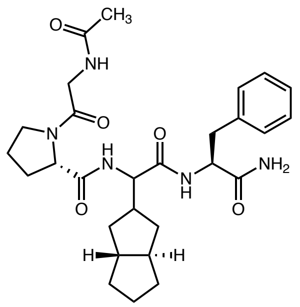

Crude Reaction – Step 1

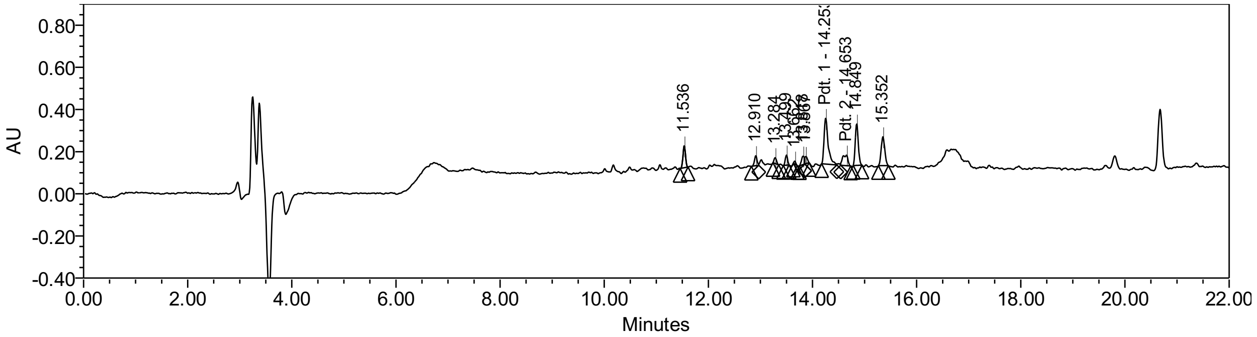

	Name	Retention Time	Area	% Area
1		11.536	346270	8.55
2		12.910	161902	4.00
3		13.284	87904	2.17
4		13.499	146096	3.61
5		13.662	32111	0.79
6		13.818	151190	3.73
7		13.867	111788	2.76
8	**Pdt. 1**	14.253	1197632	**29.58**
9	**Pdt. 2**	14.653	348231	**8.60**
10		14.849	802337	19.81
11		15.352	663911	16.40

Crude Reaction – Step 2

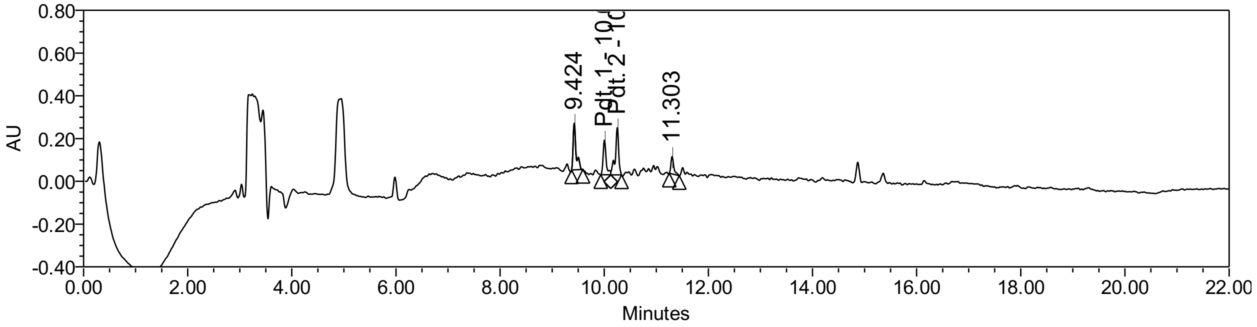

	Name	Retention Time	Area	% Area
1		9.424	871906	30.69
2	**Pdt. 1**	10.004	658453	**23.18**
3	**Pdt. 2**	10.251	997037	**35.09**
4		11.303	313794	11.04

Purified **3g**

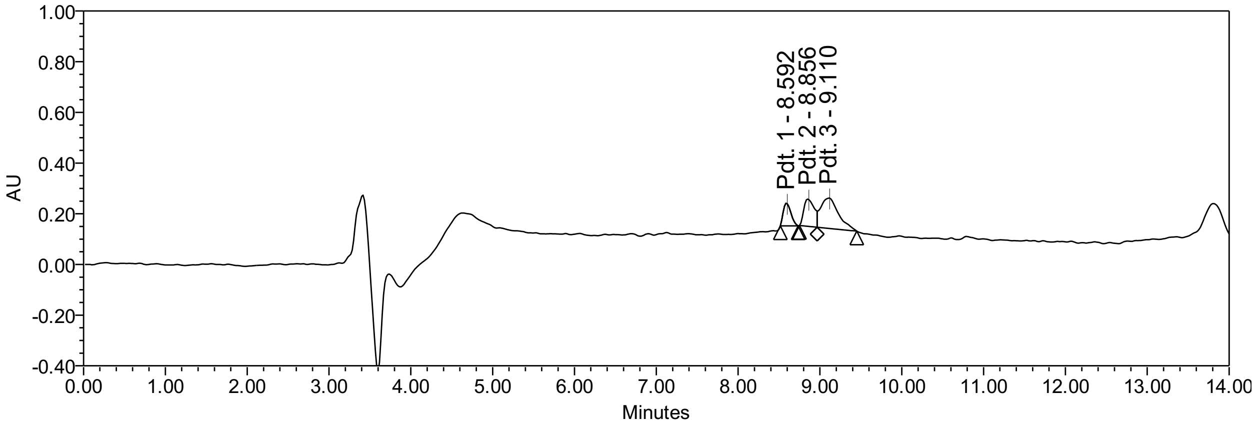

	Name	Retention Time	Area	% Area
1	**Pdt. 1**	8.592	581100	**17.31**
2	**Pdt. 2**	8.856	952631	**28.38**
3	**Pdt. 3**	9.110	1823031	**54.31**

Purified **3g** – ESI+ Trace

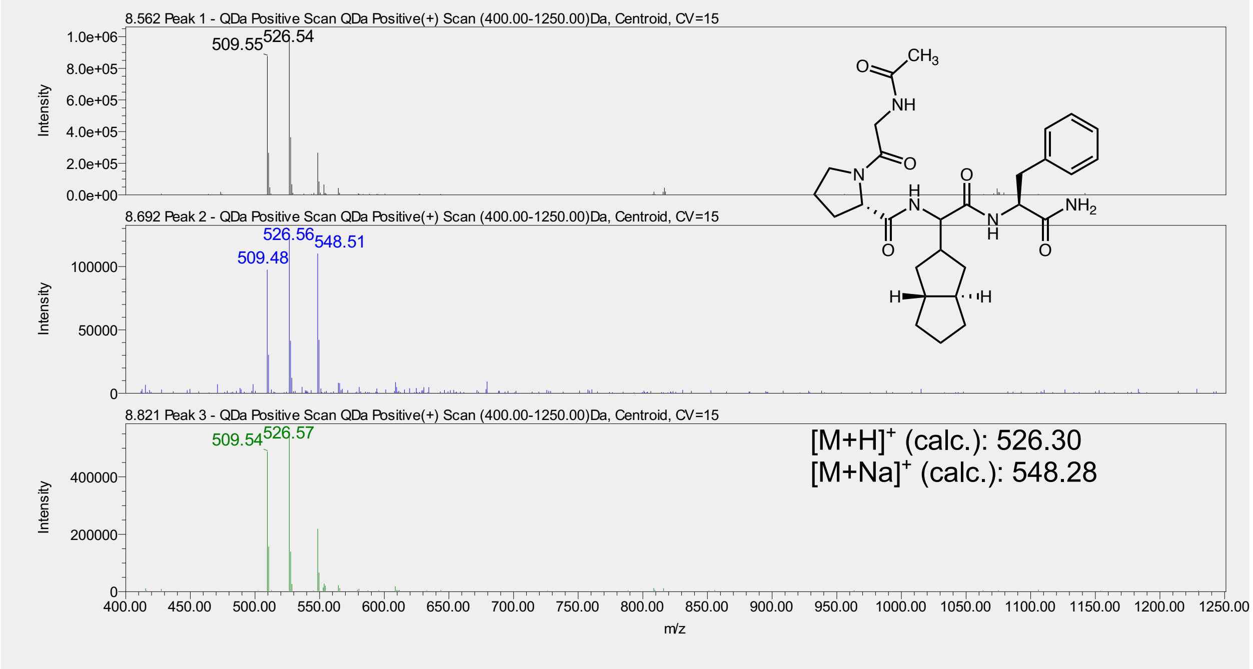

F. Mechanistic Studies

UV-Vis and Fluorescence Studies

Absorbance spectra were acquired on a Shimadzu UV-2600i spectrophotometer and fluorescence spectra using a QuantaMaster Luminescence Spectrometer (Photon Technologies, Inc.). Fluorescence samples, including those for Stern-Volmer quenching, were degassed with N_2 prior to acquisition. Spectra were acquired in the indicated aqueous solvent at the given concentration, which varied depending on solubility. For quenching studies, an excitation wavelength of 510 nm for pyronin Y was used to avoid overlap with the fluorescence signal, and 430 nm was used for CoTMPP (no fluorescence could be observed when used as a saturated solution in aq. DMF upon excitation above 500 nm). Stern-Volmer quenching was conducted with 0.02 mM pyronin Y and 0.1 mM CoTMPP.

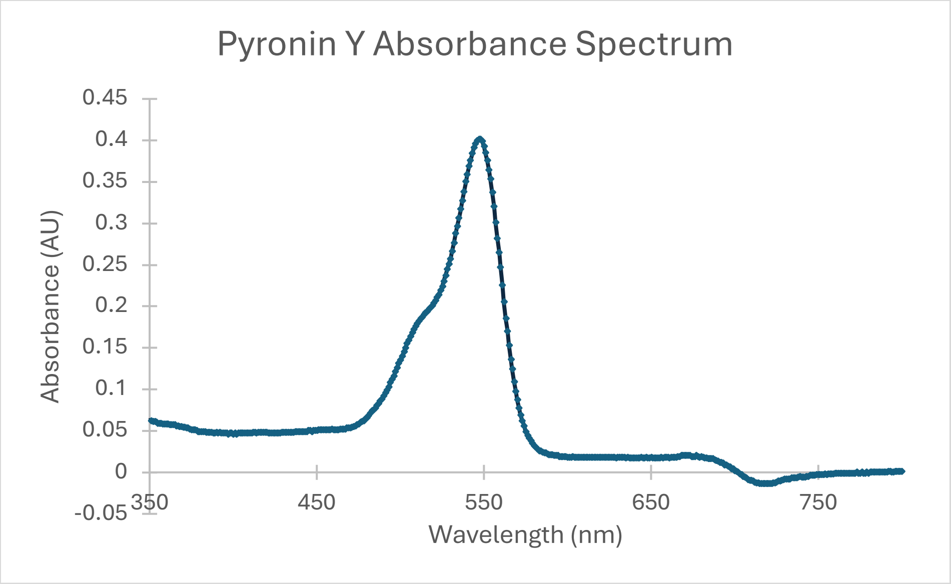

4 μM Pyronin Y, 1:1 H_2O:MeCN

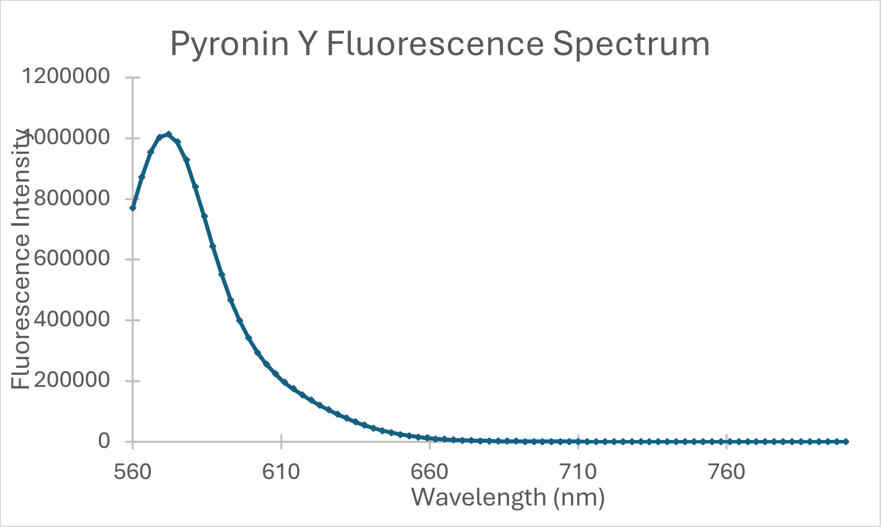

0.02 mM Pyronin Y, 1:1 H_2O:MeCN.

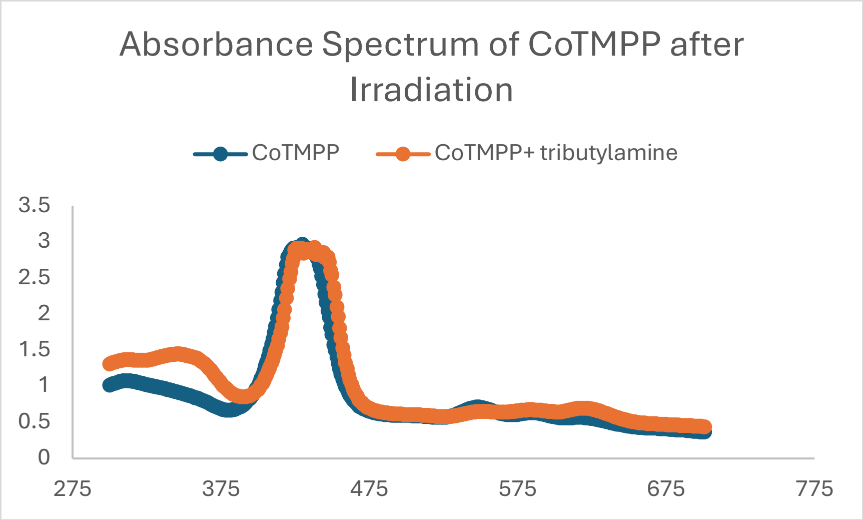

0.03 mM Co^{II}TMPP with or w/o $N(Bu)_3$ (10 eqv.), 1:1 H_2O:MeCN

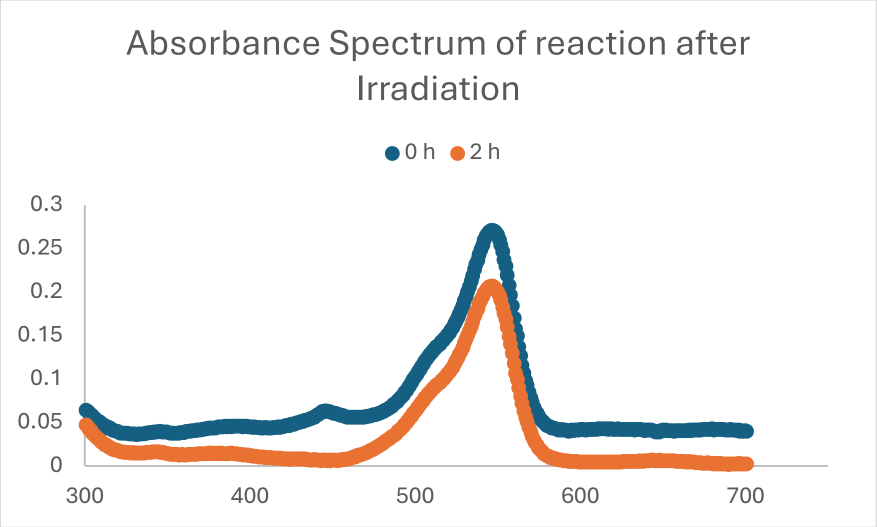

Reaction absorbance in 0 hr and after 2 hrs

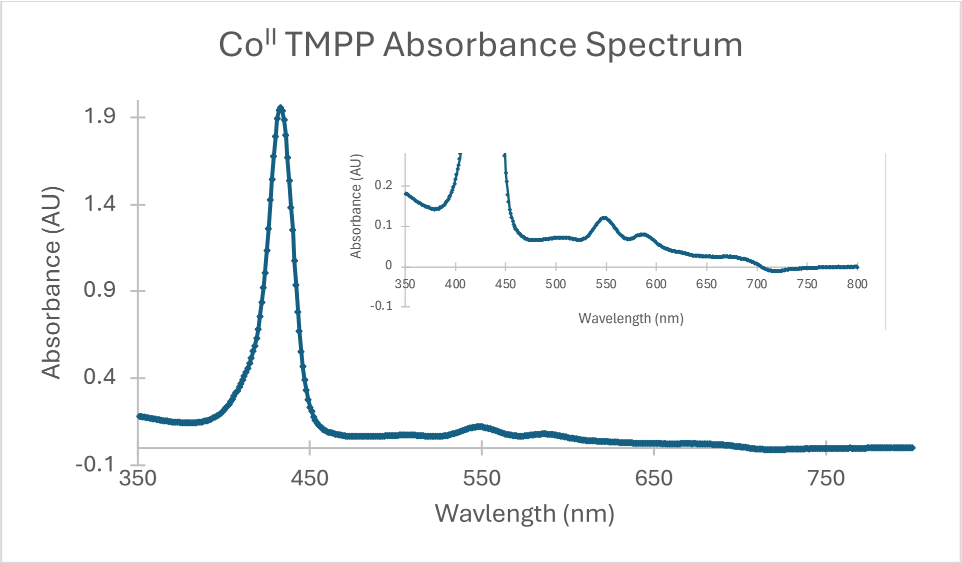

0.02 mM Co^{II}TMPP, 9:1 MeCN:H_2O.

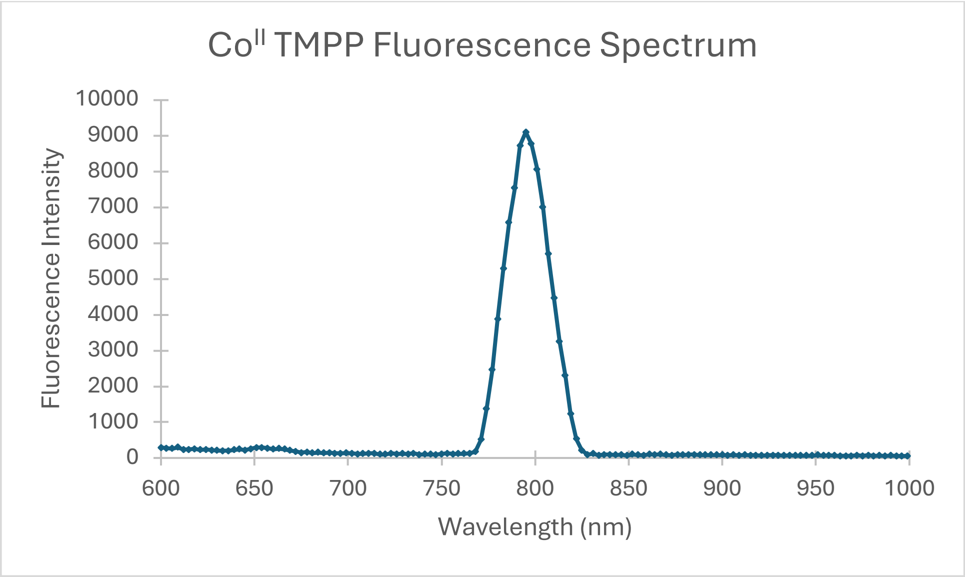

0.1 mM Co^{II}TMPP 9:1 MeCN:H_2O.

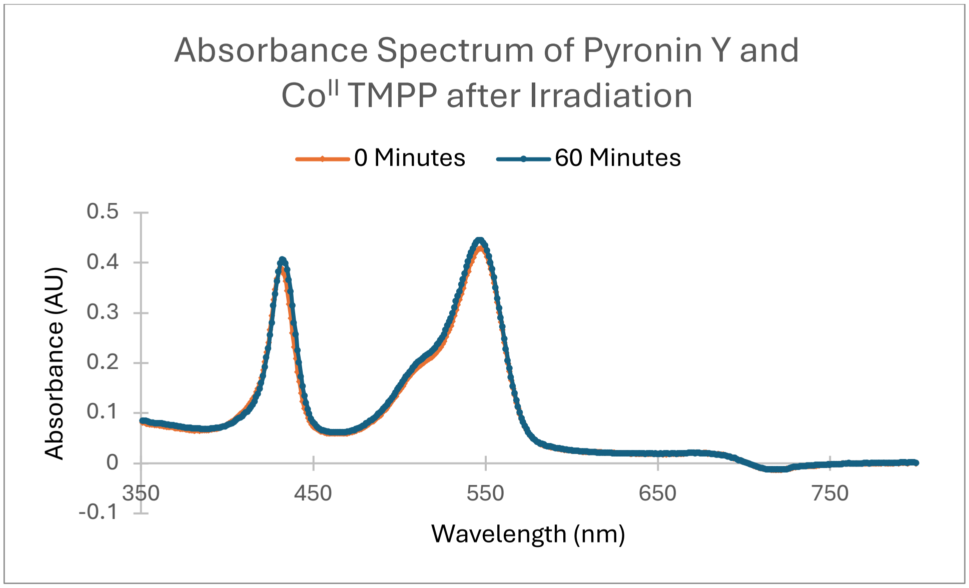

4 μM each Pyronin Y and Co^{II}TMPP, 9:1 MeCN:H_2O

a) Stern-Volmer Quenching Studies

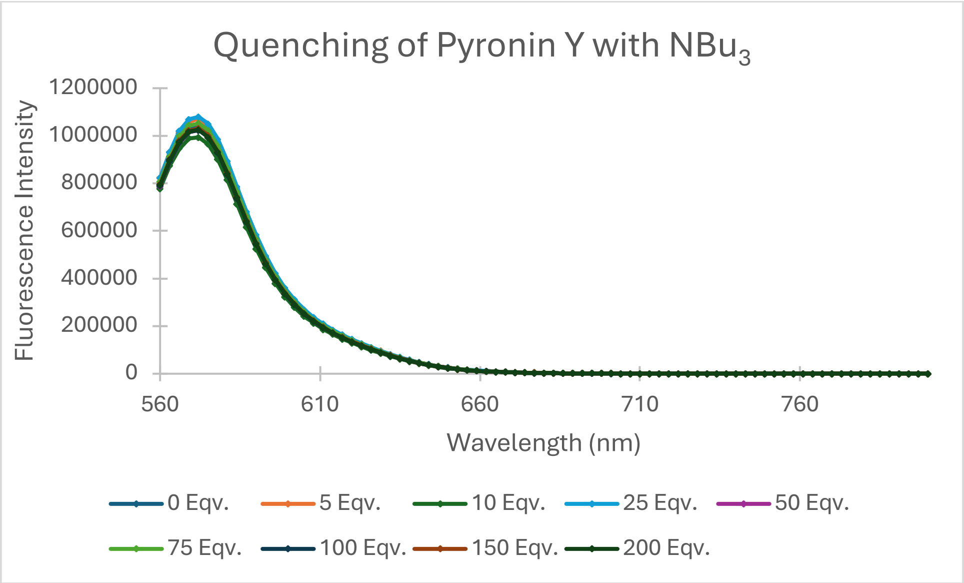

0.02 mM Pyronin Y, 9:1 MeCN:H_2O

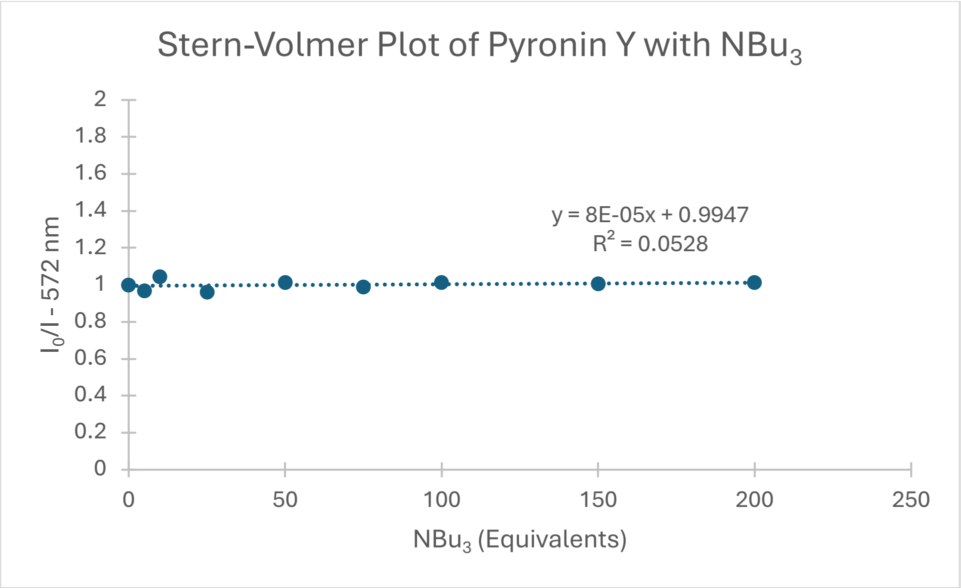

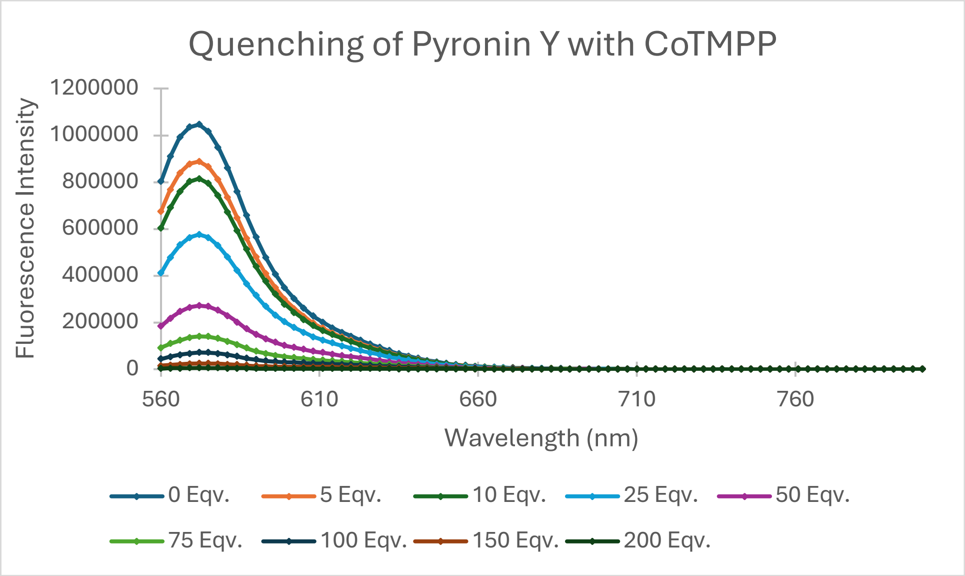

0.02 mM Pyronin Y, 9:1 MeCN:H_2O

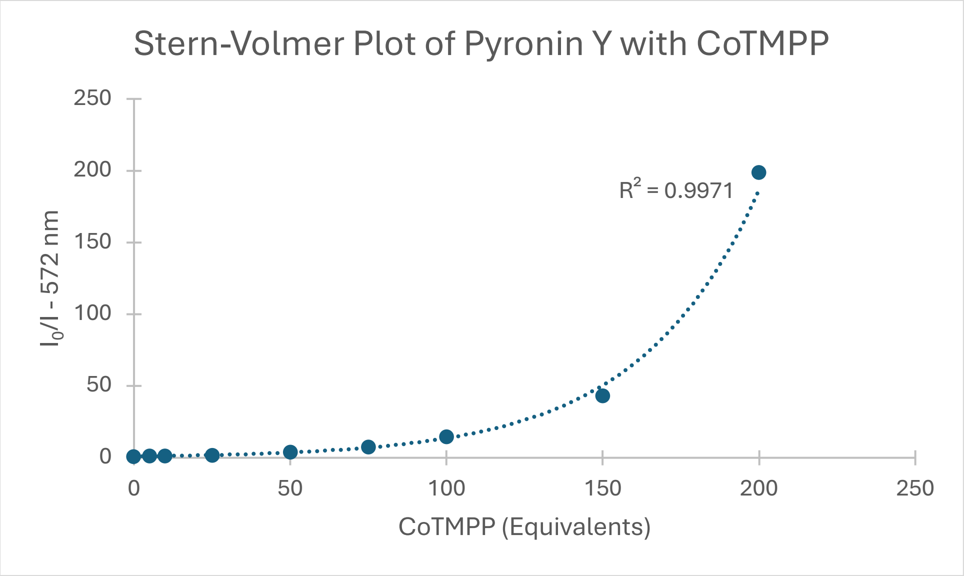

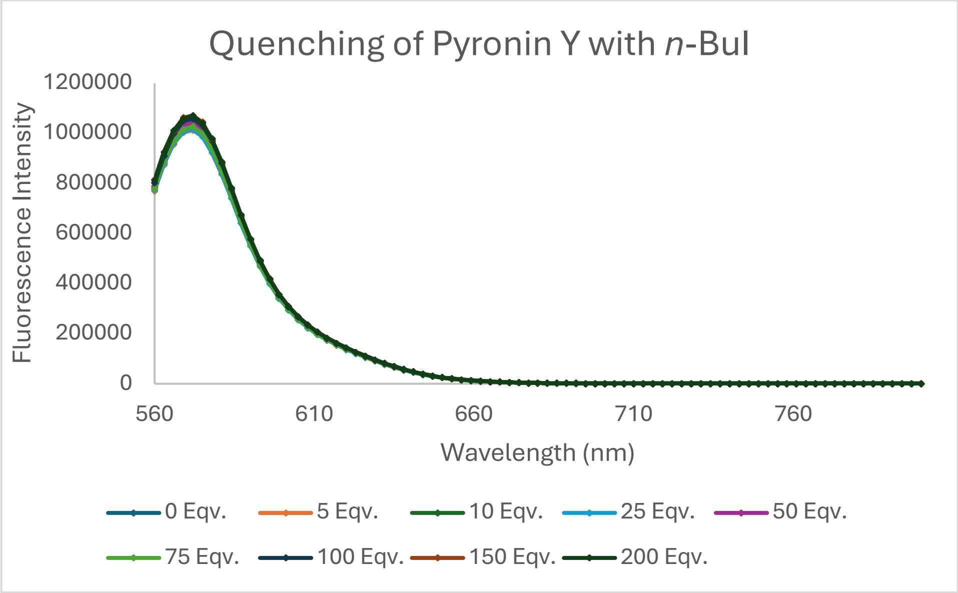

0.02 mM Pyronin Y, 9:1 MeCN:H_2O

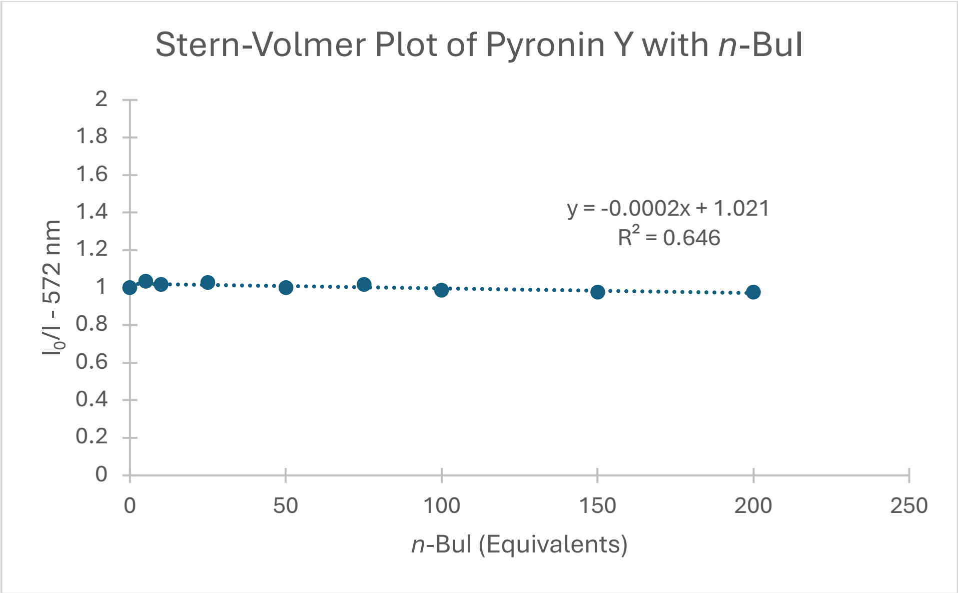

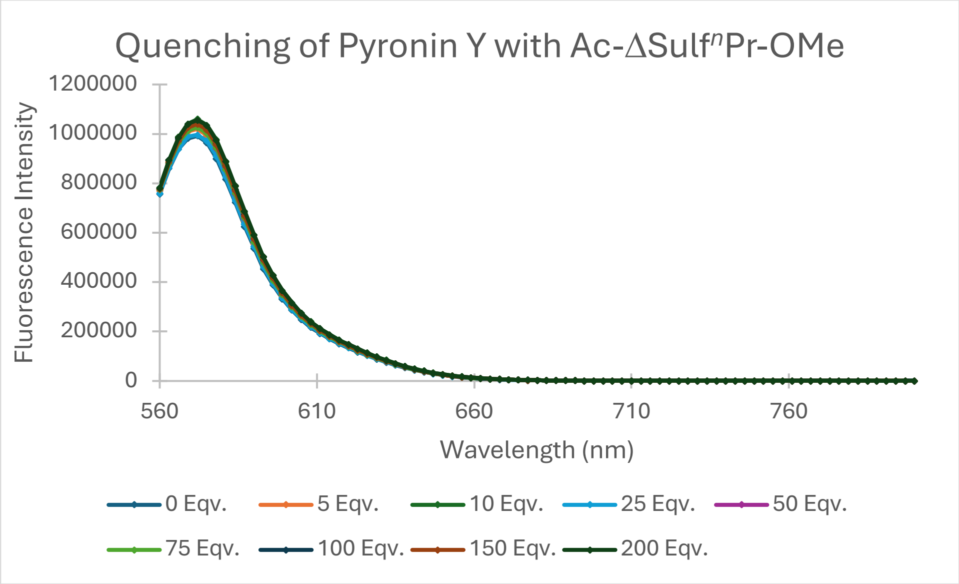

0.02 mM Pyronin Y, 9:1 MeCN:H_2O

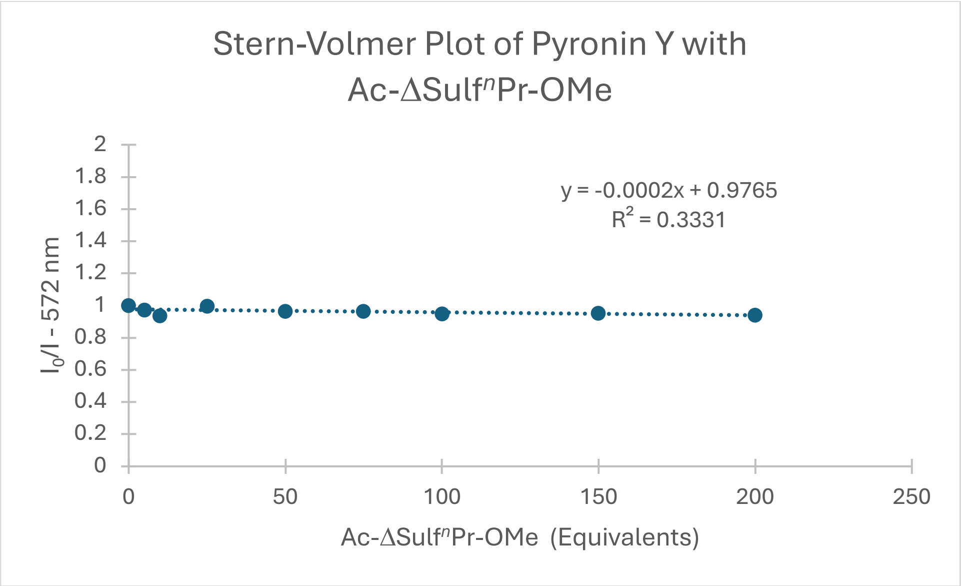

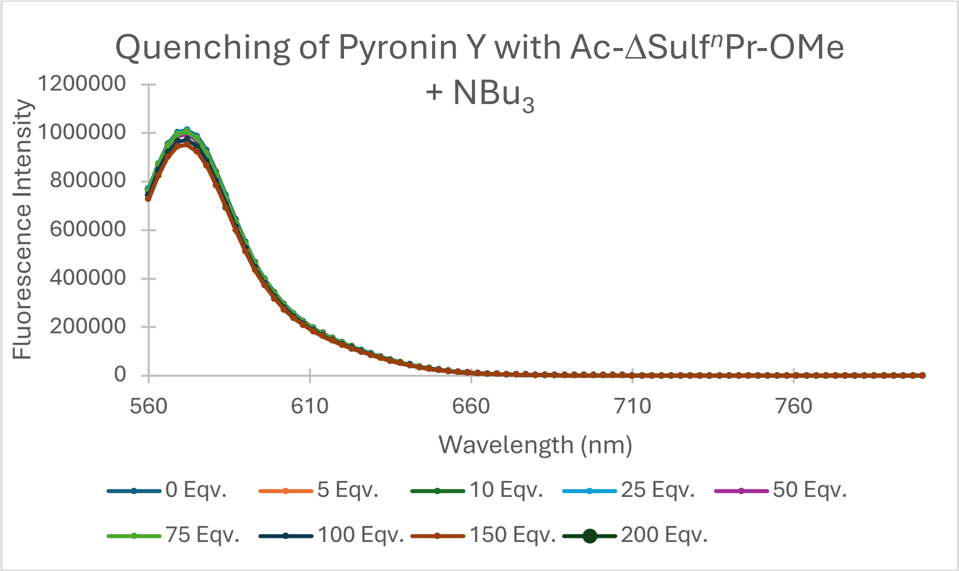

0.02 mM Pyronin Y, 9:1 MeCN:H_2O

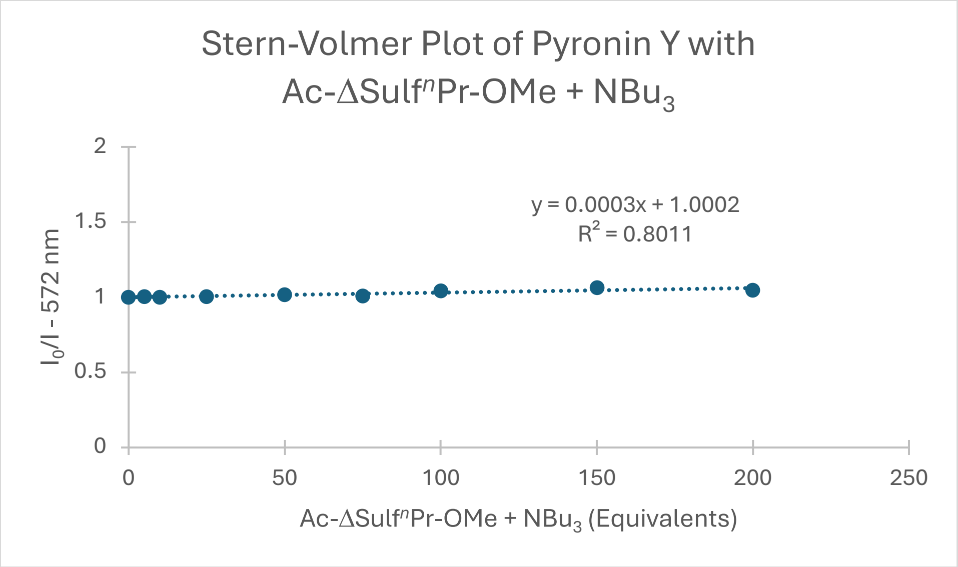

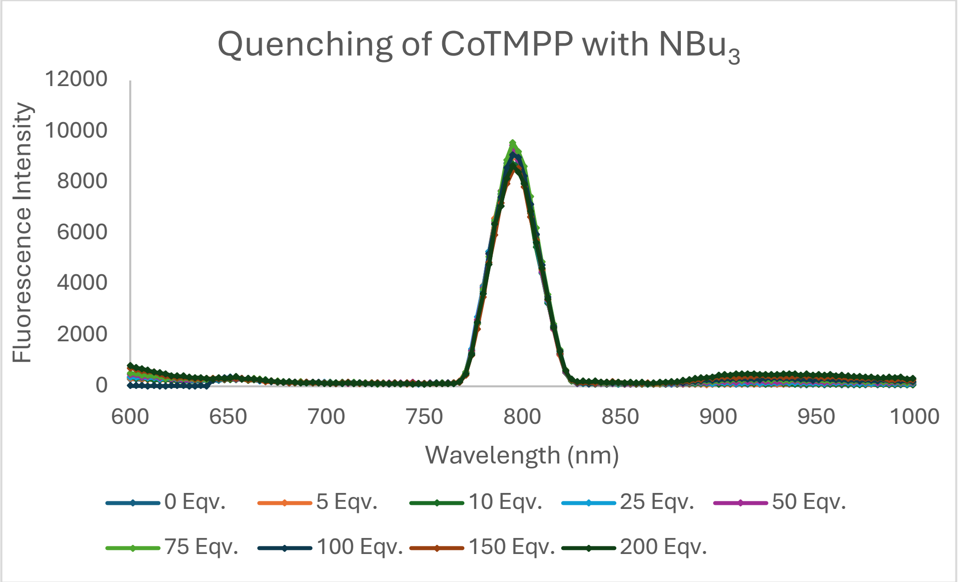

0.1 mM Co^{II}TMPP, 9:1 MeCN:H_2O

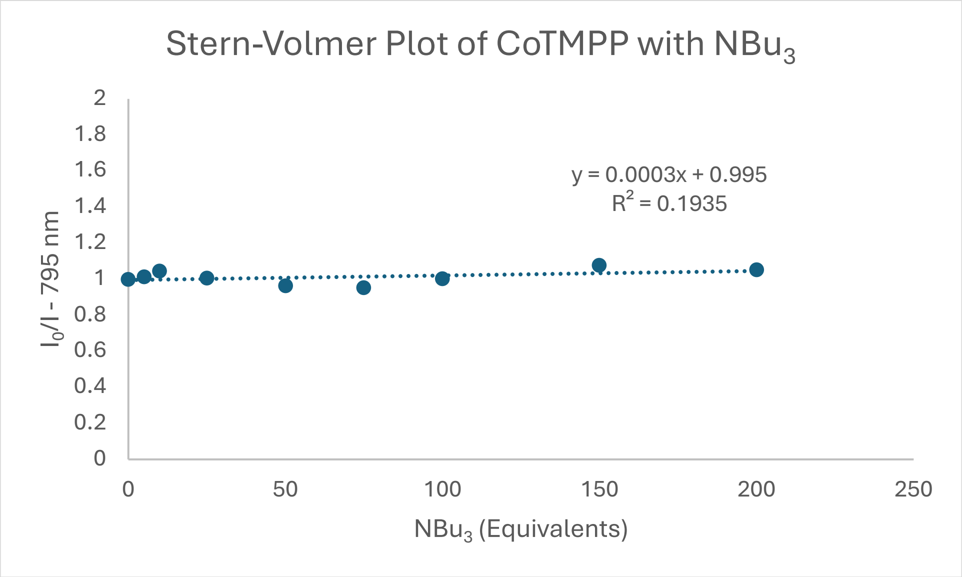

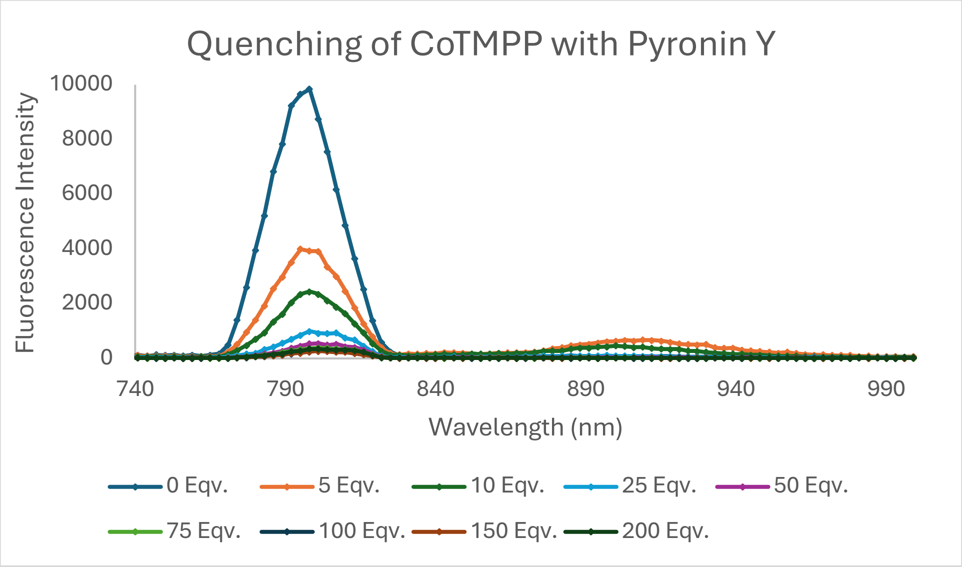

0.1 mM Co^{II}TMPP, 9:1 MeCN:H_2O

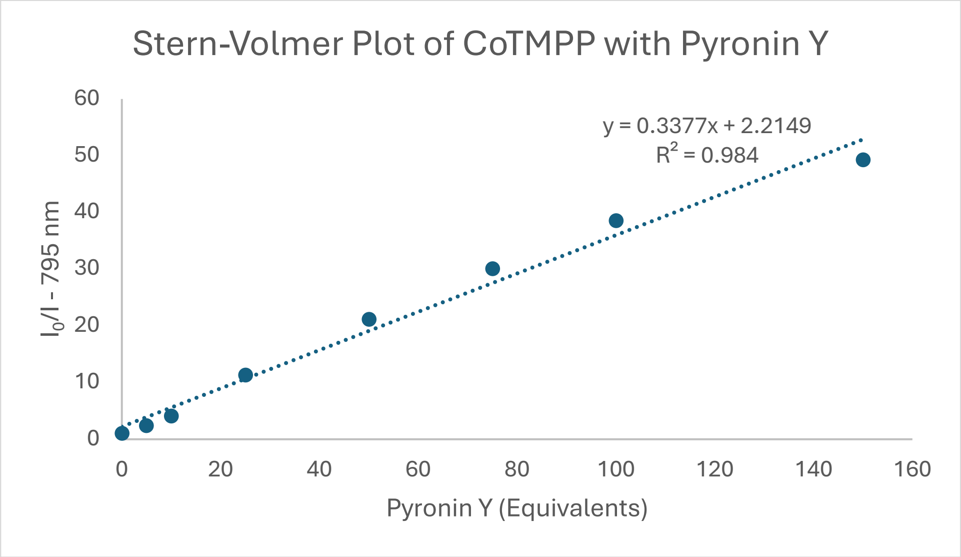

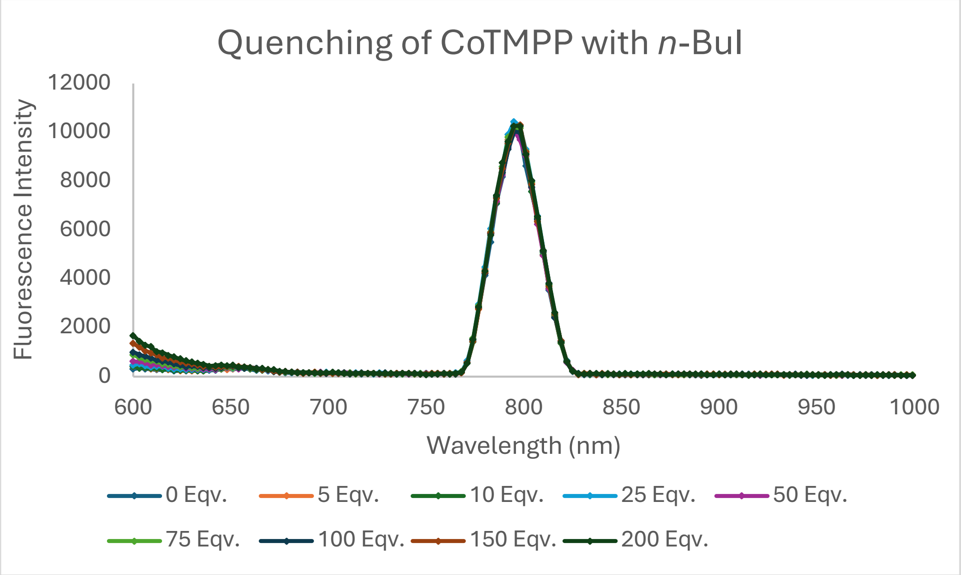

0.1 mM Co^{II}TMPP, 9:1 MeCN:H_2O

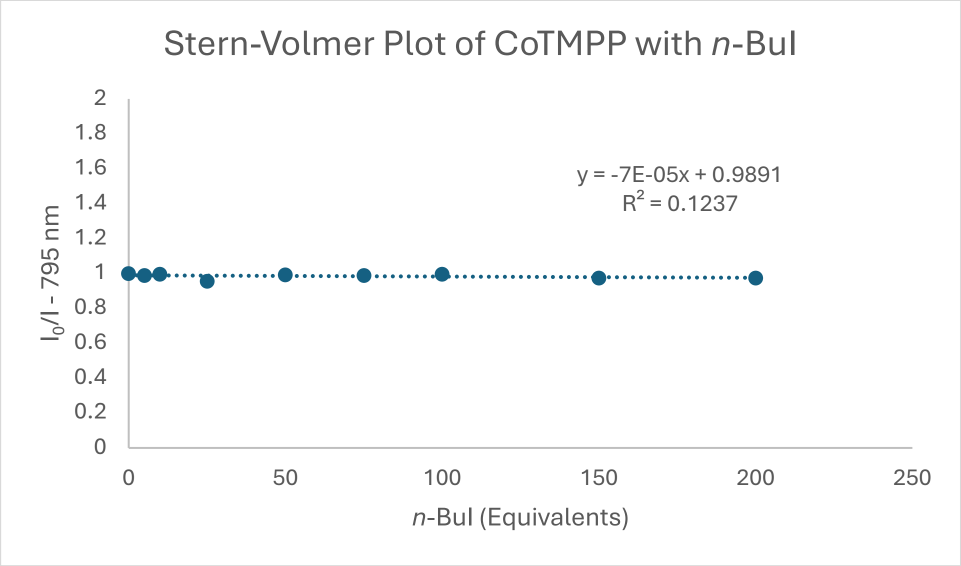

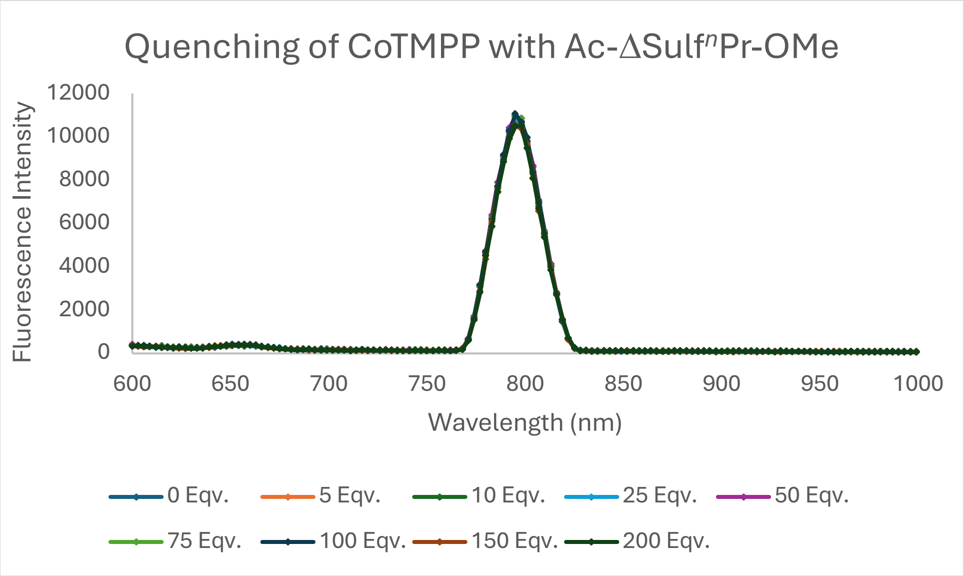

0.1 mM Co^{II}TMPP, 9:1 MeCN:H_2O

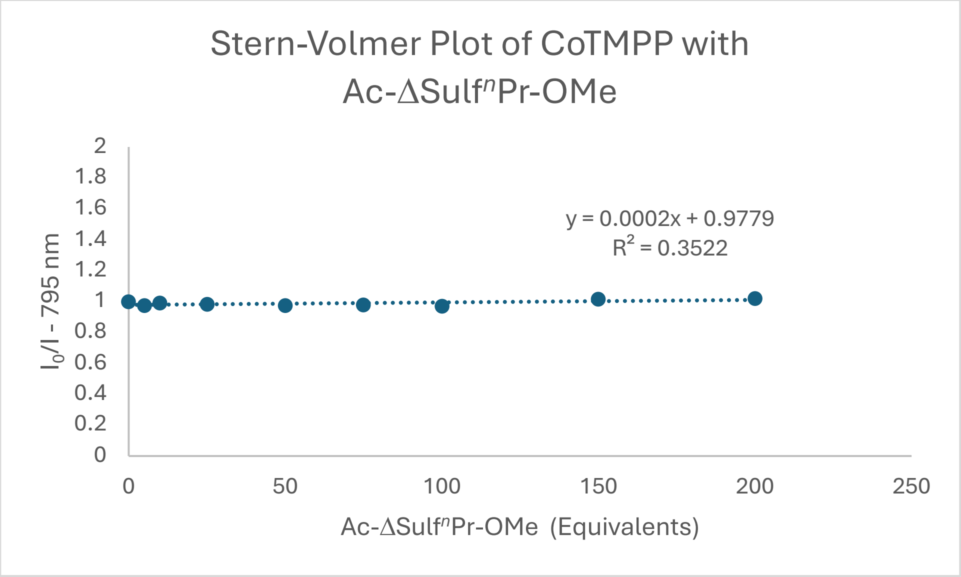

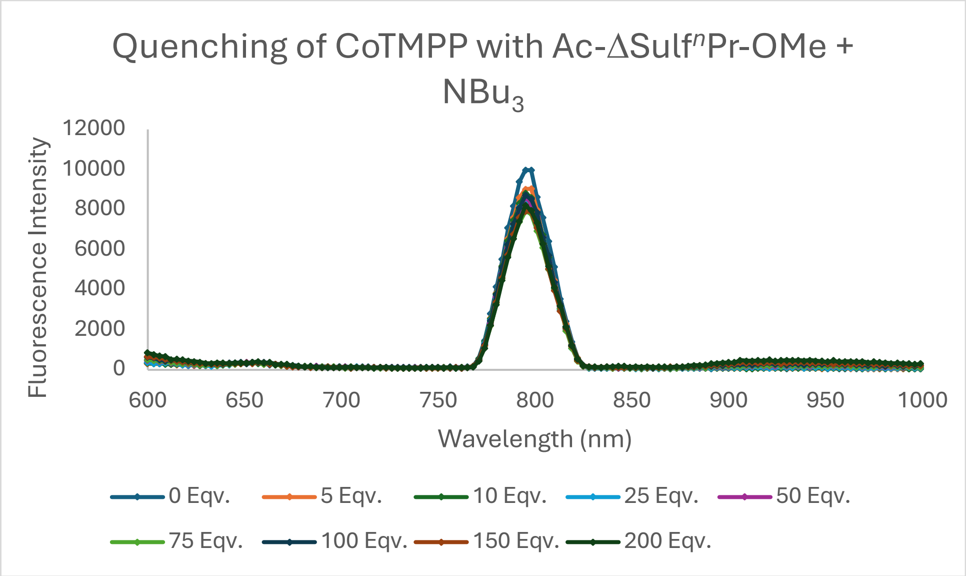

0.1 mM Co^{II}TMPP, 9:1 MeCN:H_2O

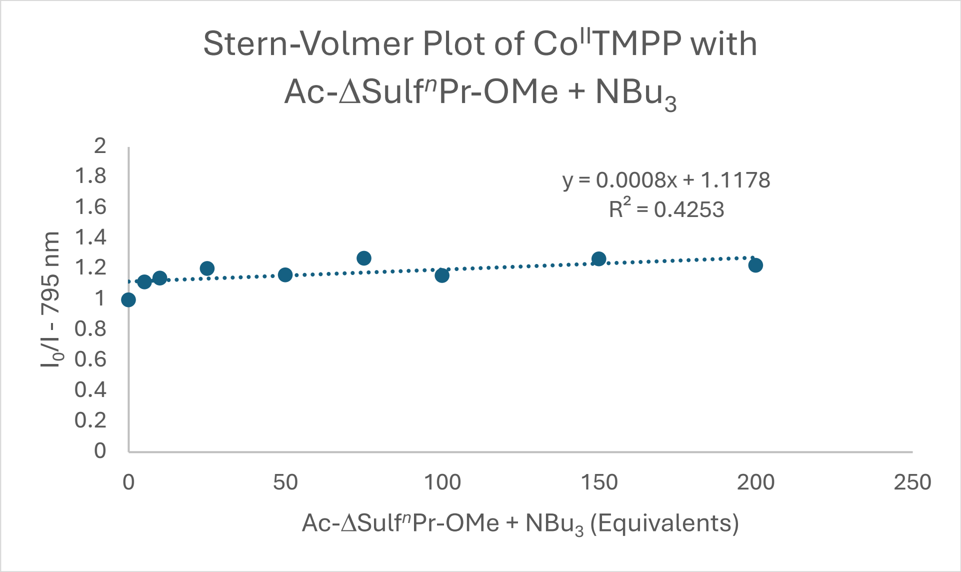

b) Cyclic Voltammetry

All cyclic voltammetry experiments were conducted using an IKA ElectraSyn 2.0 using a glassy carbon working electrode, platinum counter electrode, and silver wire reference electrode (3M aq. KCl). All spectra were obtained in N_2-degassed 9:1 MeCN:H_2O with 0.1 M $LiClO_4$ as supporting electrolyte. For pyronin Y, separate solutions were prepared to measure the oxidation and reduction potentials. Scan rates for each CV are indicated below.

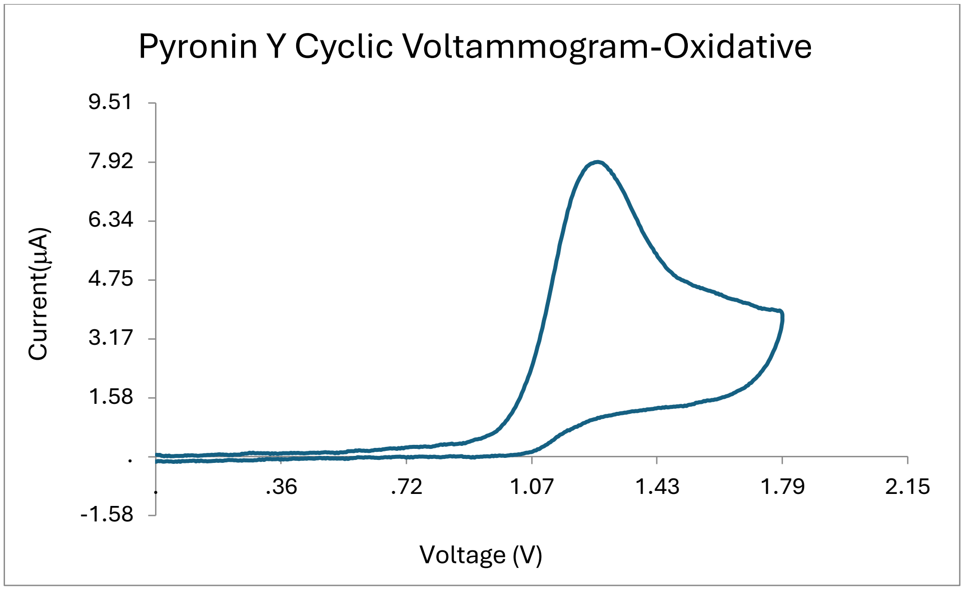

Scan Rate: 30 mV/s

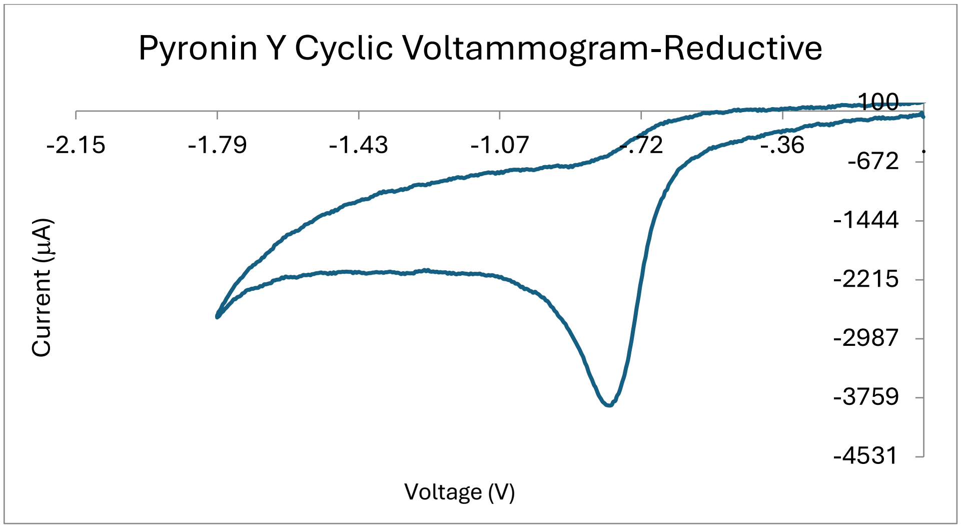

Scan Rate: 30 mV/s

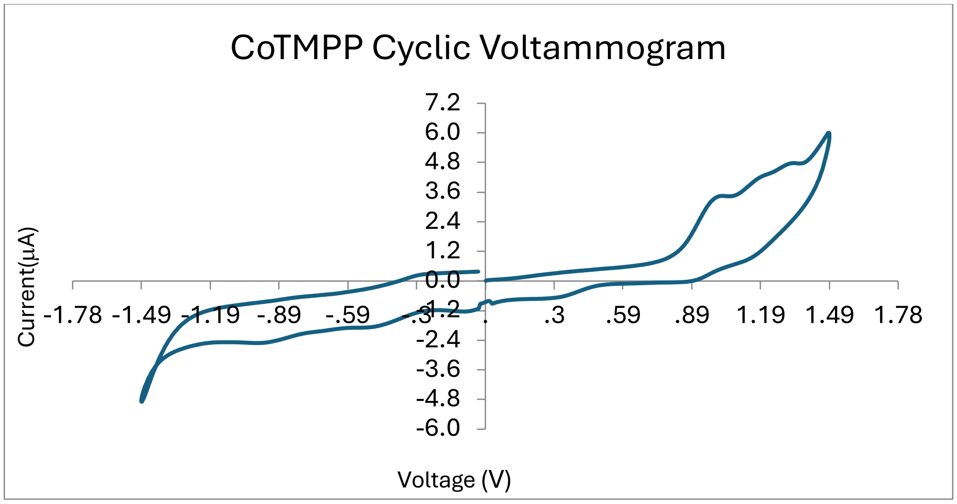

Scan Rate: 50 mV/s

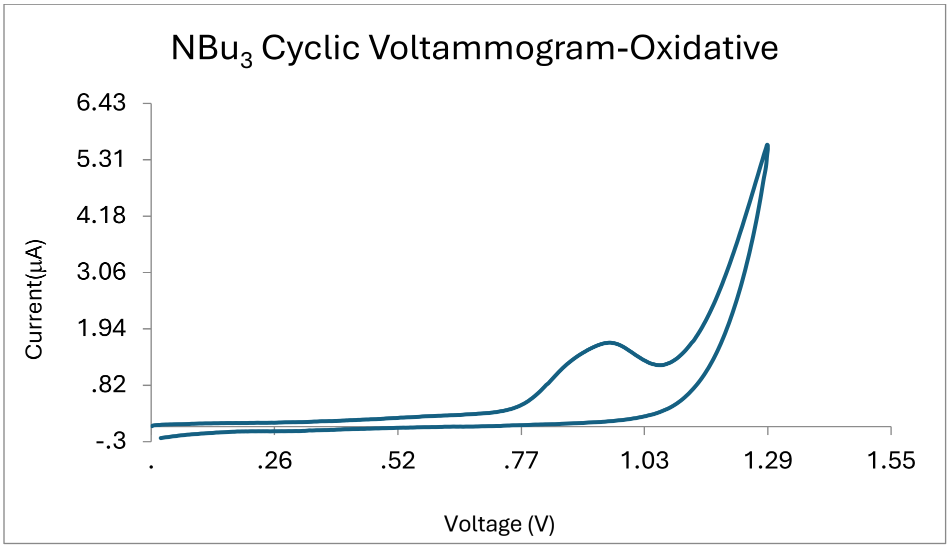

Scan Rate: 30 mV/s

G. Other Mechanistic Studies

Radical Clock Experiment

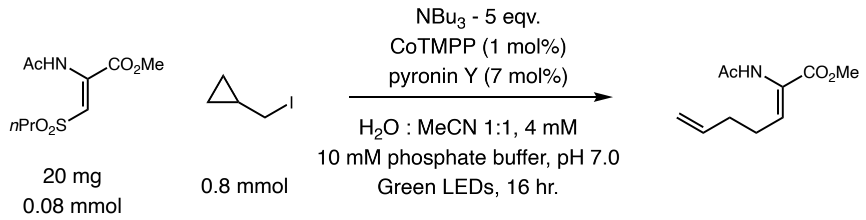

The radical clock experiment was performed following the "General Procedure for Alkylation of ΔSulf" with minor modifications. Ac-ΔSulf^{n}Pr-OMe (20 mg, 0.08 mmol), NBu_3 (0.40 mmol, 95.3 μL), pyronin Y (5.6 μmol, 1.7 mg), and CoTMPP (0.8 μmol, 0.6 mg) were added to a 50-mL round-bottom flask equipped with a magnetic stir bar. Water (8 mL), MeCN (10 mL), and 0.1 M phosphate buffer (2 mL) were next added, followed by 1-iodomethyl cyclopropane (0.8 mmol, 74.5 μL). The solution was capped with a septum, degassed for 10 minutes with N_2, sonicated for ~1 minute, and irradiated with 2 Kessil LEDs (525 nm) for 16 hours. At the completion of the reaction, the reaction mixture was concentrated to remove MeCN, transferred to a separatory funnel, and diluted with sat. aq. NH_4Cl (50 mL). The aqueous solution was then extracted with ethyl acetate (4 x 30 mL). The combined organic fractions were dried over $MgSO_4$, filtered, concentrated, and analyzed by crude ^1H NMR. Only the ring-opened product (shown) was observed. The crude reaction mixture was then purified by reverse-phase flash chromatography (C18; 0.1% formic acid/H_2O:0.1% formic acid/MeCN). Yield: 6.2 mg (39%).

^1H NMR (400 MHz, $CDCl_3$): δ 6.79 (1H, br. s), 6.69 (1H, t, *J* = 6.7 Hz), 5.86-5.75 (1H, m), 5.10-4.98 (2H, m), 3.78 (3H, s), 2.25 (4H, dt, *J* = 11.8, 6.4 Hz), 2.13 (3H, s).
Spectral data are consistent with literature.[4]

Synthesis of $Co^{III}TMPP(BF_4)$

$Co^{III}TMPP(BF_4)$ was synthesized following a slightly modified literature protocol (reported for $Co^{III}TMPP(SbF_6)$).[5] CoTMPP (100 mg, 0.126 mmol) was dissolved in CH_2Cl_2 (25 mL). In small portions, with rapid stirring, $AgBF_4$ (24.5 mg, 0.126 mmol) was added. The reaction was stirred at ambient temperature for 24 hours, then gravity-filtered over a pad of Celite and the solvent removed under reduced pressure. The solid was then redissolved in CH_2Cl_2 (~ 5 mL) and the filtration/evaporation step repeated three more times. The solid was then dried under high vacuum, affording a pink/purple solid (61 mg, 55%).

Calculation of Excited State Redox Potentials of Pyronin Y

The excited state redox potentials of pyronin Y were estimated using an established method disclosed by Nicewicz, utilizing the ground state redox potentials and the intersection of the absorbance and fluorescence spectra ($E_{0,0}$).[6]

Overlap = 565 nm = 2.194 eV

$$^*E_{red} = E_{0,0} + E^0_{red} = 2.19\text{ V} – 0.79\text{ V} = \mathbf{+1.40\text{ V}}$$

$$^*E_{ox} = E^0_{ox} – E_{0,0} = 1.27 – 2.19\text{ V} = \mathbf{-0.92\text{ V}}$$

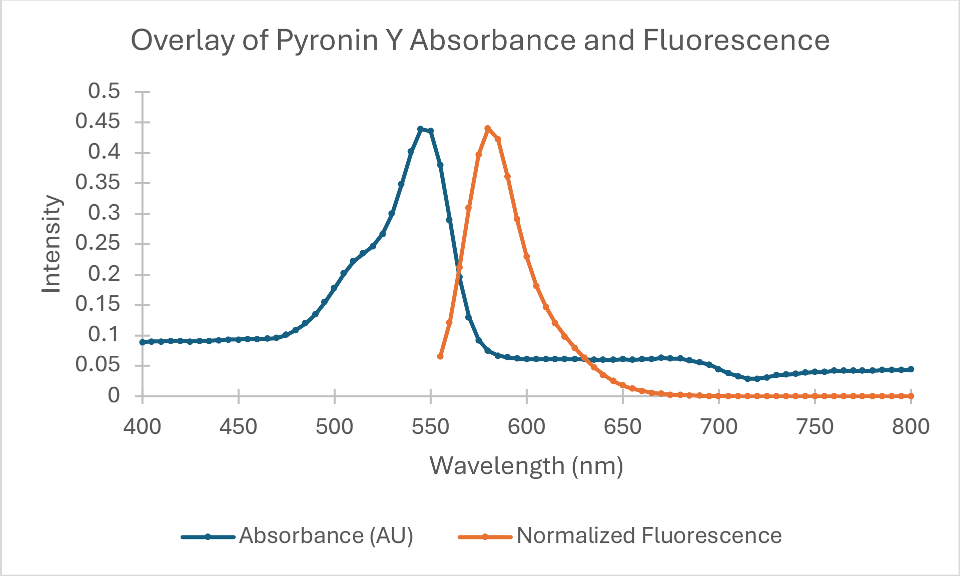

Role of tributylamine

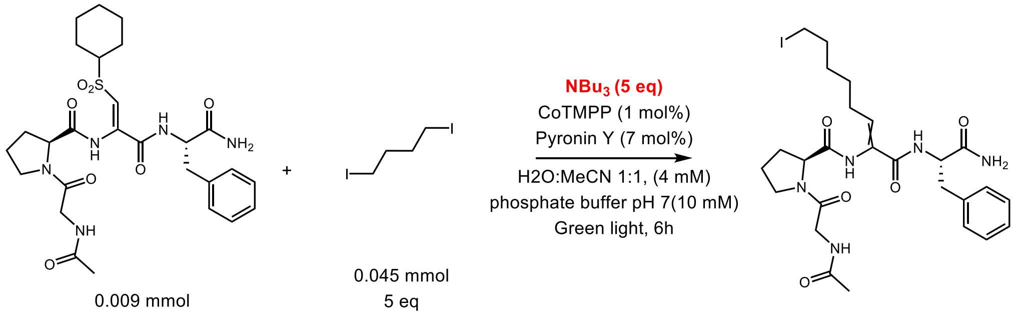

The reaction was performed following the "General Procedure for Alkylation of ΔSulf" with the modification of replacing tributylamine with DABCO and Pyridine.

Tributylamine replacement	Conversion
DABCO	0%, recover SM
Pyridine	0%, recover SM

LC-MS spectra

With DABCO

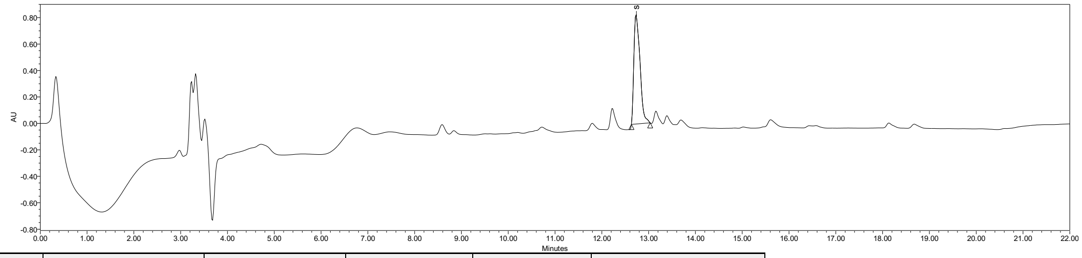

	Name	Retention Time	Area	% Area	Base Peak	Base Peak (Combined)
1	sm	12.734	7476699	100.00	576.47	576.46

With Pyridine

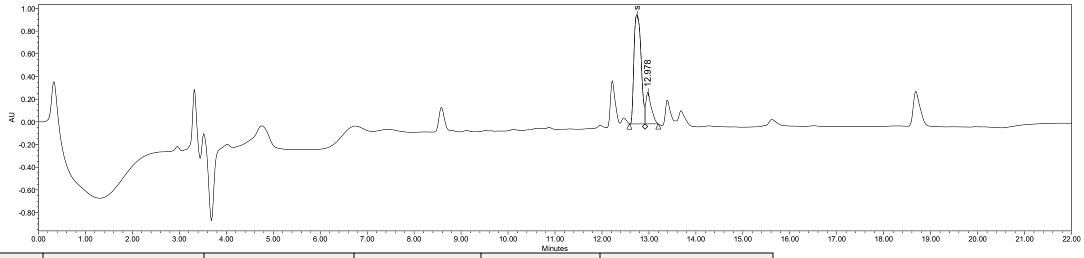

	Name	Retention Time	Area	% Area	Base Peak	Base Peak (Combined)
1	sm	12.744	10113383	81.80	576.47	576.46
2		12.978	2249423	18.20	702.45	702.46

H. NMR Spectra

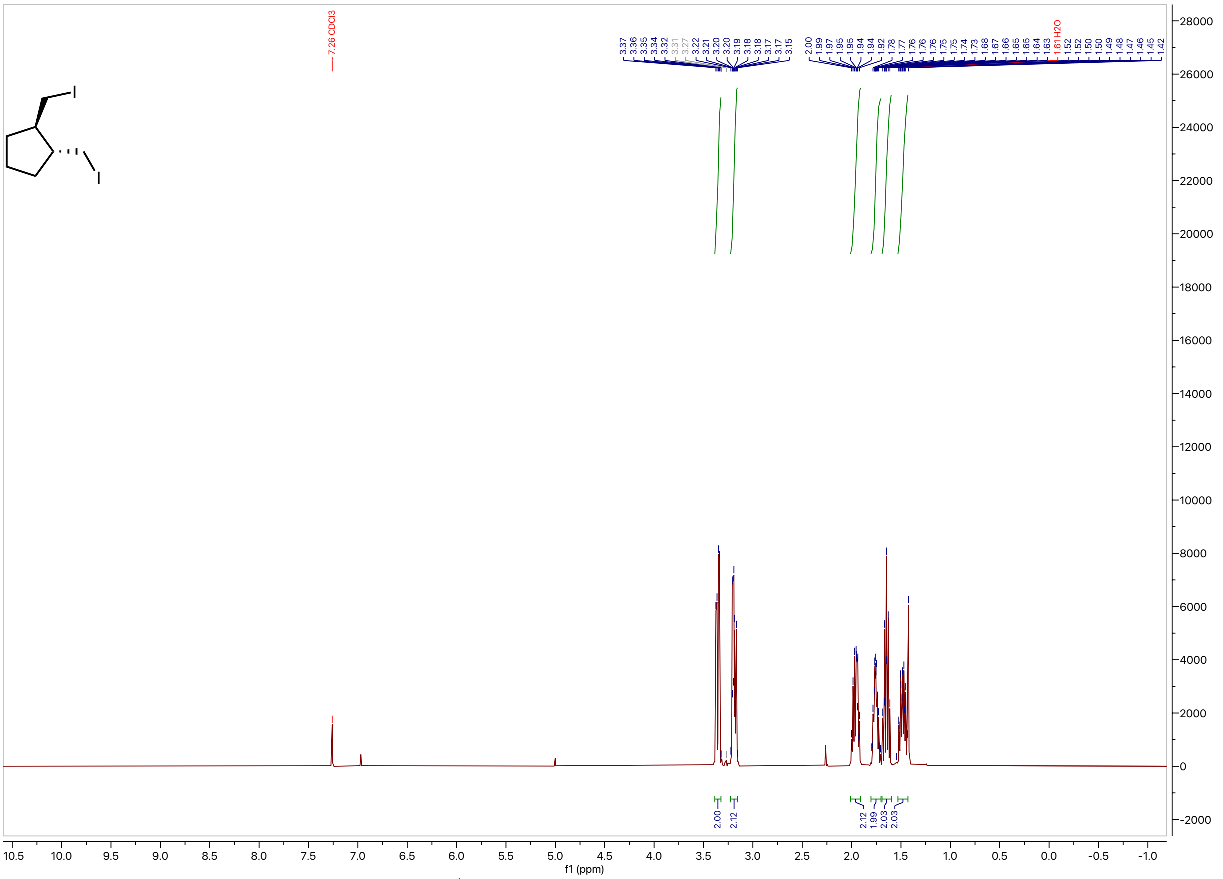

^{1}H NMR (400 MHz), $CDCl_3$

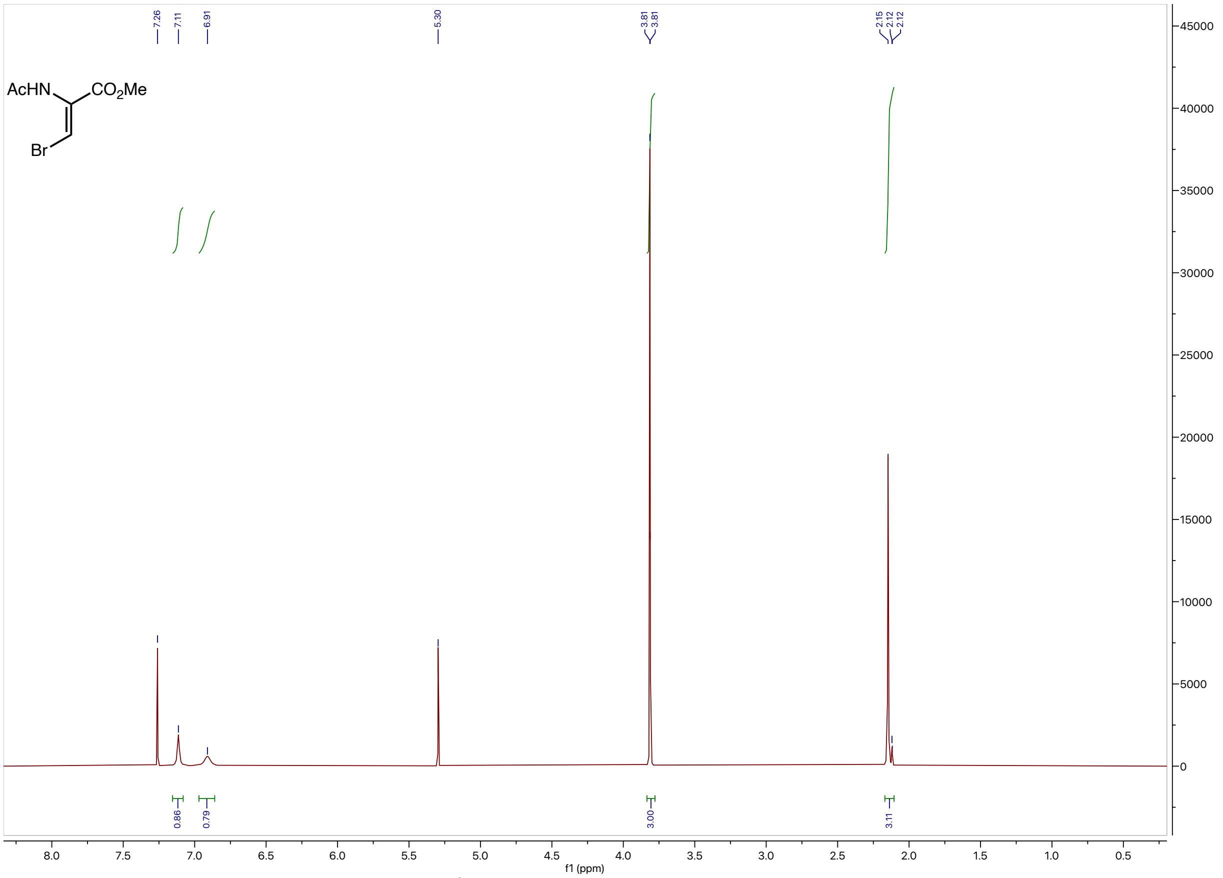

^1H NMR (400 MHz), $CDCl_3$

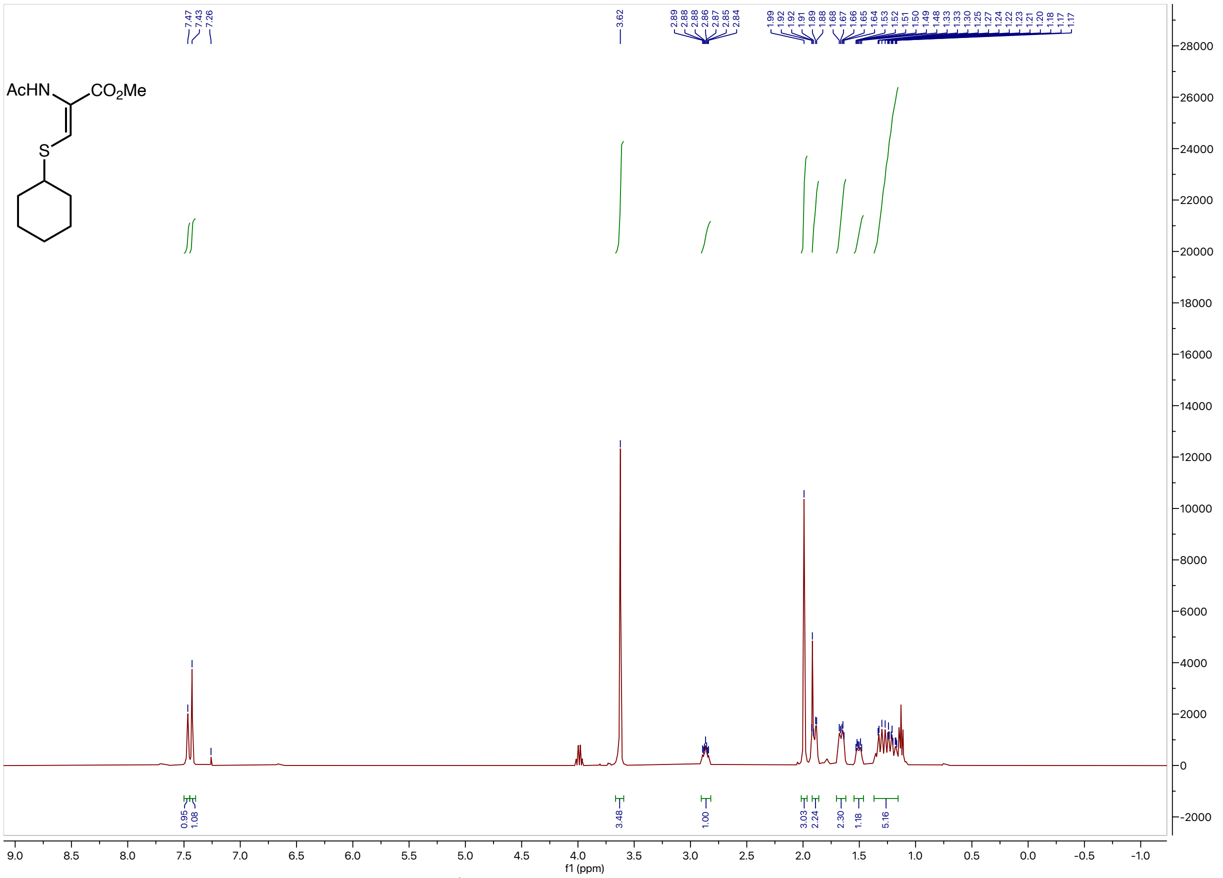

^1H NMR (400 MHz), $CDCl_3$

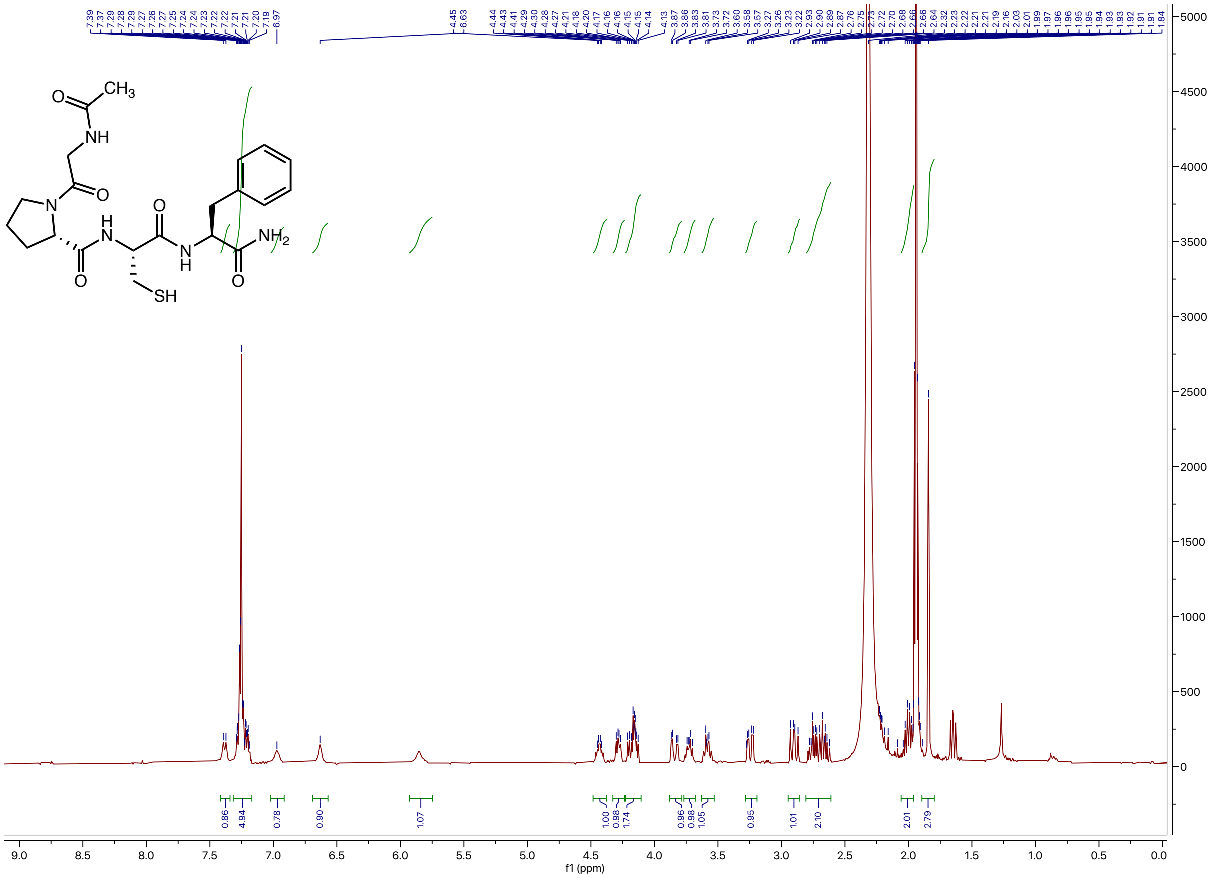

^{1}H NMR (400 MHz), CD_3CN

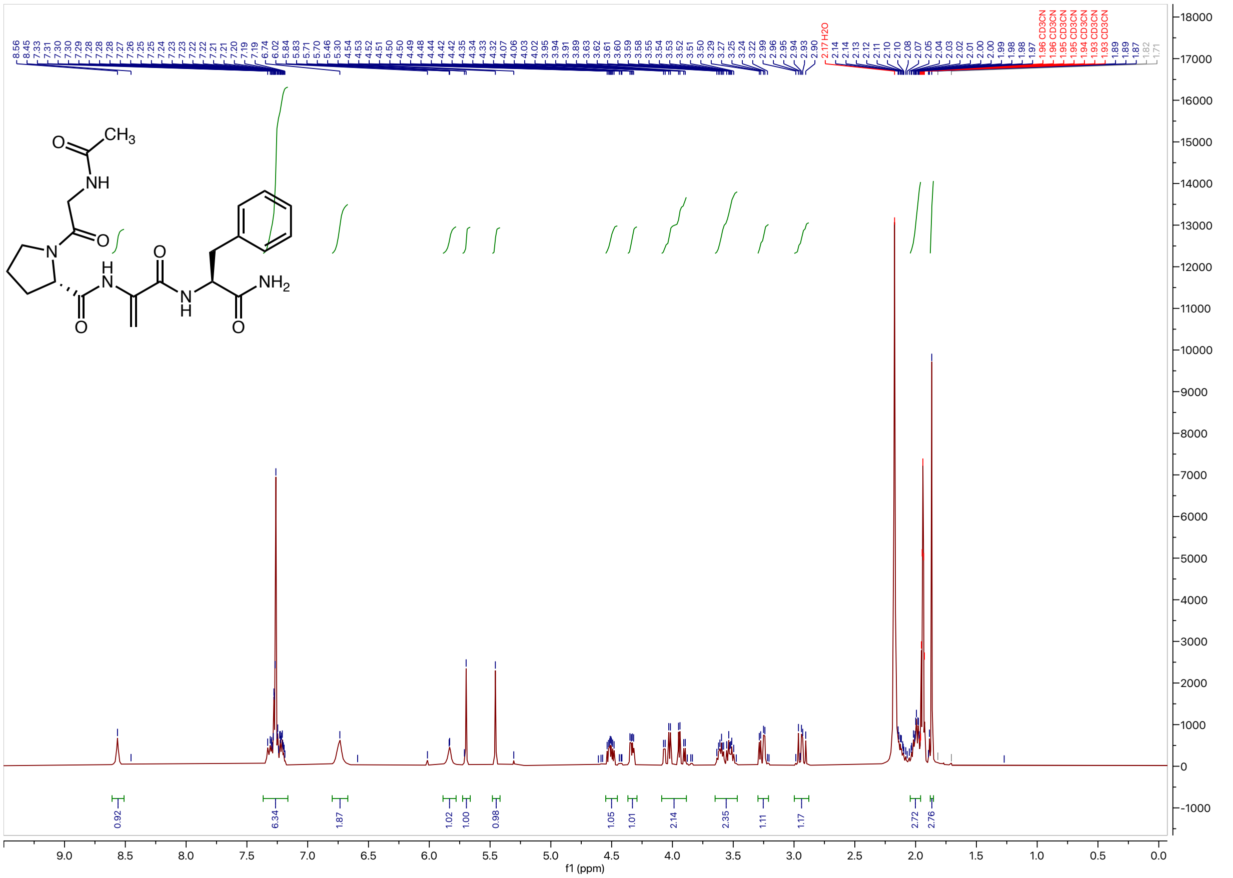

^1H NMR (400 MHz), CD_3CN

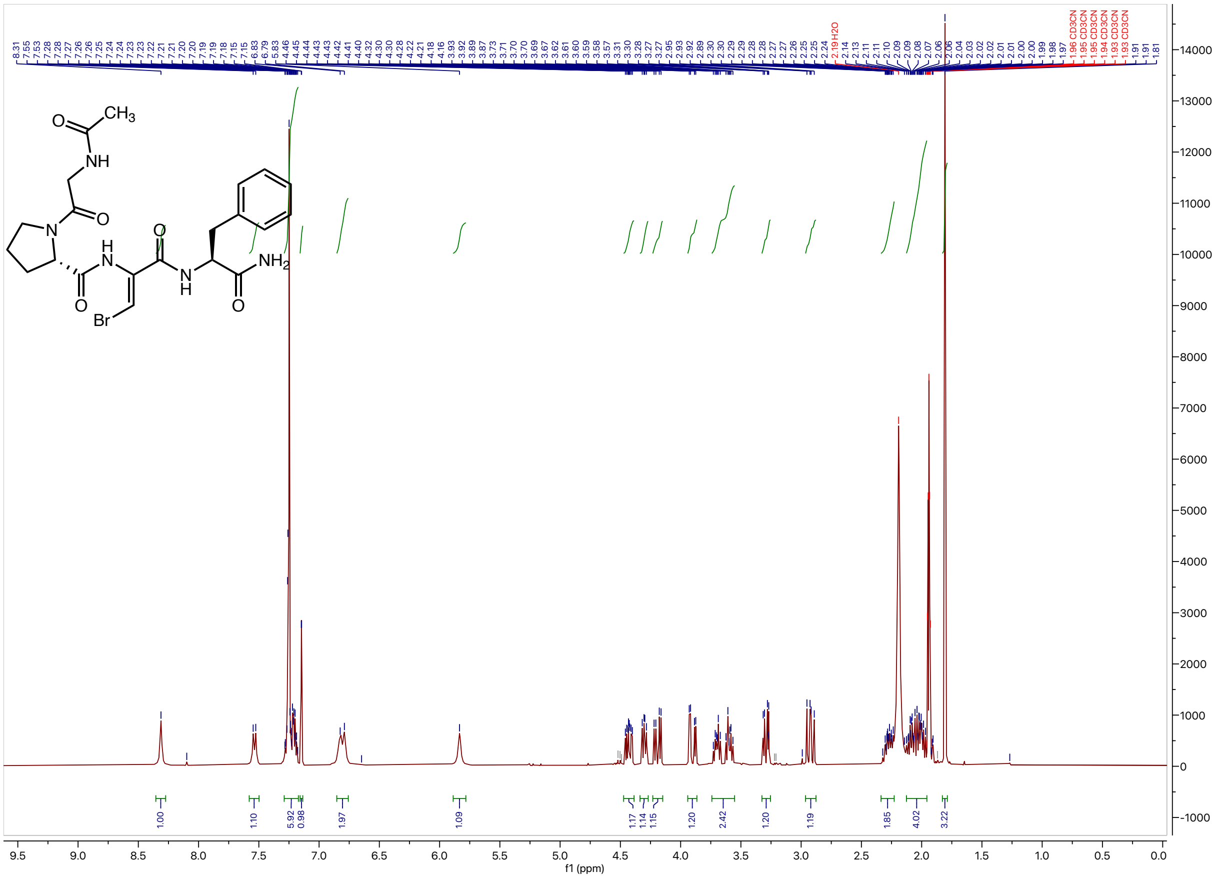

^1H NMR (400 MHz), CD_3CN

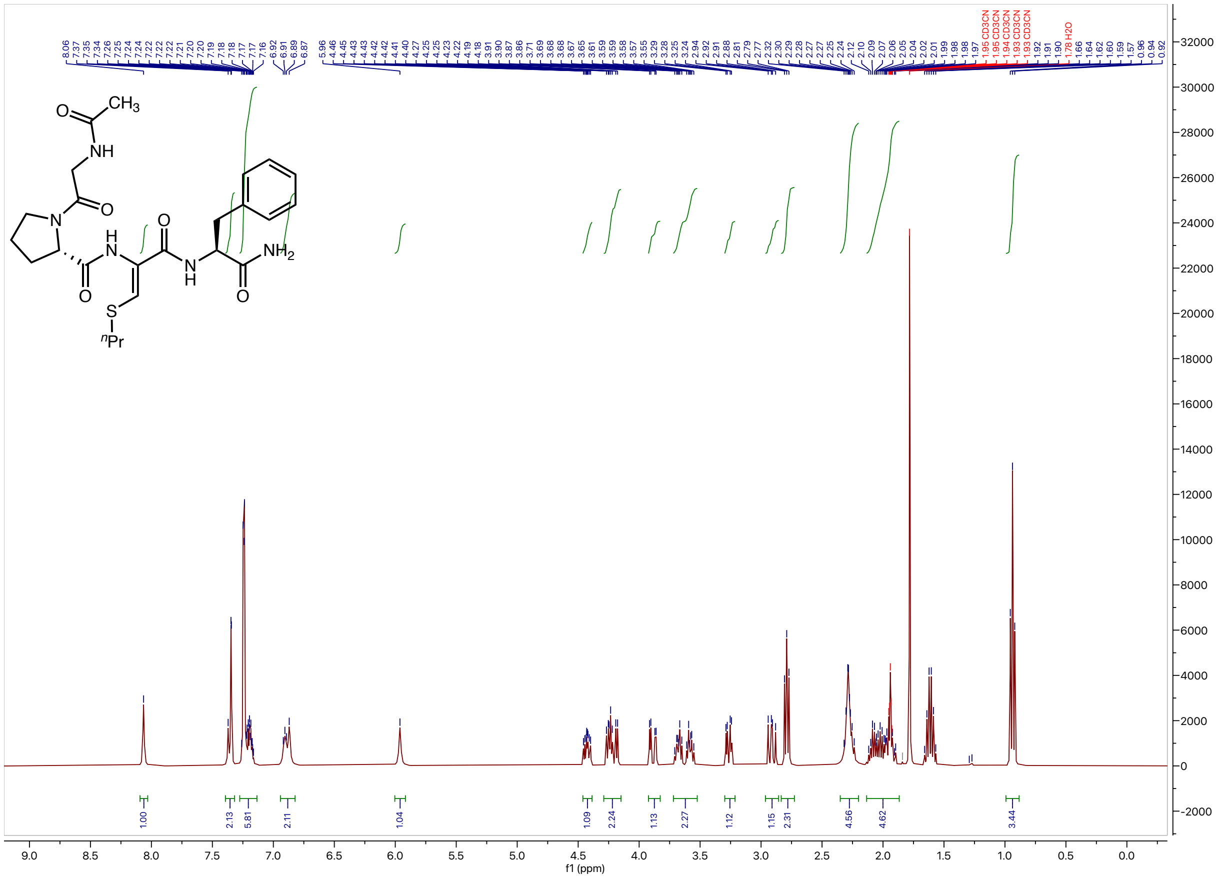

^{1}H NMR (400 MHz), CD_3CN

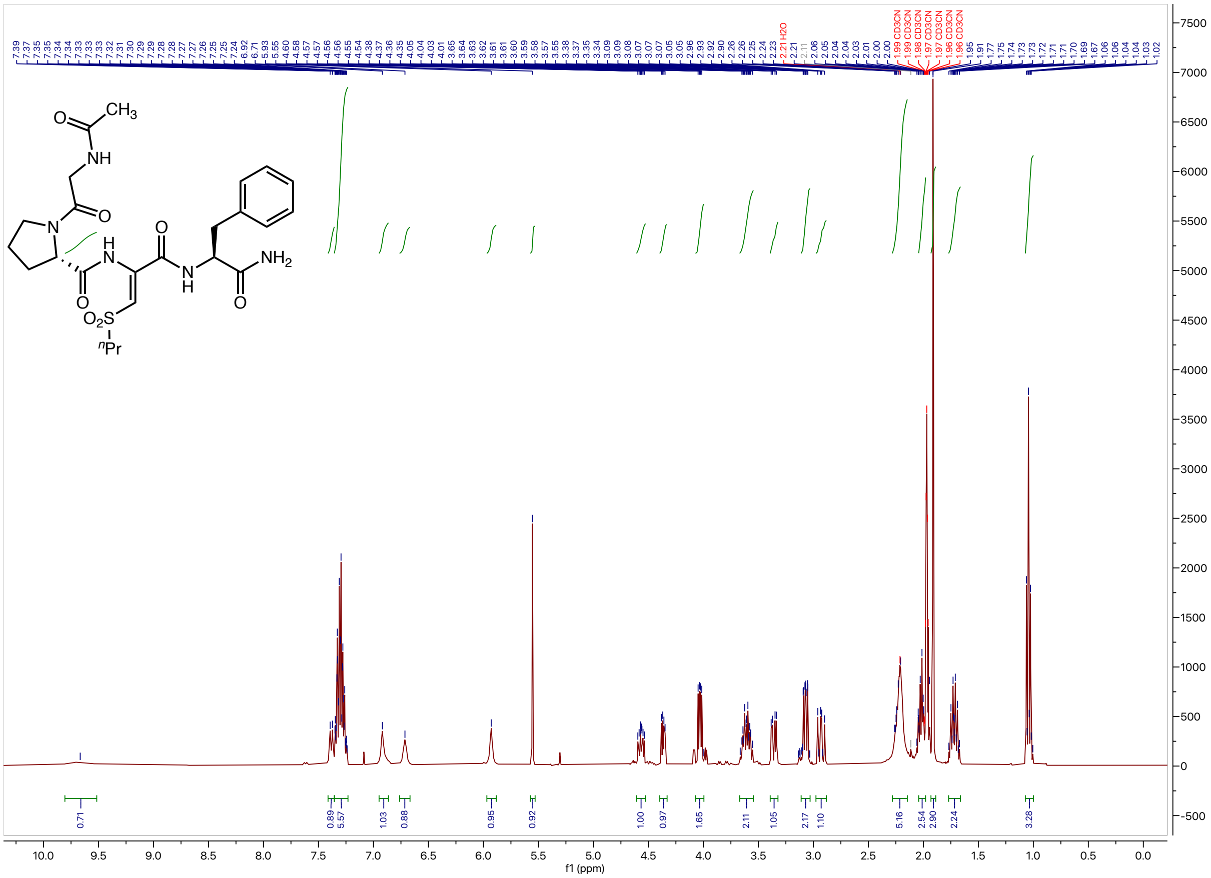

^1H NMR (400 MHz), CD_3CN

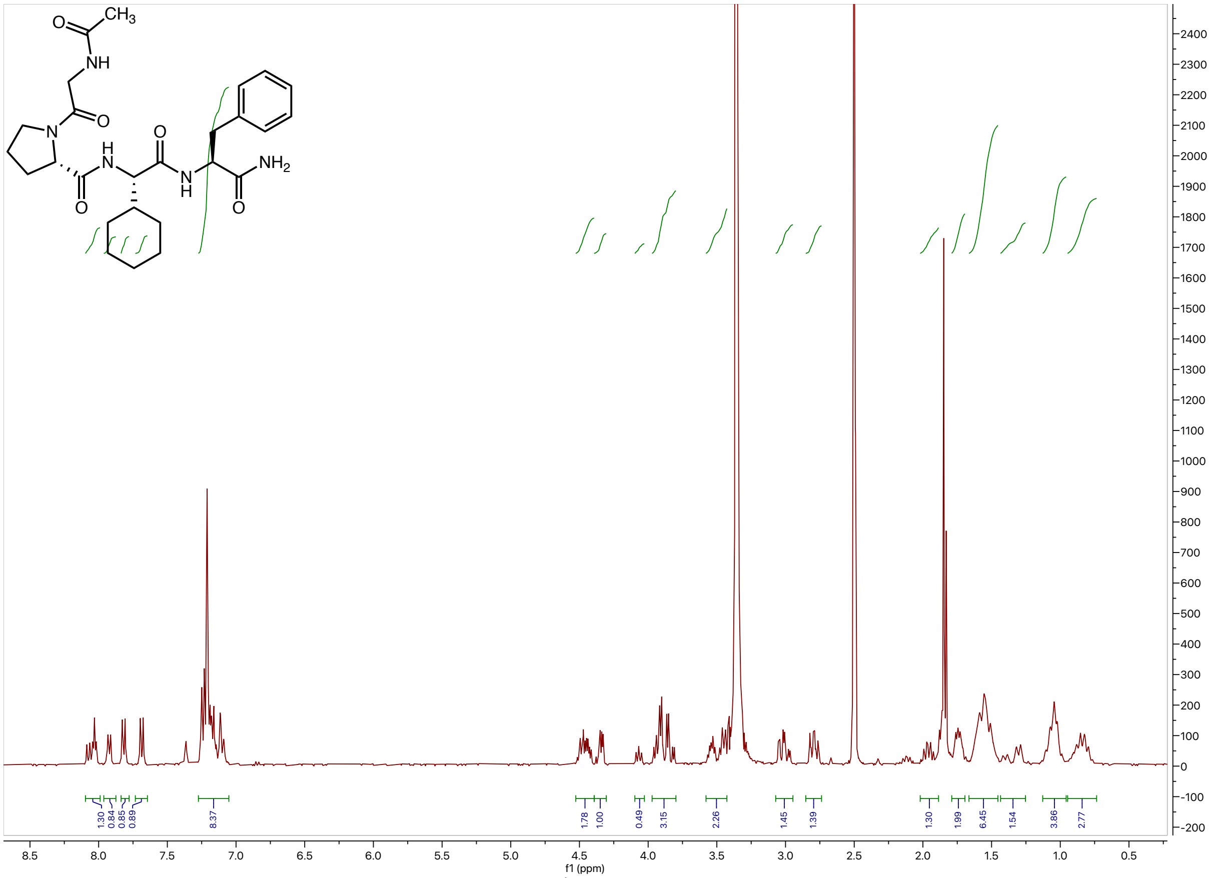

Authentic **(L)-3b**; ^{1}H NMR (400 MHz), CD_3OD

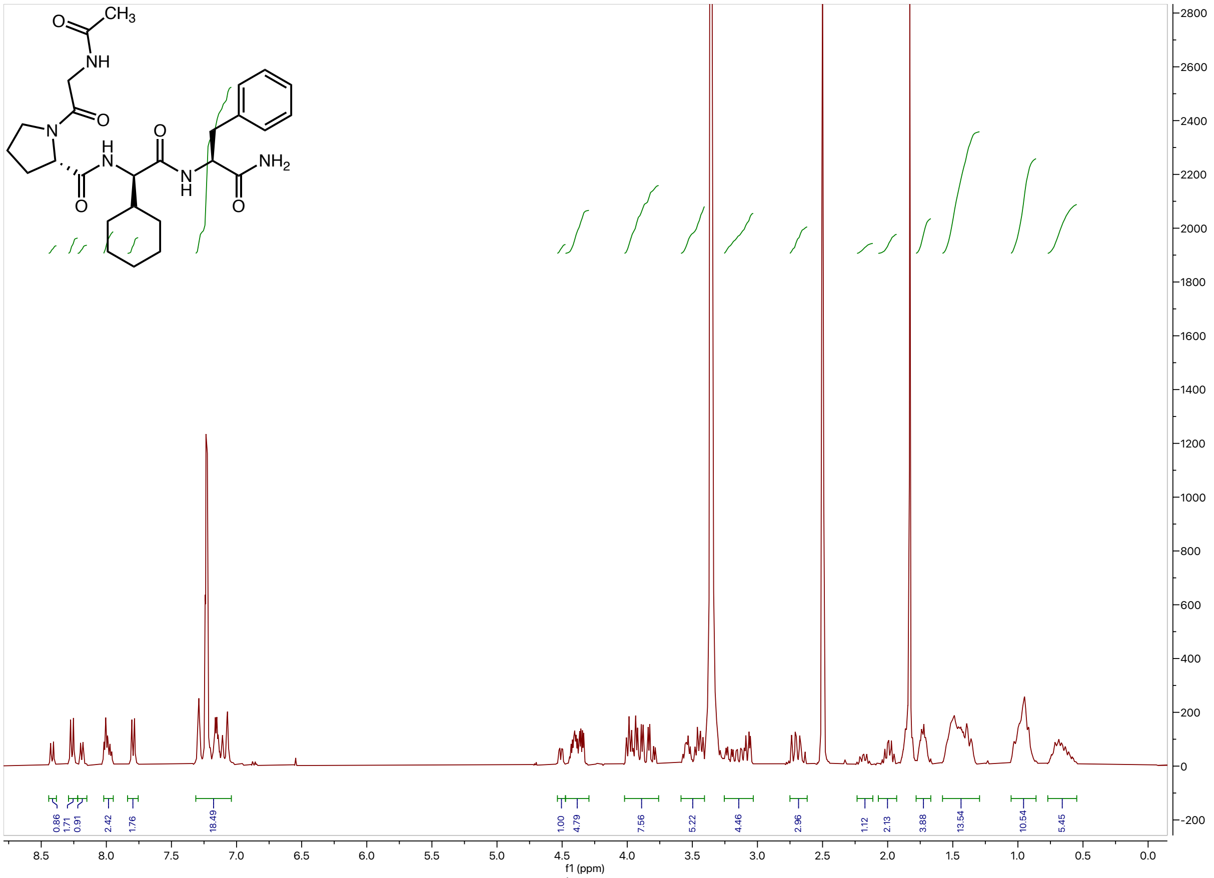

Authentic **(D)-3b**; ^{1}H NMR (400 MHz), CD_3OD

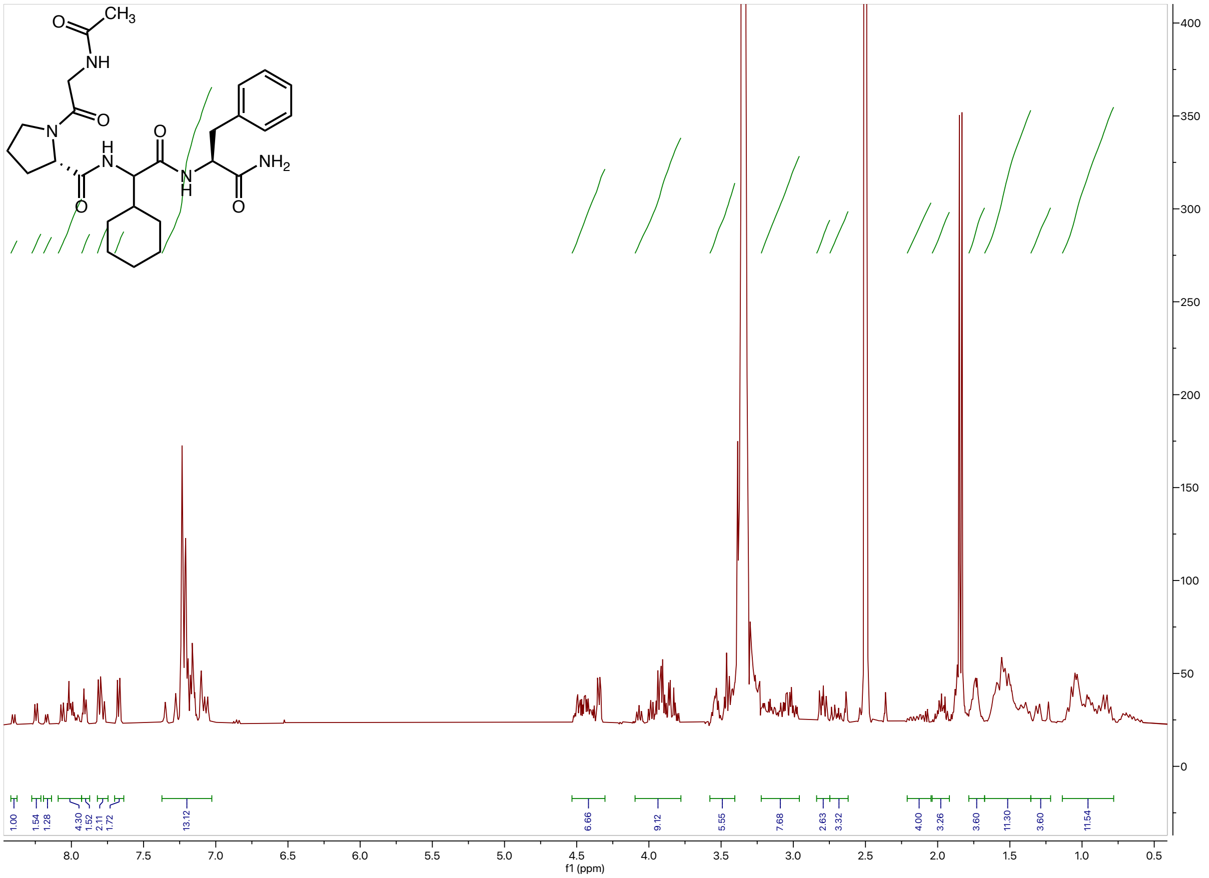

Isolated **(L/D)-3b**; ^{1}H NMR (500 MHz), CD_3OD

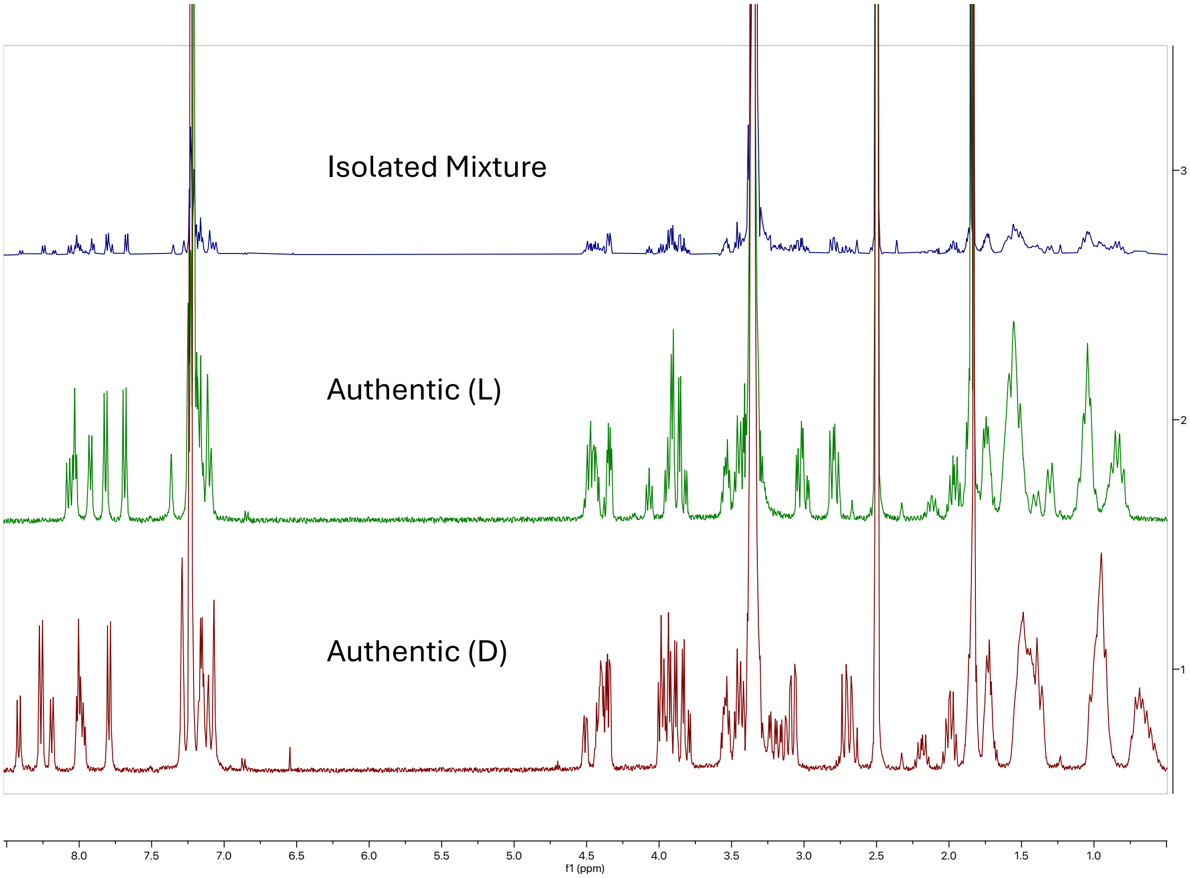

Overlay of Authentic **(L)- & (D)-3b** with an Isolated Mixture

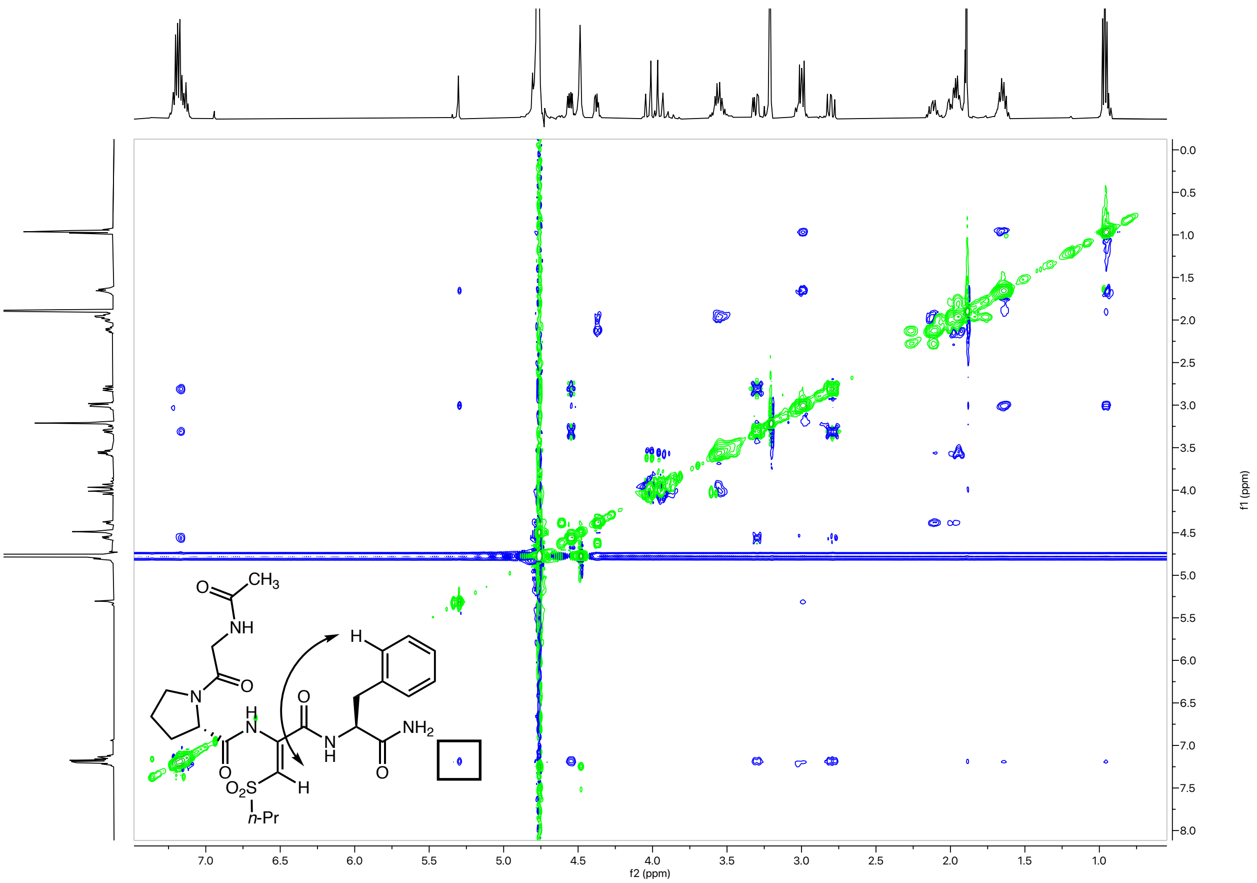

1b; (^{1}H, ^{1}H)-NOESY, CD_3OH

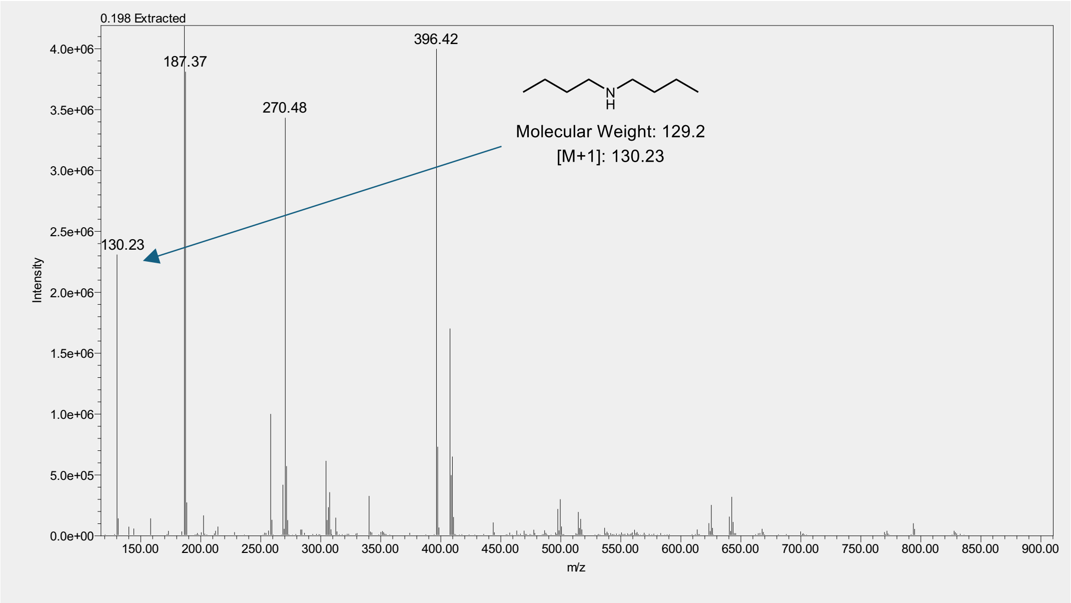

ESI+ spectra of crude reaction showing formation of NBu_3 hydrolysis products.

I. References

1) S. Gary, S. Bloom. "Peptide Carbocycles: From -SS- to -CC- via a Late-Stage "Snip-and-Stitch"." *ACS Cent. Sci.*, **2022**, *8*, 1537-1547.

2) J. Zhou, D. Sathe, J. Wang. "Understanding the Structure–Polymerization Thermodynamics Relationships of Fused-Ring Cyclooctenes for Developing Chemically Recyclable Polymers." *J. Am. Chem. Soc.* **2022**, *144,* 928-934.

3) K. Anjali, S. Nishimura. "Efficient Conversion of Furfural to Succinic Acid using Cobalt-Porphyrin Based Catalysts and Molecular Oxygen." *J. Catal.* **2023**, *428*, 115182.

4) K. Venkatasubbaiah, X. Zhu, E. Kays, K. I. Hardcastle, C. W. Jones. "Co(III)-Porphyrin-Mediated Highly Regioselective Ring-Opening of Terminal Epoxides with Alcohols and Phenols." *ACS Catal.* **2011**, *1*, 439-492.

5) E. Teoh, E. M. Campi, W. Roy Jackson, A. J. Robinson. "One-pot Tandem Enantioselective Hydrogenation-Hydroformylation Synthesis of Cyclic a-Amino Acids." *New J. Chem.* **2003**, *27*, 387-394.

6) A. R. White, L. Wang, D. A. Nicewicz. "Synthesis and Characterization of Acridinium Dyes for Photoredox Catalysis." *Synlett* **2019**, *30* 827-832.
